# SMPLIP-Score: predicting ligand binding affinity from simple and interpretable on-the-fly interaction fingerprint pattern descriptors

**DOI:** 10.1186/s13321-021-00507-1

**Published:** 2021-03-25

**Authors:** Surendra Kumar, Mi-hyun Kim

**Affiliations:** Gachon Institute of Pharmaceutical Science & Department of Pharmacy, College of Pharmacy, Gachon University, 191 Hambakmoeiro, Yeonsu-gu, Incheon, Republic of Korea

**Keywords:** Protein–ligand binding affinity, Interaction fingerprint pattern, Substructural molecular fragments, Random forest, Neural network, Featurization

## Abstract

In drug discovery, rapid and accurate prediction of protein–ligand binding affinities is a pivotal task for lead optimization with acceptable on-target potency as well as pharmacological efficacy. Furthermore, researchers hope for a high correlation between docking score and pose with key interactive residues, although scoring functions as free energy surrogates of protein–ligand complexes have failed to provide collinearity. Recently, various machine learning or deep learning methods have been proposed to overcome the drawbacks of scoring functions. Despite being highly accurate, their featurization process is complex and the meaning of the embedded features cannot directly be interpreted by human recognition without an additional feature analysis. Here, we propose SMPLIP-Score (Substructural Molecular and Protein–Ligand Interaction Pattern Score), a direct interpretable predictor of absolute binding affinity. Our simple featurization embeds the interaction fingerprint pattern on the ligand-binding site environment and molecular fragments of ligands into an input vectorized matrix for learning layers (random forest or deep neural network). Despite their less complex features than other state-of-the-art models, SMPLIP-Score achieved comparable performance, a Pearson’s correlation coefficient up to 0.80, and a root mean square error up to 1.18 in p*K* units with several benchmark datasets (PDBbind v.2015, Astex Diverse Set, CSAR NRC HiQ, FEP, PDBbind NMR, and CASF-2016). For this model, generality, predictive power, ranking power, and robustness were examined using direct interpretation of feature matrices for specific targets.

**Supplementary Information:**

The online version contains supplementary material available at 10.1186/s13321-021-00507-1.

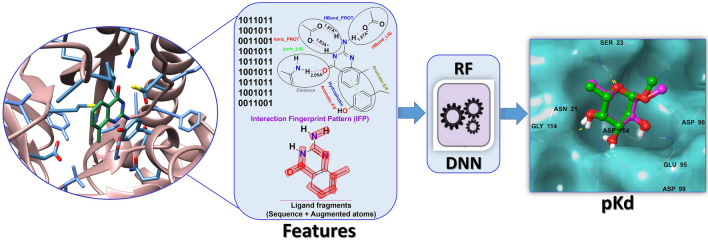

## Introduction

Protein–ligand binding in living organisms is a biological phenomenon that involves comprehensive processes such as molecular recognition and changes in protein conformation [1]. During drug development, any new molecules are evaluated empirically by measuring their binding strength to a protein target in vivo or in vitro. In contrast, computational ligand-based and target-based approaches are being used to predict the binding strengths of ligands [2–4]. In recent years, with advances in computational power, FEP (free-energy perturbation) methods [5, 6], MM–GBSA/MM-PBSA (molecular-mechanics–generalized Born surface area/molecular mechanics-Poisson–Boltzmann surface area) approaches [7–9], and molecular docking methods [10–13] have been widely used to accurately or relatively predict ligand binding poses and binding strengths with varying computational costs. Notably, for these predictions, physics-based, empirical, knowledge-based, and descriptor-based scoring functions have been used [14, 15]. These scoring functions are predetermined additive functional forms and are implemented in popular molecular docking programs such as AutoDock Vina [13], GlideScore [10], and Surflex-Dock [16]. Although these scoring functions were conveniently and widely used, they sometimes failed to discriminate binders from non-binders. Furthermore, although scoring functions as free energy surrogates for protein–ligand complexes have failed to provide collinearity, researchers hope for high correlations between docking scores and poses with key interactive residues. Thus, additional parameters have been included in the scoring functions of docking programs [17–19].

In recent years, machine learning (ML) and deep learning (DL) methods have achieved remarkable success in image and speech recognition, medical diagnosis, learning associations, classification, and regression analysis [20, 21]. ML methods have also been used to predict ligand binding strength by replacing linear scoring functions. These methods can be characterized by explicit and implicit features derived from protein, ligands, or protein–ligand pairs [22]. First, ligand binding strength depends on the vector summation of intermolecular interaction features such as hydrophobic, H-bond, π–π, cation–π, and charge interactions. Thus, several methods have been developed for extracting these features in different ways in the featurization process [23–25]. These features are either derived from an atom-centered or grid-based approach. For featurization, Gomes et al. [23] represented the structure of proteins and ligands as a combination of neighbor lists and atom types in their atom-centered approach for DL. Wallach et al. [26] and Ragoza et al. [27] represented the protein–ligand complex in a 3D-grid box to extract various interactions for the classification task. AtomNet [26], Pafnucy [28], K_DEEP_ [24], and RosENet [29] are some recent examples using an atom-based or grid-based approach to extract the features to build a convolutional neural network (CNN) model. Although the state-of-the-art DL predictors showed statistically significant and robust performance in their tested protein–ligand databases, interpreting the models is a challenge and a problem hampering further progress. Obviously, the features for affinity prediction is complex for describing atomic information in 3D space and the dimensions of 3D features is higher than data dimension such as other drug discovery prediction models (eg. 3D QSAR). Notably, the features embedded through a featurization process tend to show inscrutable patterns for human and the models fail to show how to understand their prediction for the decision-making of drug design (especially, preferred substructure of a ligand, its desirable binding pose, and the correlation with binding affinity). Therefore, a simple and interpretable featurization is required to explain an effective binding mode together with its predictive model that has reliable predictive power.

Second, diverse representations of protein–ligand interactions have been generated. Examples include algebraic graph theory (AGL-Score) [30], multiple layers of specific element pairs (OnionNet) [31] that shows local and non-local interactions (distance-dependent), protein–ligand extended connectivity fingerprint (PLEC-NN) [32], docking features (in ΔvinaRF_20_) [33], and predefined protein–ligand-interactions (ID-Score) [14]. Furthermore, molecular fingerprints, which are popular features in ligand-based virtual screening, have been applied to encode protein–ligand interactions. The fingerprint pattern can help to annotate protein families and their bound ligands. Recently, versatile tools, which capture protein–ligand binding interaction information as a fingerprint pattern with a binary string of 1 (if an interaction is present) or 0 (if an interaction is absent), have been developed, such as PLIP (Protein–Ligand Interaction Profiler) [34], IFP (Interaction Fingerprint) [35], SIFt (Structural Interaction Fingerprint) [36], and APIF (atom-pair-based interaction fingerprint) [37]. Among these tools, IFP has gained considerable popularity and suitability in drug-discovery experiments and has been used for (i) post-processing the docking result [38], (ii) prioritizing the scaffold pose [39], (iii) predicting the ligand pose [40], (iv) selecting the virtual hits [41], (v) comparing binding sites [42], and (v) designing target-oriented libraries [43]. The notable merit of IFP is its on-the-fly calculation of the interactions based on a certain set of rules (atom-types) and geometric relationships (distances and angles) between the interacting atoms of proteins and ligands [35]. Based on IFP, Chupakhin et al. built a neural network model to predict ligand-binding modes for three chosen targets (CDK2, p38-α, and HSP90-α) [42]. Unfortunately, their model was limited to these three target proteins.

Third, in addition to protein–ligand interaction features, some scoring functions use features from ligand structures (e.g., AutoDock [12], AutoDock Vina [13], and NNScore 2.0 [44]). Lin et al. reported that ligand features can reveal effective polypharmacological relationships between target proteins [45]. Boyles et al. predicted ligand-binding affinity using combined ligand features (derived from RDKit) and different scoring function (RF-Score, NNScore, and Vina) features [3]. Notably, various ligand shapes, from linear to multiple ring systems, can exhibit different binding affinity strengths even within one homologous protein class, suggesting the importance of ligand features in binding affinity prediction models [46, 47]. Thus, taking these into account, the combination of ligand features and interaction-based features can further improve performance of the scoring function.

**Fig. 1:**
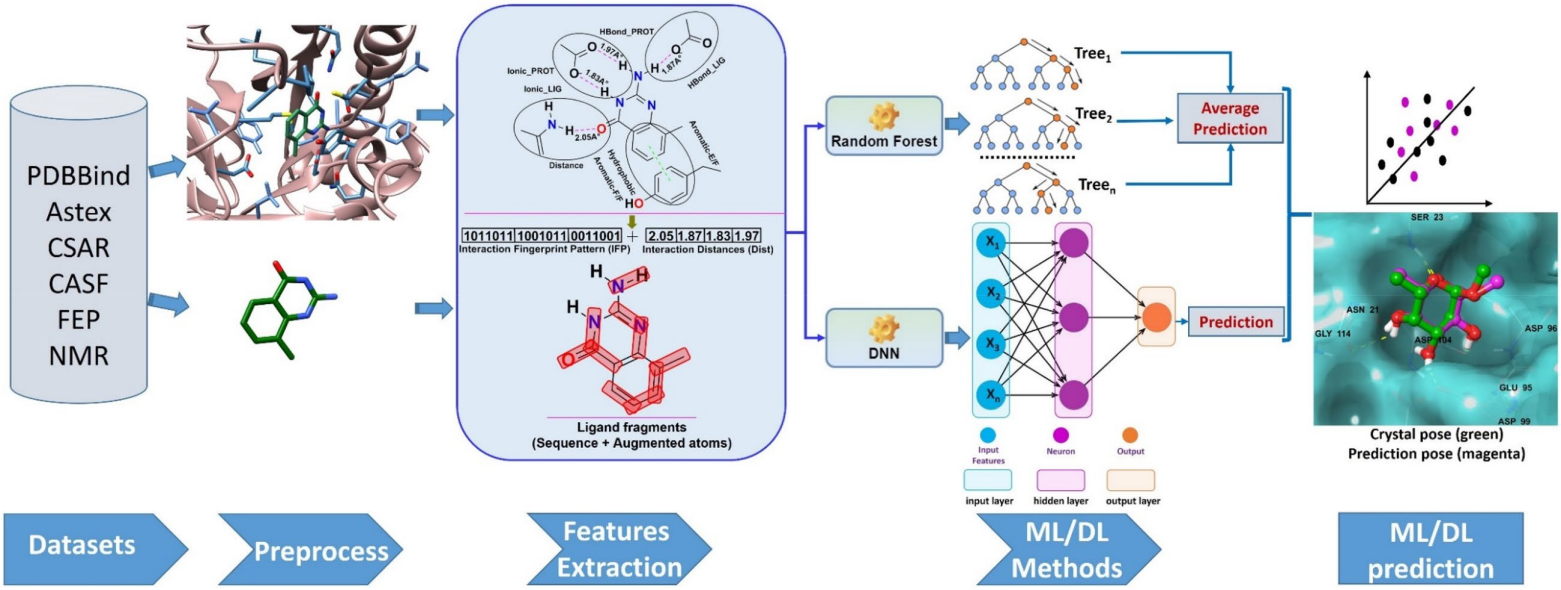
SMPLIP-Score workflow for binding affinity prediction. Publically available protein–ligand binding datasets were used to extract the information encoding the interaction fingerprint pattern (IFP), interaction distances (Int-Dist) from protein–ligand complex, and ligand fragments to which machine learning or deep learning method may be applied for affinity prediction

Based on these reported characteristics and drawbacks, we were motivated how to simplify usage, provide more interpretable features to explain protein–ligand binding directly, and provide ligand features for capturing polypharmacology. For this purpose, protein–ligand interaction-based fingerprint and ligand features were generated using IChem and SMF (substructural molecular fragment) tools, respectively. From these features, our best prediction model was realized in SMPLIP-Score (Substructural Molecular and Protein–Ligand Interaction Pattern-Score), as shown in Fig. 1. This work aimed to investigate three points: (a) the reliability of the predictive models that can be built from the IFP features of the protein–ligand complex and SMF of the ligand; (b) the efficiency of this featurization method based on complexity comparison within the SMPLIP features and between SMPLIP and the state of the art, and (c) the robustness or effectiveness of our models determined by comparing the predictive performances between simulated docking poses and experimental crystal poses.

## Materials and methods

### Dataset for learning

The protein–ligand database was downloaded from http://www.pdbbind-cn.org (PDBbind version 2015), which includes proteins and ligands in *.pdb and.mol2/.sdf file formats, respectively, each assigned with a PDB ID. The PDBbind database is a standard dataset that has previously been used to develop ligand-binding affinity prediction models [24, 25, 29, 31]. This dataset was categorized into three overlapping sets (i.e., general, refined, and core sets) with the total number of compounds for each set comprised of 11,908, 3706, and 195 proteins and ligands, respectively. These compounds were resolved by either X-ray or NMR methods with resolutions ranging from 0.75 to 4.60 Å, except for NMR solutions. The binding strength of each ligand to proteins was measured in IC_50_, K_d_, and K_i_ and reported in mM, μM, and nM, respectively. In the present work, we used the refined and core sets with the binding strength in only K_d_ or K_i_. The overlapping complexes between the refined and core sets were removed from the refined set. The refined set was randomly partitioned with a ratio of 80:20 into a train and valid set (a total of six subsets of the train and valid sets was created with a different random seed). Herein, the validation set was used to evaluate the robustness of the model's fit based on different random seeds. The training and validation were performed on a refined set and core set used to test the prediction performance of the developed models.

### Dataset preprocessing

The PDBbind dataset was cleaned and processed using the KNIME analytic platform [48]. The silent features of the KNIME analytic platform allow any user to perform several programming tasks using several nodes, without any previous background knowledge of programming languages. We created datasets cleaning the KNIME workflow that included nodes from the Community and Schrödinger suite [49], considering the protein path as input, iteratively reading the input PDB structure, adding H-atoms, correcting the bond order, removing water molecules from the protein files, and converting them into *.mol2 files. During preprocessing, the protein file with a resolution of < 2.5 Å was retained, so that a well-resolved protein structure could be used for feature construction (Additional file 1: Figure S1). After the preprocessing steps, a total of 3481 and 180 datasets were retained for featurization from the refined and core sets, respectively. Additional file 1: Figure S2 shows the characterization of the input PDBbind dataset after preprocessing.

### Feature construction

**Fig. 2:**
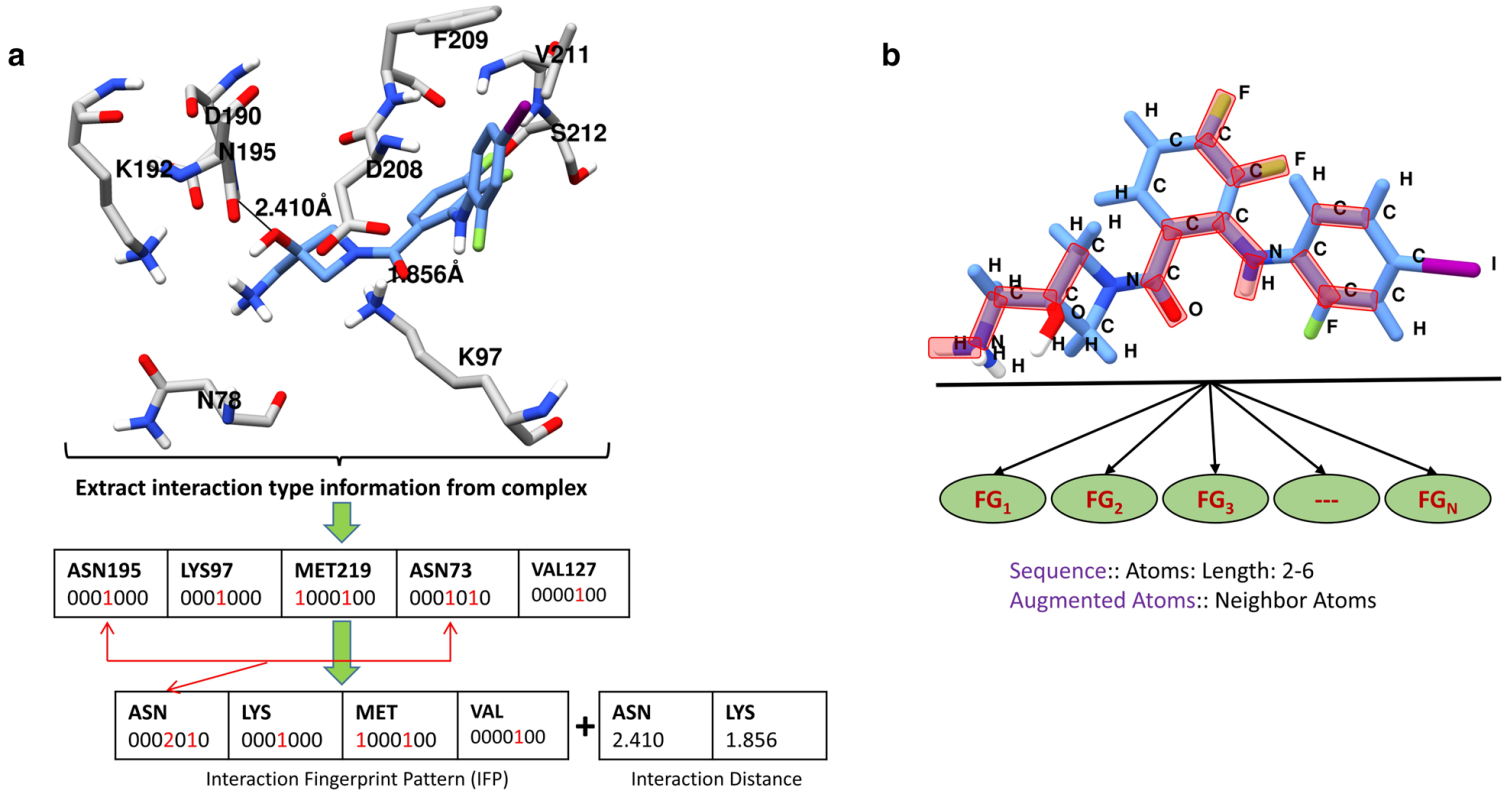
**a** Interaction fingerprint pattern (IFP) generation. The interaction information (seven type) for each amino acid was extracted and interaction types from the same amino acid was concatenation in a matrix with a fixed size of 140. Similarly, the observed interaction distances were measured. **b** The ligand fragmentation pattern (FG_1_, FG_2_, …, FG_N_) for the input ligand, which corresponds to atoms sequence and augmented atoms were calculated. (Refer feature constructions in Additional file 1 for detailed information)

We used two types of features that represent the ligand's active-binding site environment; one was based on the interaction pattern observed between a ligand and the protein's binding site amino acid residues, and the second utilized ligand fragments based on atoms and neighboring atoms. The IFP between each protein–ligand complex was calculated using the IChem tool, which is based on OEChem TK [35]. Using this tool, we extracted only the on/off information of the IFP and then created seven-bit-string values for interactive amino acid residues without any additional geometrical parameters (e.g., angle, dihedral angle, or distance) or location information of atoms/residues. These seven-bit-string values represented hydrophobic, aromatic face-to-face, aromatic edge-to-face, H-bond accepted by ligand, hydrogen bond donated by ligand, ionic bond with ligand negatively charged, and ionic bond with ligand positively charged under standard geometric rules. Therefore, if any amino acid residues within the binding pocket formed any interaction with the ligand atoms, then the respective interaction was assigned a value of 1; otherwise, it was assigned a value of 0. Moreover, if two arginine residues respectively were hydrogen bonded with the ligand in a protein–ligand complex, the ‘ARG_HBond_LIGAND’ feature was assigned the integer 2. Considering the 20 standard amino acids in biological systems and the 7 bits of interaction information, a matrix of 20 × 7 = 140 was constructed. Notably, favorable interactions were only formed when two interacting atoms from proteins and ligands were in close proximity to each other. These interactions were distance-dependent and governed by spatial and geometric rules. Thus, the interaction distance information from the protein–ligand interaction pair was also extracted and combined with an interaction fingerprint pattern that equaled a total of 280 lengths (see detailed feature constructions in Additional file 1). The refined and core sets of the PDBbind database (release 2015) contain more than three thousand co-crystal ligands, which are structurally diverse and vary in shape and size with ligand lengths up to 47 Å. Thus, considering the different shapes and sizes of ligands, we used the SMF program to calculate the substructural fragment descriptors for the ligands [50]. Two types of substructural fragment descriptors were calculated: the sequence of atoms with a path length of up to six atoms and atoms with their neighbors. Both types contributed a total of 2282 substructural fragment descriptors (see detailed feature constructions in Additional file 1). The feature construction method used in this study is shown in Fig. 2.

### Machine learning and deep learning methods

In recent years, the various branches of artificial intelligence (i.e., ML and DL) have gained wide applicability in drug design and discovery, which includes predicting the numerous properties of a set of ligands or predicting affinity of the bound ligand in the protein binding pocket. Based on successful prediction performances, in the present study, we used random forest (RF) and deep neural network (DNN) as the ensemble learning method and DL method, respectively.

#### Ensemble learning

Ensemble learning-based methods combine several models, which were built individually to improve prediction performance. The general ensemble learning methods can be divided into two categories: bagging and boosting. Bagging is also called bootstrap aggregation, where multiple sample sets are produced and these sets are trained by individual learners. The main advantage of using bagging algorithms is that they decrease the prediction variance of the model and improve the accuracy of the ensemble. In this study, we used the RF as a bagging algorithm to build the regression model. RF is an ensemble of the decision tree (B) $\{ T_{1} (X),....,T_{B} (X)\}$, as a base learning model, where $X = \{ x_{1} ,...,x_{p} \}$ is a p-dimensional vector of molecular properties. Ensemble learning produces output $\{ \widehat{Y}_{1} = T_{1} (X),....,\widehat{{Y_{B} }} = T_{B} (X)\}$, where $\widehat{{Y_{b} }},b = 1,...,B,$ is the prediction for a molecule by the *b*th tree. The outputs obtained from all trees are aggregated to produce one final prediction for each molecule [51]. In regression modeling, this is the average of the individual tree predictions. The scikit-learn package (version 0.22) [52] was used to train and build the RF model. To build the models, different parameters from the RF were used (n_estimators = 100, 200, 300, 400, 500 and max_features = ‘auto’ and ‘sqrt’). The random_state parameters were fixed to different seed numbers during training to reproduce the statistical results (Additional file 1: Table S1).

#### Deep neural network

In recent decades, DL has been used for image classification, video processing, speech recognition, and natural language processing. In addition, these methods have been used in drug design and discovery applications over the last few years [53, 54]. A typical DNN method uses an artificial neural network (ANN) to make a decision or solve the problem. The standard DNN architecture includes the input layer, hidden layers, and output layers. In this study, we used a DNN to build the model and perform the predictions. The DNN model was trained using Keras (version 2.2.4) with the TensorFlow backend module [55]. The DNN training utilized a sequential model, which was initialized followed by four dense layers with 400, 200, and 100 units. Finally, the output layer was connected to one neuron to produce the predicted pK_d_. During the DNN training, early stopping criteria (Δ_loss_), dropout, batch normalization, and L2 regularization were adopted to avoid over-fitting of the DNN model. The DNN model was trained with tunable parameters that included dropout regularization (0.1 to 0.6), alpha (0.1 to 1.0), and batch sizes of 64, 128, and 256. During learning, the best model was obtained as the learning entered an over-fitting stage, which was based on a modified loss formula (LOSS) adopted from Zheng et al. [31].

$${\text{LOSS}} = \alpha \left( {{1} - {\text{PCC}}} \right) + \left( {{1} - \alpha } \right){\text{ RMSE}}$$

where PCC is the Pearson correlation coefficient and RMSE is the root mean square error. Furthermore, some additional parameters, such as a learning rate of 0.001, decay constant of 1e^−6^, and momentum of 0.9, were kept constant during the learning. Rectified linear units (ReLUs) were used at each layer as activation functions, and the stochastic gradient descent (SGD) optimizer was selected to search for optimal weights in the model. The DNN script was adopted from the work of Zheng et al. [31].

#### Evaluation metrics

The quality and performance of each ML or DL model were assessed using various evaluation metrics, including RMSE, mean absolute error (MAE), and PCC. The detailed information is as follows:

$$RMSE = \sqrt {\frac{1}{N}\sum\limits_{i = 1}^{N} {(BA_{predict} } - BA_{true} )^{2} }$$

where RMSE measures the average magnitude of the error and represents the square root of the average of squared differences between the predicted and experimental values.

The MAE is another evaluation metric that differs from RMSE, as MAE is the average of the summed absolute differences of the predicted and experimental values.

$$MAE = \frac{1}{N}\sum {|BA_{predict} - BA_{true} |}$$

The PCC was used to estimate the linear relationship between the predicted and experimental values. This metric also assesses the scoring function ability of the model.

$$PCC(R) = \frac{{\sum {[(BA_{predict} - \overline{BA}_{predict} )(BA_{true} - \overline{BA}_{predict} )]} }}{{(SD_{{\overline{BA}_{predict} }} )(SD_{{\overline{BA}_{true} }} )}}$$

#### Benchmark datasets for evaluation

We used five different benchmark datasets to assess the accuracy and efficiency of SMPLIP-Score. Previously, these datasets were used by many researchers to measure the quality and performance of their ML/DL/CNN models.

##### Astex diverse datasets

These datasets comprised diverse and high-quality protein–ligand pairs. Mooij et al. [56] manually curated these datasets to validate the protein–ligand docking program. After checking and comparing the overlapped protein–ligand pairs, 15 protein–ligand pairs remained for further processing.

##### Community structure–activity resource (CSAR) datasets

These in-house datasets were collected and managed by the University of Michigan. Among the CSAR datasets, we used the CSAR-HiQ-NRC (Set01 and Set02) benchmark dataset from http://csardock.org. The original input dataset contained 176 and 167 protein–ligand pairs for Set01 and Set02, respectively, with binding affinity data in K_d_/K_i_. After comparing and excluding the overlapped protein–ligand pairs from the refined set, a total of 56 and 64 pairs remained in Set01 and Set02 for further processing, respectively.

##### Comparative assessment of scoring functions (CASF) datasets

The CASF datasets are part of the PDBbind dataset and consist of a collection of high-quality protein–ligand complexes that are provided to assess scoring functions. We used CASF-2016 (http://www.pdbbind-cn.org/casf.php), which is comprised of 285 protein–ligand pairs with their experimental activity in K_d_/K_i_. A total of 122 protein–ligand pairs were selected after excluding overlapped pairs from the training set.

##### FEP dataset

This dataset is comprised of eight targets (BACE, CDK2, JNK1, MCL1, p38, PTP1B, thrombin, and Tyk2) and contains 199 compounds selected from the literature by Wang et al. [6] in order to predict relative ligand-binding affinities using the FEP method. While there are 199 compounds for eight targets, binding affinities in K_i_ have only been reported for five targets (BACE, MCL1, PTP1B, thrombin, and Tyk2). Therefore, in our work, we excluded CDK2, JNK1, and p38 from our FEP dataset. The remaining five targets were not part of the refined set, so all the reported compounds were selected for the BACE, MCL1, PTP1B, thrombin, and Tyk2 targets (36, 42, 22, 11, and 16 compounds, respectively).

##### NMR PDBbind dataset

We also tested the performance of the SMPLIP-Score on protein–ligand pairs that were resolved by NMR. The refined and core sets lacked NMR-resolved structures; thus, these structures were obtained from the general set. A total of 191 protein–ligand pairs were selected from the general set.

## Results and discussion

We built predictive models using IFP and SMF features, optimized the models, and evaluated their predictive power on the benchmark dataset. We further tested the robustness and effectiveness of our best models using the poses (input features) derived from the molecular docking simulations. We used the RF as an ensemble method and DNN as a DL method to build the predictive models, SMPLIP-RF and SMPLIP-DNN.

### SMPLIP-RF model

**Fig. 3:**
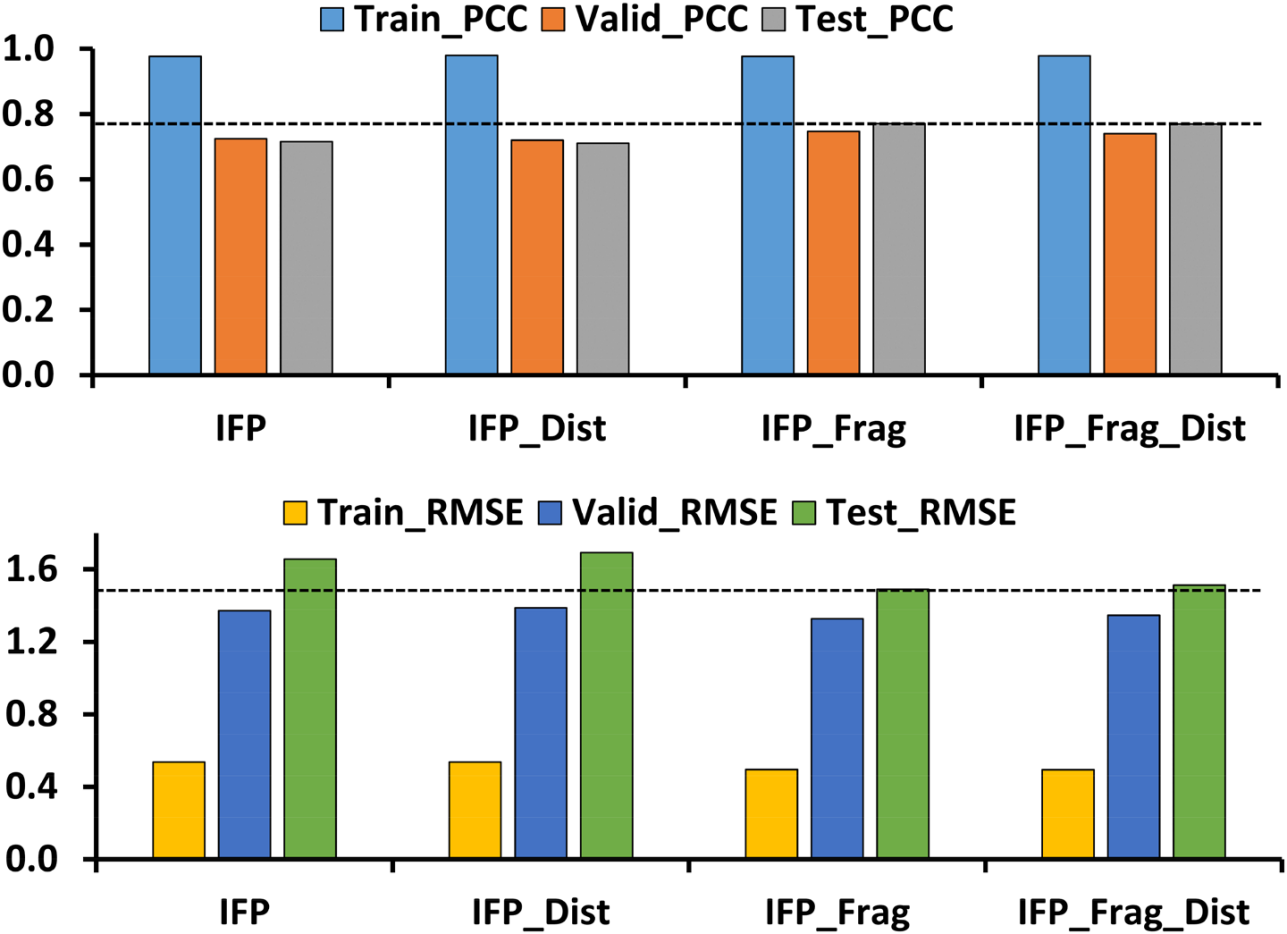
The bar plot for statistical comparison from different feature (s) combination. The PCC (Pearson-correlation-coefficient) and RMSE (root-mean-squared-error) were compared from the train, valid, and test data

We first investigated the use of a single feature or combined features on the prediction performance of the model using the RF method. We trained six different models (with a different random seed) and different combinations of feature(s), n_estimators, and max_features were used for each model. All the parameters were set to default in the RF models, except max_features (‘auto’ or ‘sqrt’) and n_estimators (100, 200, 300, 400, or 500). Additional file 1: Table S1 and Figures S3–S10 report the statistical results, and the best model (based on the lowest RMSE reported on the test data) was selected for each feature or combined features after comparing all the statistical results for the different sets (Table 1 and Fig. 3).

**Table 1:** The statistical performance of SMPLIP-RF models on PDBbind (Release 2015) according to different features compositions

Features	Train				Valid				Test			
	RMSE	MAE	PCC	p_Value	RMSE	MAE	PCC	p_Value	RMSE	MAE	PCC	p_Value
IFP	0.537	0.420	0.977	0	1.372	1.066	0.724	4.17E-115	1.656	1.349	0.716	1.49E-29
IFP + Int-Dist	0.536	0.422	0.980	0	1.387	1.093	0.720	2.08E-112	1.692	1.388	0.711	5.15E-29
IFP + Frag	**0.496**	**0.381**	**0.977**	**0**	**1.327**	**1.035**	**0.747**	**2.62E-125**	**1.489**	**1.227**	**0.771**	**8.71E-37**
IFP + Int-Dist + Frag	0.494	0.382	0.978	0	1.346	1.054	0.740	8.07E-122	1.512	1.244	0.770	1.43E-36

The Refined set (n = 3481) used for training and validation, and core set (n = 180) as a test data. The boldface represents the model with better statistics from different features combination and max_features options

*RMSE* root-mean-square-error, *MAE* mean absolute error, *PCC* Pearson correlation coefficient, *p_value* p_value for statistical significance

Comparing the predictive power of the test data (the RF models with different combinations of features), the features from IFP + Frag at n_estimators = 100 and max_features = ‘auto’ showed the best performance with the lowest RMSE (1.489) and high PCC (0.771) at a significance value of 8.71E^−37^ (refer to Additional file 1 for a detailed discussion). This model (IFP + Frag: 0.771) had a higher PCC and comparable performance to some models reported by Boyles et al. (Vina + RDKit: 0.749; RF-Score + RDKit: 0.778), but slightly lower than that from other models (RF-Score-v3 + RDKit: 0.780; NNScore 2.0 + RDKit: 0.786) [3]. Notably, this result suggests that facile, recognizable SMPLIP features (especially IFP + Frag) can show sufficient predictive power that is never inferior to known scoring using complex features such as atom-centered or grid-based features. Moreover, we built a null model and compared its statistics with the IFP and IFP + Frag features (Additional file 1: Table S2). The statistical metrics revealed that the addition of IFP features significantly reduced the overall prediction error.

### SMPLIP-DNN model

We further evaluated the prediction performance of SMPLIP features using the DNN method. For the SMPLIP-DNN model, we used the same set of training, validation, and test data that had been used to compare the statistical performance of the RF model. The predictive model was built in our DNN training with screened hyperparameter values for dropout, alpha, and batch size. The models for each feature or combined features with optimized hyperparameter values are shown in Additional file 1: Table S3 and Figures S11–S14. In Additional file 1: Table S3, the best model at each epoch was based on the modified loss formula, which prevents overfitting of the model. The best model for each feature combination is shown in Table 2.

**Table 2:** The statistical performance of SMPLIP-DNN models on PDBbind (Release 2015) according to different features compositions

Features	Train			Valid			Test		
	LOSS	RMSE	PCC	LOSS	RMSE	PCC	LOSS	RMSE	PCC
IFP	0.563	0.871	0.899	1.019	1.483	0.678	1.032	1.538	0.726
IFP + Int-Dist	0.472	0.990	0.873	0.775	1.468	0.687	0.805	1.582	0.713
IFP + Frag	**0.209**	**0.595**	**0.956**	**0.631**	**1.402**	**0.699**	**0.646**	**1.530**	**0.733**
IFP + Int-Dist + Frag	0.212	0.403	0.979	0.834	1.400	0.733	0.923	1.559	0.714

The Refined set (n = 3481) used for training and validation, and core set (n = 180) as a test data. The boldface represents the model with better statistics from different features combination

Notably, in the RF model, the IFP + Frag features possess high predictive power for the test data, while in the DNN model, these features did not dramatically improve the test data (PCC: 0.733; RMSE: 1.530). For the IFP + Frag features, the best model was obtained with a batch size of 64, dropout of 0.1, and alpha value of 0.7 at 129 epochs (Additional file 1: Figure S15).

### Comparison of predictive performance with known models

**Fig. 4:**
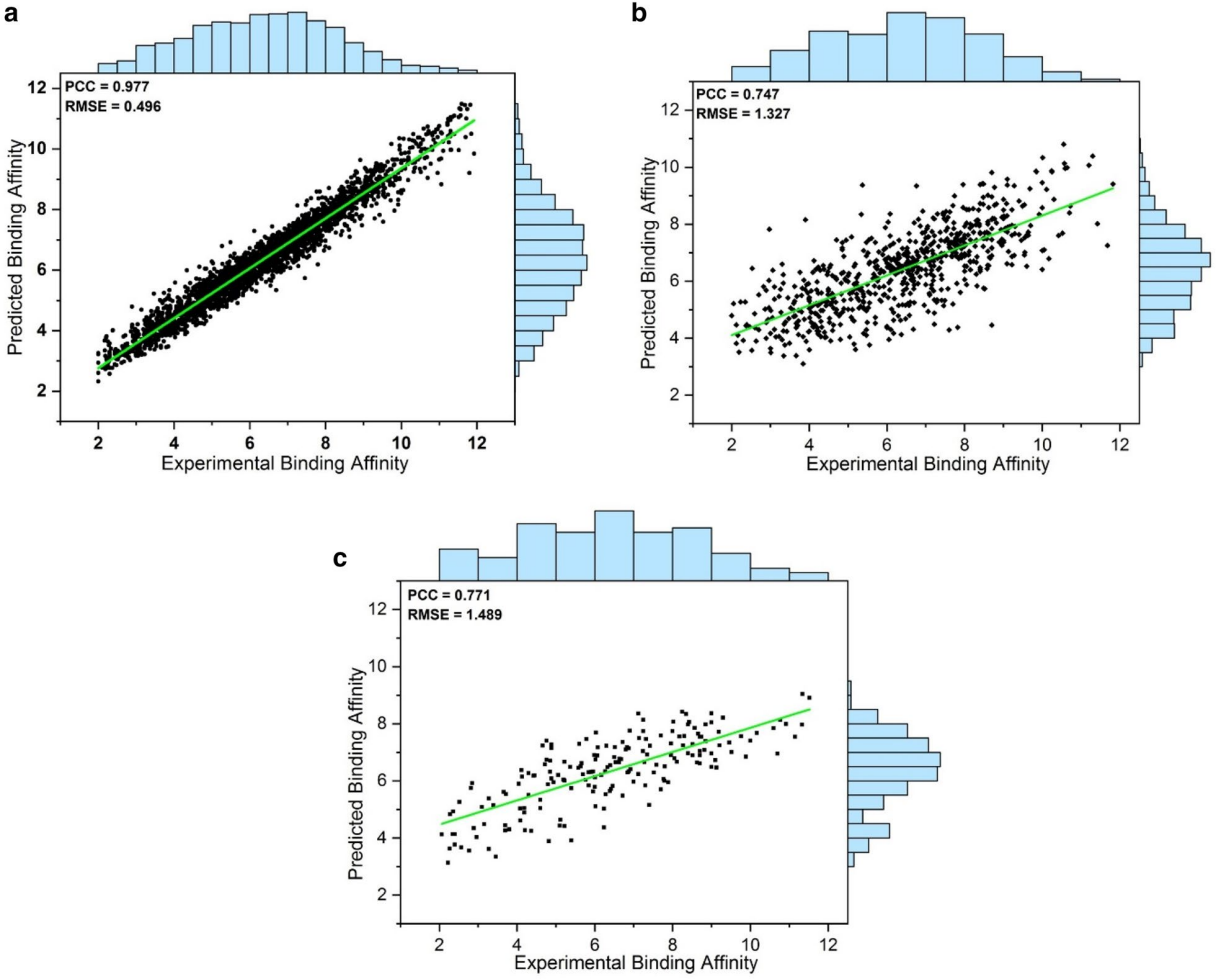
2D-Scatter plot between predicted and experimental of SMPLIP-RF (IFP + Frag) for **a** training, **b** valid, and **c** test data

We built the SMPLIP-RF and SMPLIP-DNN models to predict ligand-binding affinities. Under our investigated conditions (different partitioning of the dataset, different hyperparameter options, and chosen features), all models presented statistical reliability with the distribution of PCC and RMSE values from independently trained models (Additional file 1: Tables S1 and S2) and with their variance analysis (Additional file 1: Table S4). Although the IFP + Frag feature-based model outperformed on predicting binding affinity in both the RF and DNN methods, the statistical performance (PCC and RMSE) of the RF model on the test data was better than the DNN model. Figure 4 shows the correlation of predicted and experimental binding affinities from SMPLIP-RF (IFP + Frag) as a scatter plot for the training, valid, and test data.

Consequently, the best SMPLIP-Score motivated us to assess its scoring and ranking power on other predictive models with different featurization methods. For this purpose, the performance of the SMPLIP-Score was further compared with that of other state-of-the-art models (Table 3). First, the SMPLIP-Score was compared with the featurizer methods of Gomes et al. [23], specifically atomic convolutional neural network (ACNN), GRID-RF, GRID-NN, GCNN, extended connectivity fingerprint (ECFP)-RF, and ECFP-NN. Their featurizer performs a 3D spatial convolution operation to learn atomic-level chemical interactions from a protein–ligand pair. Using these featurization methods, they performed poorly on the core set. Statistical comparison revealed that our SMPILP-Score performed better than the GRID-RF and ACNN models (Table 3). Second, the algebraic topology featurization of Cang et al. [57] was compared with the SMPLIP-Score. Despite their slightly higher PCC values than those of the SMPLIP-Score, the RMSE of the SMPLIP-Score was distinctly lower than their RMSE, suggesting better performance. Third, the rigidity index score (RI-Score) of Nguyen et al. [58] produced a higher RMSE using core data as the test set. Dziubinska et al.’s 3D grid-based CNN model, the Pafnucy model [28], was also inferior to our SMPLIP-Score model.

**Table 3:** Performance comparison of SMPLIP-Score with reported models on the PDBbind v.2015 dataset

ML/DL Method	Core Set	Refs
SMPLIP-Score	0.771 (1.489)	–
ACNN	0.669	Gomes et al. [23]
TopBP-ML	0.797 (1.99)	Cang et al. [57]
TopBP-DL	0.799 (1.91)	
RI-Score	0.782 (2.051)	Nguyen et al. [58]
Pafnucy	0.70 (1.62)	Stepniewska Dziubinska et al. [28]
PLEC-Linear	0.757 (1.47)^a^	Wójcikowski et al. [32]
PLEC-NN	0.774 (1.43)^a^	Wójcikowski et al. [32]
OnionNet	0.782 (1.503)	Zheng et al. [31]
RF-Score-v3	0.74 (1.51)^a^	Wójcikowski et al. [59]
X-Score	0.614 (1.78)^a^	Khamis et al. [60]
Autodock Vina	0.54 (1.90)^a^	Gaillard et al. [61]
Autodock	0.54 (1.91)^a^	

Pearson correlation coefficients with RMSE in parentheses for predictions by different methods

*SMPLIP-Score* interaction fingerprint pattern and Ligand Fragment-based random forest (RF) model, *GRID-RF* grid featurizer based Random Forest; *ACNN* Atomic Convolutional Neural Network model, *TopBP* Topology based model, *RI-Score* Rigidity Index based score

^a^Values in parenthesis represent the standard deviation (SD)

In turn, Wójcikowski et al.’s [32] circular fingerprint featurization (PLEC-linear and PLEC-NN) with different depth levels (protein depth of 5 and a ligand depth of 1) and the SMPLIP-Score produced comparable performances despite the fact that the latter uses fewer features (2422) than the PLEC (65,536) models do. Furthermore, the SMPLIP-Score is readily understandable based on the interpretability of the embedded feature matrix because SMPLIP feature is mimetic to human’s recognition on binding poses, which didn’t use either any geometrical or topological information. In detail, only existence information of residue types (20 amino acids), interactions (seven types), and ligand substructures (2282 fragments) were directly embedded in SMPLIP while the location numbers of bits (of the radial fingerprints) were embedded in PLEC [32]. Moreover, OnionNet [31], a popular method based on a feature matrix derived from rotation-free element pair-specific contacts between protein and ligand, achieved a slightly higher RMSE than the SMPLIP-Score based on the core set. Consistently, the SMPLIP-Score even outperformed popular ML scoring functions, such as RF-Score-v3 [59], X-Score [60], AutoDock Vina [61], and AutoDock [61] using the PDBbind v.2013 core set. Notably, all the models/methods compared here are different in terms of their featurization processes and require state-of-the-art architecture (ML/DL/CNN) to achieve predictive power.

### Complexity comparison of SMPLIP with information cost

Obviously, time complexity can be determined by the dimensions of features (*d*), the number of data points (*n*), and learning parameters such as the number of bagged trees (*t*) of RF, and the number of layers (*l*) and number of nodes of DNN. As shown in the big *O* in Table 4, feature size (*d*) can be dominant in some model, which shows the dimensions of features are larger than the number of data points. To our best knowledge, almost all binding affinity prediction models also show *d* >> *n* for respective benchmark datasets. In the cases, an efficient feature size need to be considered for the complexity. Similarly, ${O}_{RF}$, can depend on the dimensions of sampled features (*d*ʹ) under the conditions of similar data (*n*ʹ) and hyperparameters (*t*). Thus, because the optimal number of PLEC features was 65,536 and 27-fold larger than the best SMPLIP feature, the PLEC-linear is 27-fold more complex than SMPLIP-linear under the same learning condition in Table 4. Expectedly, this difference in complexity between PLEC-RF and SMPLIP-RF is reduced from 27 folds into 5.2 folds (${d}^{^{\prime}}: sqrt$). In cases of DNN models, the number of learning parameters is overwhelmingly *d* or *n*, as shown in the ${O}_{DNN}.$ When we measured run times, the run times followed the big *O*. ‘IFP’, ‘IFP + Int-Dist’, ‘IFP + Frag’, and ‘IFP + Int-Dist + Frag’ showed their run times proportional to the dimensions of features under RF method. In the case of space complexity, the dimension of features didn’t make an effect on space complexity and different learning parameters show also trivial difference.

**Table 4:** Complexity comparisons of SMPLIP-Score

Models	Features	No of features	Learning parameters	Run time (s)^a^	Complexity^a^	Memory usage (max)^b^
SMPLIP-RF	IFP	140	max_features (*d*ʹ) = ‘auto’ or ‘sqrt’ n-estimators (*t*) = 100 to 500	2.82	$O(t*O(d{^{\prime}}*n{^{\prime}}*{\log}(n{^{\prime}}))$	55.24
	IFP + Int-Dist	280		6.55		56.621
	IFP + Frag	2422		36.86		57.18
	IFP + Int-Dist + Frag	2562		40.37		57.17
SMPLIP-DNN	IFP	140	159,601	137.96	$$O(d*\mathrm{n}*layers*nodes)$$	130.49
	IFP + Int-Dist	280	215,601	161.83		131.73
	IFP + Frag	2422	1,072,401	295.79		130.93
	IFP + Int-Dist + Frag	2562	1,128,401	495.57		131.18
SMPLIP-Linear	IFP + Frag	2422	Loss = huber, penalty = elasticnet, max_iter ($${t}_{i})$$=100	1.54	$$O({t}_{i}*d*n)$$	57.66
PLEC-Linear^c^	PLEC FP	65,536	Loss = huber, penalty = elasticnet, max_iter ($${t}_{i})$$=100	–	$$O({t}_{i}*d*n)$$	–

^a,b^ The run time and memory usages were computed on system (Intel Xeon CPU E5-2650) using PDBbindv.2015-refined set

^a^The comparison of time complexity according to chosen features or learning condition

^b^The comparison of space complexity according to chosen features or learning condition

^c^The data was gained from the original article of PLEC [32]

In addition to computational complexity, information cost (or information complexity) [62] of SMPLIP could be considered through the comparison of used information quantity. At this time, we considered Shanon information cost, *H*(*X*): = **E**_*x∼X*_[*− *log_2_ *P*_*X*_(*x*)]. Because the expected value, **E** is the summation of each probability of each feature ($-\sum_{i=1}^{d}{p}_{i}{\mathrm{log}}_{2}{p}_{i}$), the information cost, *H* increases according to the dimensions of features. Thus, low dimensions of features show low information cost. Similarly, ‘IFP’ feature of SMPLIP is less complex than ‘IFP + Frag’ and ‘IFP + Frag’ of SMPLIP also is less complex than PLEC in the view of information cost. Notably, both IFP and Frag in SMPLIP only captured on/off information without either geometric parameters (e.g., angle, dihedral angle, or distance) or location information of atoms/residues. Despite of low information cost, SMPLIP showed comparative predictive performance to state-of-the art methods and SMPLIP-RF was the most cost-effective among tested learning conditions. Furthermore, the extraction of interesting features through simple counting approximates human recognition and data treatment. In other words, SMPLIP can provide direct interpretation, which does not require additional feature importance analysis such as the distribution of weights in learning architectures [28] or visualized contour functions (Ragoza et al.) [27]. When specific targets or chemical scaffolds need to be understood for drug design, the data matrix of SMPLIP can directly compare docking poses based on the simple counting. In brief, SMPLIP-RF demonstrated its cost-effectiveness based on the following views: (1) low computational complexity, (2) low information cost (presence or absence), (3) direct interpretation from embedded feature matrix to docking pose without additional analysis, and (4) predictive power comparable to state-of-the-art models.

### Generalization of SMPLIP-Score evaluated through benchmark datasets

The generalization of the SMPLIP-Score was tested using additional benchmark datasets. These datasets were previously used by other researchers to evaluate the performance of their ML/DL/CNN models. The benchmark datasets used here belong to the Astex Diverse, CSAR NRC HiQ, CASF-2016, FEP, and PDBbind NMR datasets. SMPLIP-RF based on IFP + Frag features with n_estimators = 100 and max_features = ‘auto’ was used for generalized evaluation of the benchmark datasets. Table 5 lists the comparative assessment of the calculated scoring metrics (PCC, RMSE, and Spearman's rank correlation coefficient (Sp)) for these datasets. The scatter plot for the predicted binding affinity against the experimental binding affinity for these benchmark datasets is shown in Additional file 1: Figure S16. The original Astex Diverse dataset consisted of 93 protein–ligand pairs and after removing the overlapped pairs from the PDBbind refined set, a small set of 15 protein–ligand pairs remained for prediction measurements. We achieved PCC, Sp, and RMSE values of 0.724, 0.764, and 1.177 for this dataset, respectively. The SMPLIP-Score achieved a slightly lower scoring than DeepAtom but better scoring and ranking than RF-Score, Pafnucy, Res4HTMD, and RosENet using the Astex Diverse dataset with previously reported models [25, 28, 29, 63].

**Table 5:** The prediction performance on benchmark datasets and statistical comparison of SMPLIP-Score with reported models

Datasets	Models	SETS	PCC	RMSE	MAE	p_Value	Sp	Refs
Astex Diverse Set	SMPLIP-Score	–	0.724	1.177	0.938	0.002	0.764	–
CSAR NRC HiQ		Set01	0.785	1.903	1.500	8.03E−13	0.761	
		Set02	0.803	1.475	1.134	1.54E−15	0.823	
FEP		BACE	0.239	0.639	0.505	0.160	0.250	
		MCL1	0.077	1.045	0.797	0.629	0.146	
		PTP1B	0.634	0.768	0.536	0.002	0.680	
		Thrombin	− 0.645	0.962	0.780	0.321	− 0.536	
		Tyk2	0.469	0.859	0.655	0.078	0.546	
PDBbind NMR		–	0.209	1.857	1.552	0.004	0.234	
Astex Diverse Set	DeepAtom	–	0.768	1.027	0.714	–	–	Li et al. [25]
	RF-Score	–	0.710	1.144	0.891	–	–	Ballester et al. [63]
	Pafnucy	–	0.569	1.374	1.110	–	–	Stepniewska Dziubinska et al. [28]
	Res4HTMD	–	–	1.54	–	0.07	0.41	Hassan Harrirou et al. [29]
	RosENet	–	–	1.84	–	0.21	0.29	
CSAR NRC HiQ	K_DEPP_	Set01	0.72	2.09	–	–	–	Jiménez et al. [24]
		Set02	0.65	1.92	–	–	–	
	Res4HTMD	Set01	–	1.75	–	2E−15	0.84	Hassan Harrirou et al. [29]
		Set02	–	1.34	–	3E−13	0.83	
	RosENet	Set01	–	1.71	–	2E−17	0.87	
		Set02	–	1.38	–	2E−14	0.85	
FEP	K_DEEP_	BACE	− 0.06	0.84	–	–	–	Jiménez et al. [24]
		MCL1	0.34	1.04	–	–	–	
		PTP1B	0.58	0.93	–	–	–	
		Thrombin	0.58	0.44	–	–	–	
		Tyk2	− 0.22	1.13	–	–	–	
	Res4HTMD	BACE	–	1.27	–	0.26	− 0.19	Hassan Harrirou et al. [29]
		MCL1	–	1.1	–	2E−3	0.45	
		PTP1B	–	0.88	–	6E−3	0.55	
		Thrombin	–	0.83	–	0.16	0.45	
		Tyk2	–	0.76	–	2E−3	0.71	
PDBbind NMR	RosENet	–	–	1.37	–	–	0.56	

*Models* ML/DL method used to build the ligand binding affinity prediction model, *RMSE* root-mean-square-error, *MAE* mean absolute error, *PCC* Pearson correlation coefficient, *p_value* p_value for statistical significance, *Sp* Spearman correlation coefficient, *RF-Model* RF parameters includes: n_estimators = 500; max_features = “AUTO”

The second selected benchmark (the CSAR NRC HiQ dataset) consists of both Set01 and Set02. These sets were also used for docking program validation. After checking the overlapped protein–ligand pairs, 56 and 64 pairs were left in Set01 and Set02, respectively, for predictive evaluation. Compared with K_DEEP_, a CNN model, the SMPLIP-Score has better performance. However, a slightly lower performance was observed in Sp when compared to models from Res4HTMD and RosENet in predicting the binding affinity of the CSAR NRC HiQ sets [24, 29].

Another benchmark dataset, popularly known as the FEP dataset, was selected from the work of Wang et al. [6]. This dataset was used to predict the relative binding potency using a modern free-energy calculation protocol and forcefield. This dataset was comprised of ligands from BACE, MCL1, PTP1B, thrombin, and Tyk2 targets. Notably, except for thrombin, all prediction performances from SMPLIP-Score were positive, and the prediction ranking was PTP1B > Tyk2 > BACE > MCL1 > thrombin. Comparison of the prediction results with those of other methods (i.e., K_DEEP_ and Res4HTMD) revealed that for all of the FEP targets, SMPLIP-Score performed better than the K_DEEP_ model, while for BACE and PTP1B targets, Res4HTMD performed better [24, 29].

Finally, we further predicted the ligand-binding affinity of the dataset for which different experimental techniques have been used. A total of 191 protein–ligand pairs derived from the NMR method were selected and predicted for their ligand-binding affinities. Compared with the RosENet model, our model did not predict well the ligand-binding affinity derived from the NMR method [29]. Overall, SMPLIP-Score performed very well in benchmark evaluations in predicting the ligand-binding affinities of most cases, which affirms the reliability of our model for binding affinity predictions of diversified datasets, and it can be used further for virtual drug screening.

### Ranking power on the benchmark dataset

SMPLIP-Score was further assessed for its ranking power, as indicated by the value of Sp for the CASF-2016 benchmark dataset. The CASF benchmark dataset consists of high, medium, and low active crystal and locally optimized poses from each protein target with a cluster number. Here, using these poses, two types of rankings were calculated; first, we calculated rankings of all Sp for the reduced set, and second, we calculated the ranking of individual Sp for each cluster, followed by an average of all clusters. The calculated PCC and Sp values for the CASF benchmark dataset are listed in Table 6. For this dataset, the PCC on the crystal pose and locally optimized pose remained the same, while there was a small increase in RMSE. Although the differences in root-mean-square deviations (RMSDs) for crystal and locally optimized poses were not large, such small differences in prediction/error were expected because any ML/DL model is sensitive to input features. Notably, the prediction results for the crystal pose and minimized pose were not even > 0.1, indicating that our model was less sensitive to the minimized pose than to the crystal pose. Additionally, the ranking metrics indicated that the average value of Sp for all the clusters was lower than the overall Sp. This suggests that, while each cluster has high, medium, and low active compounds, the difference in activity in some clusters is not large, rendering the ranking of compounds prone to change by prediction error. We further compared the prediction performance of the SMPLIP-Score on other ML/DL models. The statistical results from models based on the AutoDock Vina, ΔSAS (buried percentage of the solvent-accessible surface) [64], and Δ_Vina_RF_20_ [33] functions are listed in Table 6, which shows that the input features used in all three ML/DL methods are different from our features. Nevertheless, the performance of our model was greater than the AutoDock Vina and ΔSAS functions, but not Δ_Vina_RF_20_. Notably, Δ_Vina_RF_20_ uses descriptors derived from the AutoDock Vina interaction, ligand-dependent, and bSASA terms, which can result in superior performance with the benchmark dataset. Nevertheless, our prediction model uses protein–ligand interaction fingerprint and ligand-dependent features for predicting ligand-binding affinities, and we expect future additional interaction terms, such as desolvation, entropy effects, and surface and shape matching properties, may further improve the prediction performance.

**Table 6:** The evaluation of the CASF-2016 dataset

Models	SETS	PCC	RMSE	Spearman (Sp)		Refs
				All	Cluster average	
SMPLIP-Score (IFP + Frag Features)	Crystal-reduced	0.775	1.643	0.784	0.700	–
	Crystal-minimized	0.775	1.647	0.780	0.682	
Autodock Vina	Crystal-pose	0.600	–	0.60	0.53	Su et al. [64]
ΔSAS	Crystal-pose	0.62	–	0.63	0.59	
Δ_Vina_RF_20_	Crystal-pose	0.82	1.27	0.82	0.75	Wang et al. [33]

The crystal-reduced represent the dataset where protein–ligand pairs are obtained after removing the overlapped protein–ligand pair from the refined set. The crystal-minimized represent the dataset where ligands are locally optimized. The crystal-pose represents the experimental pose

*PCC* Pearson correlation coefficient, *RMSE* root-mean-square-error; *Sp* Spearman correlation coefficient

### Robustness and effectiveness of SMPLIP-Score

**Fig. 5:**
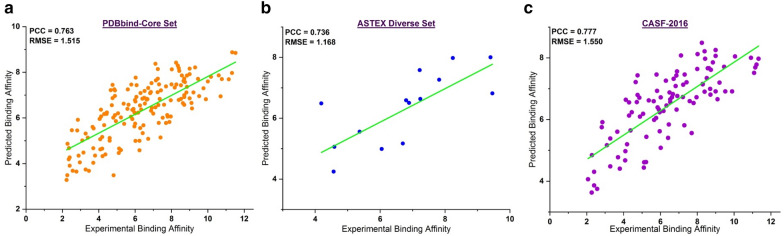
Thee Scatter plots of the predicted binding affinity against the experimental activity from the **a** PDBbind Core Set; **b** Astex Diverse Set; **c** CASF-2016 Set

Ligand-based or target-based discovery depends on the reliability of predicted binding poses either through experimental or computational methods. In particular, molecular docking simulations are the more popular method to generate such poses with a typical RMSD criterion (< 2.0 Å) than X-ray crystal poses. In addition, because every predictive model for binding affinity relies on features generated from input poses, the robustness of such a model is also affected by the input poses. Thus, we studied the robustness of SMPLIP-Score according to the change in input poses. To measure the robustness of SMPLIP-Score, we conducted docking simulations using selected datasets (PDBbind-Core Set, Astex Diverse Set, and CASF-2016) to generate docking poses. The molecular docking simulations were performed using the AutoDock Vina program [13] (Exhaustiveness: 32; Num_modes: 50), and after docking, only poses with RMSD < 2.0 Å from the crystal pose were selected as reliable poses. For the PDBbind Core Set, Astex Diverse Set, and CASF-2016 Set, a total of 163, 15, and 100 poses had RMSD < 2.0 Å, respectively. Then, SMPLIP features (IFP + Frag) were extracted from the molecular docking poses and the ligand-binding affinities were predicted. The prediction results are shown in Table 7 and the scatter plot is shown in Fig. 5.

**Table 7:** Prediction result based on simulated docking poses of the selected dataset

Dataset	PCC	RMSE	MAE	p_value	Sp
PDBbind Core Set	0.763	1.515	1.251	2.70E−32	0.749
Astex Diverse Set	0.736	1.168	0.878	0.003	0.846
CASF-2016	0.779	1.550	1.253	1.49E−21	0.785

*PCC* Pearson correlation coefficient, *RMSE* root-mean-square-error, *MAE* mean absolute error, *p_value* p_value for statistical significance, *Sp* Spearman correlation coefficient, *RF-Model* Random Forest parameters includes: n_estimators = 500; max_features = “AUTO”

When comparing the statistical performance of models built on docked poses (Table 7) with those built on crystal poses (Tables 1 and 5), we found that reasonable poses (RMSD < 2.0 Å) rarely made an effect on the affinity prediction and our result corroborated earlier work [31]. A slight variation in PCC and RMSE was observed between Tables 7 and 1 (or Table 5). In addition, feature matrices encoded by IChem and SMF can be directly compared between docked poses and crystal poses to reveal the interpretability of SMPLIP featurization. Thus, to analyze such changes, we randomly selected a few poses (crystal and docked) for comparison of interactions from these datasets. When two feature matrices were identical for both crystal and docked poses, they were excluded from this discussion. From the PDBbind Core Set, the superimposed crystal and docked poses for PDBs 2JDM and 3VH9 and IFP are shown in Additional file 1: Figure S17(a, b) and Tables S5, S6, respectively. The first selected 2JDM represents the protein–ligand pair from *Pseudomonas aeruginosa* lectin II (PA-IIL) in complex with methyl-a-l-fucopyranoside with the reported experimental binding affinity, pK_d_ = 5.4 M [65]. Both the crystal pose and docked pose with RMSD of 0.376 Å showed similar interactions (Additional file 1: Table S5) and thus had the same predicted affinity of 5.739 M. Similarly, another selected PDB, 3VH9, belongs to an *Aeromonas proteolytica* aminopeptidase enzyme bound with 8-quinolinol, with an experimentally determined binding affinity of 6.2 M [66]. The IFP in Additional file 1: Table S6 shows that most of the interactions from crystal and docked poses were common; however, additional interactions such as H-bonds (Asp117, Asp179) and hydrophobic interactions (Cys223) have been observed, resulting in an improvement in the predicted binding affinity from 4.362 M (crystal pose) to 4.579 M (docked pose). Furthermore, the binding interactions of the superimposed crystal and docked poses for the selected PDBs 1TT1 and 1SQN from the Astex Diverse Set are shown in Additional file 1: Figure S18(a, b) and those from IFP are shown in Additional file 1: Tables S7, S8, respectively. PDB 1TT1 is a GluR6 kainate receptor subunit bound to 3-(carboxymethyl)-4-isopropenylproline with 4.19 M experimental binding affinity [67]. The binding interaction revealed that its docked pose had almost the same orientations of the groups. Interestingly, the crystal and docked poses had the same interactions with the binding site residues, even with an RMSD of 1.176 Å, and both poses were predicted at 6.488 M. Similarly, another selected PDB was 1SQN, which represents the progesterone receptor in complex with norethindrone with a reported affinity of 9.4 M [68]. Notably, most of the interactions were hydrophobic, and the crystal and docked poses had almost identical IFPs except for additional hydrophobic (Leu763, Phe778) interactions for the docked pose. While the docked pose had additional interactions, similar predicted values of 8.086 M and 8.002 M were observed for the crystal and docked poses, respectively. Lastly, we selected PDBs 2Y5H and 1W4O from the CASF-2016 Set to study the IFP. The first selected PDB, 2Y5H, contains factor Xa, a serine protease from the blood coagulation cascade, crystallized with derivatives of pyrrolo[3,4-a]pyrrolizine [69]. The superimposed crystal and docked poses and IFP are shown in Additional file 1: Figure S19(a) and Table S9, respectively. The calculated IFP showed identical interactions for the crystal and docked poses with the same predicted values of 7.459 M. Another selected PDB, 1W4O, represents ribonuclease-A protein in bound form with non-natural 3′-nucleotides [70] (Additional file 1: Figure S19(b)). The calculated IFP (Additional file 1: Table S10) for the docked pose shows that most of the interactions are shared by the crystal pose. However, a few interactions change their types while interacting with binding site residues. For example, the Phe120 residue formed H-bonds with the ligand (HBond_LIG) in the docked pose, whereas in the crystal pose, the backbone of the same residue formed HBond_PROT with the ligand. Similarly, in the crystal pose, Lys41 formed ionic (Ionic_PROT) interactions, while in the docked pose it formed H-bond (HBond_PROT) interactions. These trivial changes were reflected in the predicted affinity of the crystal pose (4.460) and docked pose (4.621).

Despite there being a close relationship between the predicted values for crystal and docked poses, we further evaluated the prediction values for poses with high RMSD values and checked the effectiveness of the SMPLIP-Score. For this purpose, we selected some poses with high RMSDs and predicted the binding affinity values (Additional file 1: Figures S20–S21 and Tables S11–S12). Additional file 1: Figure S20, Tables S11, S13 show the comparison of the binding pose (docked) and IFP for PDB 2JDM. This revealed that the docked pose with an RMSD of 3.53 Å (Additional file 1: Figure S20(b)) retained most of the hydrophobic interactions (Asp99, Ser23, and Thr98), but lost most hydrogen bond interactions, when compared with the crystal pose; this pose was predicted at pKd = 4.869 M. Likewise, another docked pose with an RMSD of 3.043 Å (Additional file 1: Figure S20(c)) also lost most of the hydrophobic and hydrogen bond interactions and predicted an affinity of 4.846 M. Notably, both predicted values for the docked poses were lower than the experimental binding affinity (5.4 M), while the most preferable docked pose with an RMSD of 0.376 Å (Additional file 1: Figure S20(a)) predicted 5.703 M, closer to the experimental value. This suggests that a reliable pose was required for an accurate prediction. Moreover, Additional file 1: Figure S21 and Table S12 show the comparison of the binding pose and IFP for docked poses from PDB 3VH9. Notably, the SMPLIP-Score predicted the crystal pose at 4.362 M, while its docked pose predicted 4.579 M (RMSD: 0.531 Å), 3.963 M (RMSD: 2.621 Å), and 4.199 M (RMSD: 4.27 Å) (Additional file 1: Table S13). Remarkably, the docked pose that predicted a value of 3.963 M showed only hydrophobic (Met180 and Ile255) interactions, but it did not make any hydrogen bond interactions due to flipping of the pose in the binding pocket. The prediction result further rationalizes the reliable pose requirement for accurate binding affinity predictions.

Overall, the comparison of IFP results from both docked and crystal poses indicates that, during the virtual screening (molecular docking) experiments, the identified small molecules must have complementary interactions with the crystal pose to be predicted accurately. Nonetheless, the additional interactions observed in the protein–ligand complexes may lead to changes in the observed prediction of binding affinity.

## Conclusions

Herein, we report SMPLIP-Score as a robust and effective predictor and compared it with state-of-the-art featurization processes/methods. SMPLIP features, originating from protein–ligand interaction patterns and ligand features, showed cost-effectiveness as well as interpretability of the feature matrix embedded for learning. Most notably, the best SMPLIP features (IFP + Frag) demonstrated scoring power, ranking power, and robustness using various benchmark datasets. Obviously, the comparison between crystal and docked poses verified the robustness of SMPLIP-Score against input poses. Their interpretable feature matrices can be used directly to provide insight into ligand binding to a protein and the integrated description of binding mode with predicted affinity (having high accuracy) is a replaceable predictor of current scoring function.

## Supplementary Information

**Additional file 1**. Additional tables, figures, and methods are available.

## Data Availability

Knime workflow, python code, and refined data will be available in GitHub. https://github.com/college-of-pharmacy-gachon-university/SMPLIP-Score.
