# Supplementary material for: SMPLIP-Score: predicting ligand binding affinity from simple and interpretable on-the-fly interaction fingerprint pattern descriptors
Source: J Cheminform. 2021 Mar 25;13:28. doi: 10.1186/s13321-021-00507-1 (PMC7993508; doi:10.1186/s13321-021-00507-1)
Supplement: Supplementary file 1 — Additional file 1. Additional tables, figures, and methods are available. [file 13321_2021_507_MOESM1_ESM.docx]

**Additional file 1**

**Contents:**

| **S.**  **No.** | **Description** | **Page**  **No.** |
| --- | --- | --- |
| **1.** | **Figure S1:** KNIME workflow for processing of protein file. | **4** |
| **2.** | **Figure S2:** PDBbind (Release 2015) dataset characterization. | **5** |
| **3.** | Feature constructions | **6-8** |
| **4.** | **Table S1:** The Result of statistical performance using the Random Forest model. | **9-10** |
| **5.** | **Table S2:** The statistical comparison of different feature type and size. | **11** |
| **6.** | SMPLIP Random Forest and DNN Model | **12-13** |
| **7.** | **Figure S3-S10:** Statistical comparison of a) PCC (Person-Correlation-Coefficient) and b) RMSE (Root-Mean-Square-Error) between different SETS for Train, Valid and Test data based on IFP, IFP+Int-Dist, IFP+Frag, IFP+Int-Dist+Frag as features for RF model. | **14-21** |
| **8.** | **Table S3:** The Result of statistical performance using the DNN model. | **22** |
| **9.** | **Figure S11-S14:** Statistical comparison of a) PCC (Person-Correlation-Coefficient) and b) RMSE (Root-Mean-Square-Error) between different SETS for Train, Valid and Test data based on IFP, IFP+Dist, IFP+Frag, IFP+Dist+Frag as features for DNN model. | **23-26** |
| **10.** | **Figure S15:** Statistical performance of PCC (Person-Correlation-Coefficient) and RMSE (Root-Mean-Square-Error) for Train and Valid data as a function of Epochs based on IFP+Frag as features. | **27** |
| **11.** | **Table S4:** The variance analysis of the Random Forest (RF) and Deep Neural Network (DNN) models. | **28** |
| **12.** | **Figure S16:** The binding affinity predictions for the Five benchmark dataset with IFP+Frag features using Random Forest Model. | **29-30** |
| **13.** | **Figure S17-S19:** The superimposition of docked pose (magenta color) over the crystal pose (green color) for PDBs from PDBbind Core Set, Astex Diverse Set and CASF-2016 Set. | **31-36** |
| **14.** | **Table S5-S10:** Interaction Fingerprint Pattern (IFP) calculated for Crystal pose and Docked pose for PDBs from PDBbind Core Set, Astex Diverse Set and CASF-2016 Set. | **31-36** |
| **15.** | **Figure S20-S21**: Comparison of crystal pose with docked pose (different RMSD) from PDBbind Core Set. | **37-38** |
| **16.** | **Table S11-S12:** Interaction Fingerprint Pattern (IFP) calculated for Crystal pose and two Docked pose for PDBs. | **37-38** |
| **17.** | **Table S13:** The Experimental and Predicted Values for Crystal and Docked pose from selected PDBs. | **39** |
| **18** | **Table S14:** The Performance Comparison Between SMPLIP Feature and PLEC Feature. | **39** |

**Figure S1:** KNIME workflow for processing of protein file from PDBbind (Release 2015) dataset.


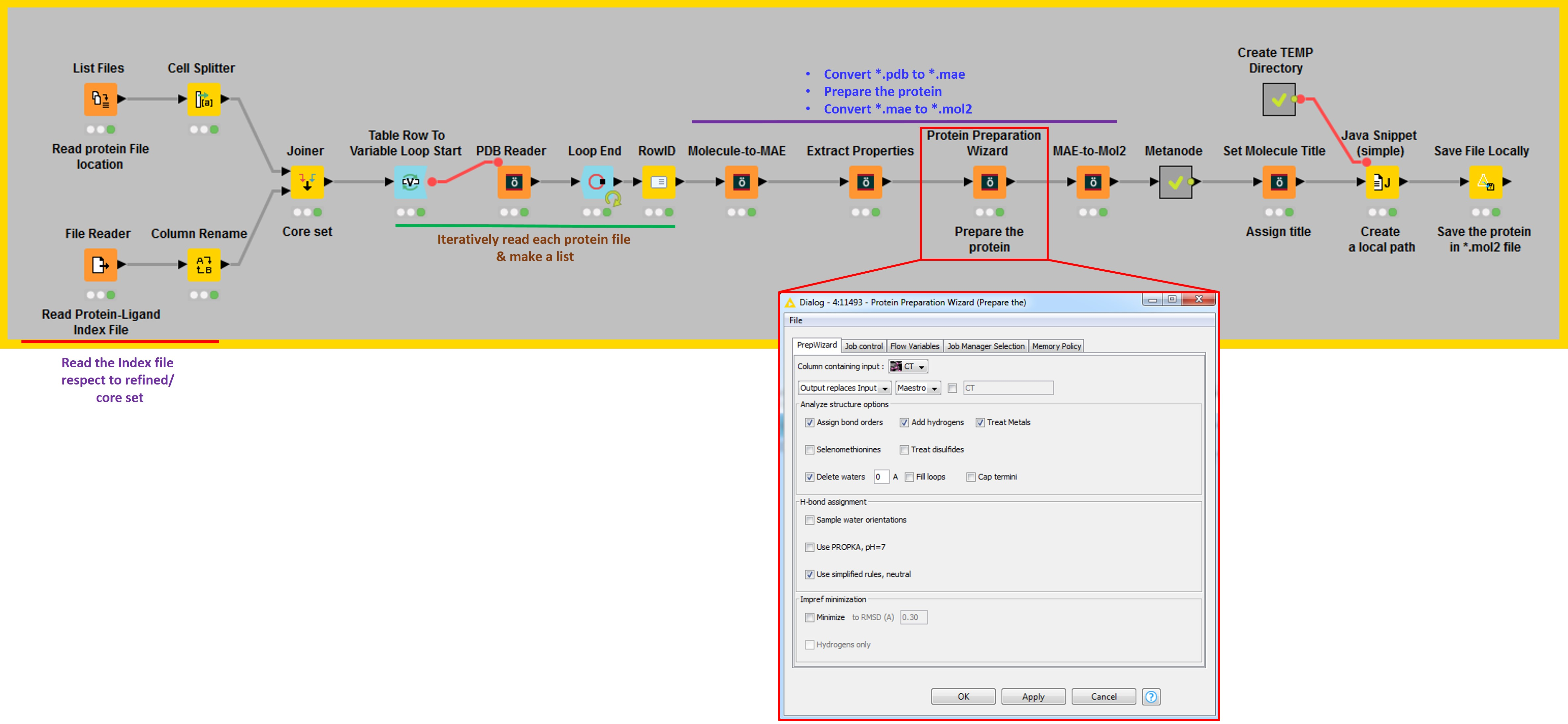


The workflow read the protein in *.pdb format and also the respective index file (which stores protein-ligand binding strength data, resolution, binding strength unit). After reading the input files, a loop was introduced, which iteratively read all the *.pdb file and store in each row. After the loop node, the nodes from Schrodinger was added, which convert the *pdb into *.mae format and then prepare the protein. The protein preparation steps include the addition of H-atoms, assign bond order, treat mental, remove all water molecules, and assign simplified rules for the protonation state of each amino acid. Once the preparation completes, the *.mae format converted into *.mol2 file and each protein was assigned with PDB-ID. To save each protein files into individual *.mol2 file format, a temporary directory was created and all the protein files saved in the local directory of the system.

**Figure S2:** PDBbind (Release 2015) dataset characterization. Resolution range for refined set (n = 3481) **(a)** and core set (n = 180) **(b)**; Experimentally measured binding strength distribution in Refined set **(c)** and Core set **(d)**; Ligand length (Å) distribution for refined and core set **(e)**.


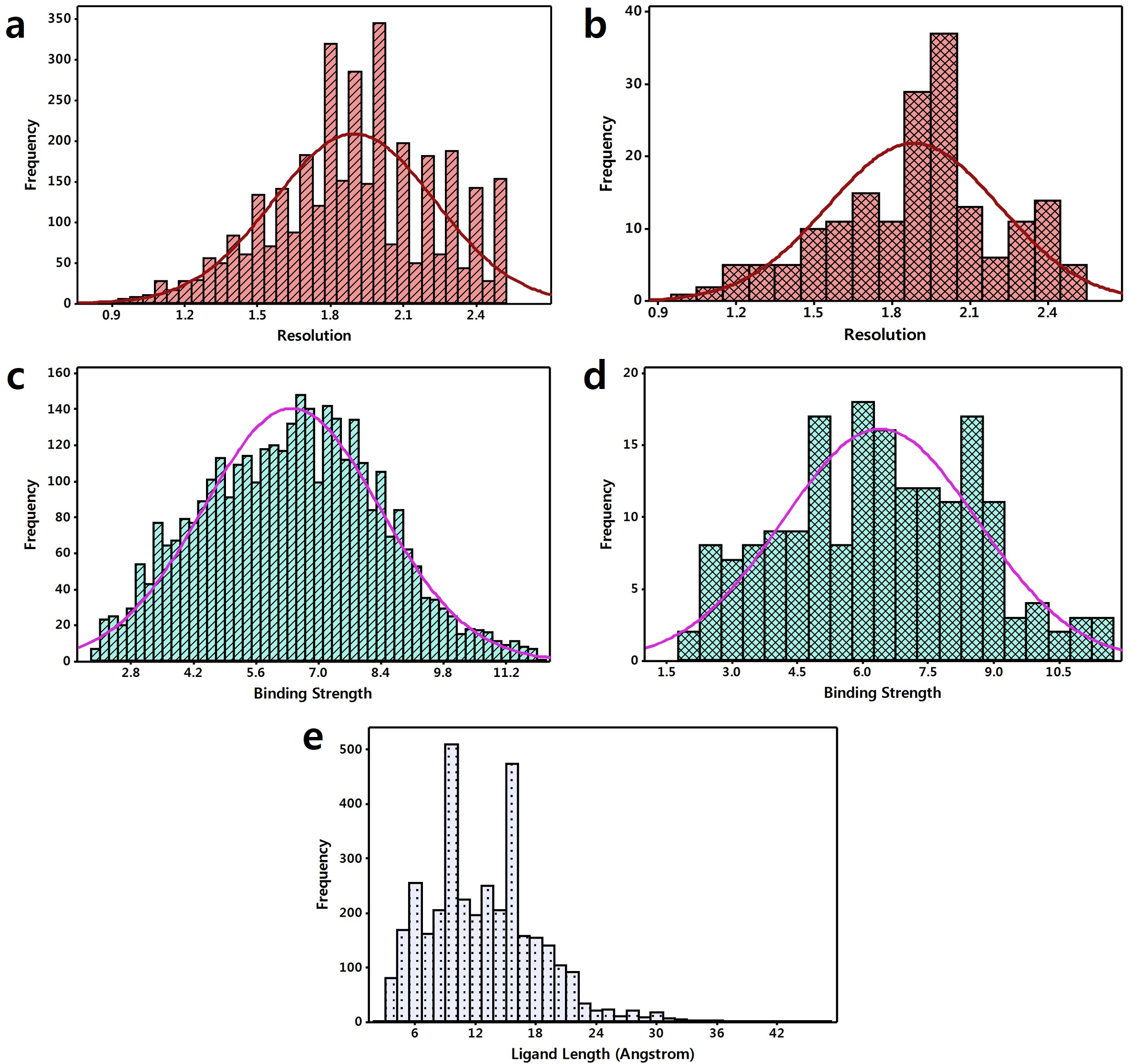


**Feature Constructions:**

**Interaction Fingerprint Pattern (IFP):**


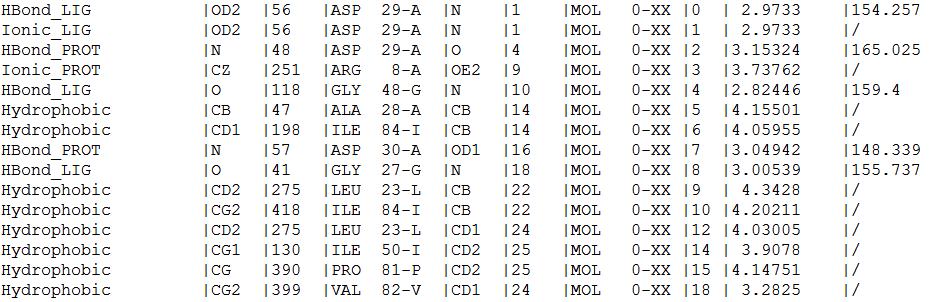


A typical interaction fingerprint is shown above, where each columns are defined as follows:

1^st^  column: type of interaction

2^nd^  column: interacting protein atom name

3^rd^  column:  interacting protein atom number

4^th^  column:  interacting protein residue name & number

5^th^  column: interacting ligand atom name

6^th^  column:  interacting ligand atom number

7^th^  column:  interacting ligand residue name & number

8^th^  column: interaction distance

9^th^  column: interaction angle (for H‐bonds only)

If similar interaction type (i.e. either hydrophobic or HBond_LIG, etc) reported by the same type of amino acid, then it was summed up. Similarly, if the same amino acids were reporting the distance, then its values were summed up, to maintain the dimension of the matrix, which latter represent the IFP and Dist matrix respectively. During the matrix creation, each information (i.e. interacting protein residue and type; interaction distances) were extracted using the KNIME workflow (IChem_IFP_PDBBind_2015.knwf (https://github.com/college-of-pharmacy-gachon-university/SMPLIP-Score)), and saved into *.csv format.

**Substructural Fragment Descriptors for Ligands:** The SMF program generates the ligand fragments in hashed form. The detailed information about the SMF and its fragmentation pattern can be seen as follows; The SMF program, define the two different classes of substructure molecular fragments: 1) “sequences” (I) and 2) “augmented atoms” (II). Three sub-types are also defined for each class as atoms and bonds (AB), atoms only (A) or bonds only (B).

For the sequence (I), only the shortest paths from one atom to the others are considered. For each type of sequence, which constitutes, the atoms and bonds (AB), atoms only (A) or bonds only (B), the minimum (n_min_) and maximum (n_max_) number of constituent atoms are defined. Thus, based on the partitioning I(AB,n_min_→n_max_), I(A, n_min_→n_max_) and I(B, n_min_→n_max_), the SMF program generates ‘‘intermediate’’ sequences involving n atoms (n_min_≤n≤n_max_) (**Figure 1**).


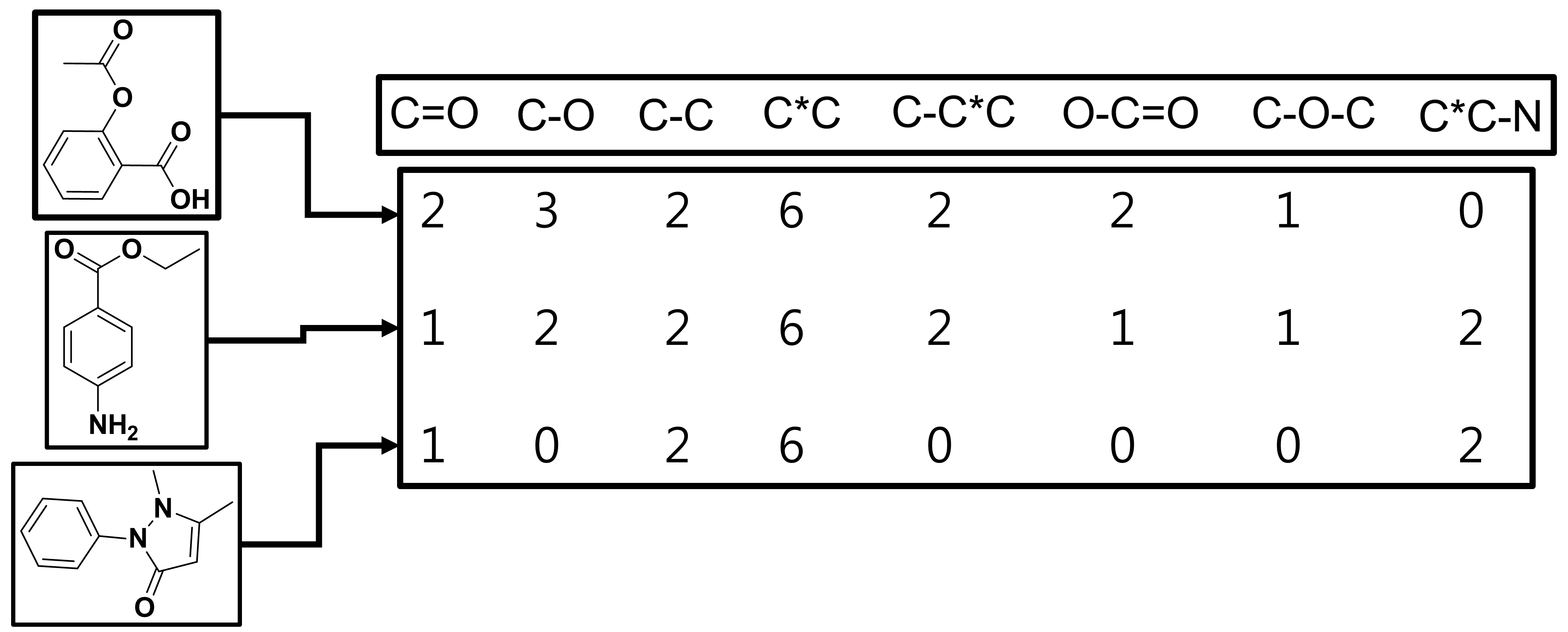


**Figure 1:** Example showing a part of descriptor matrix for three organic compounds. The integer numbers correspond to occurrences of the constituent substructural fragments.

Similarly, an ‘‘augmented atom’’ represents a selected atom with its environment including either neighboring atoms or bonds (AB), or atoms only (A), or bonds only (B). Atomic hybridization (Hy) can be taken into account for augmented atoms of the A-type (**Figure 2**).


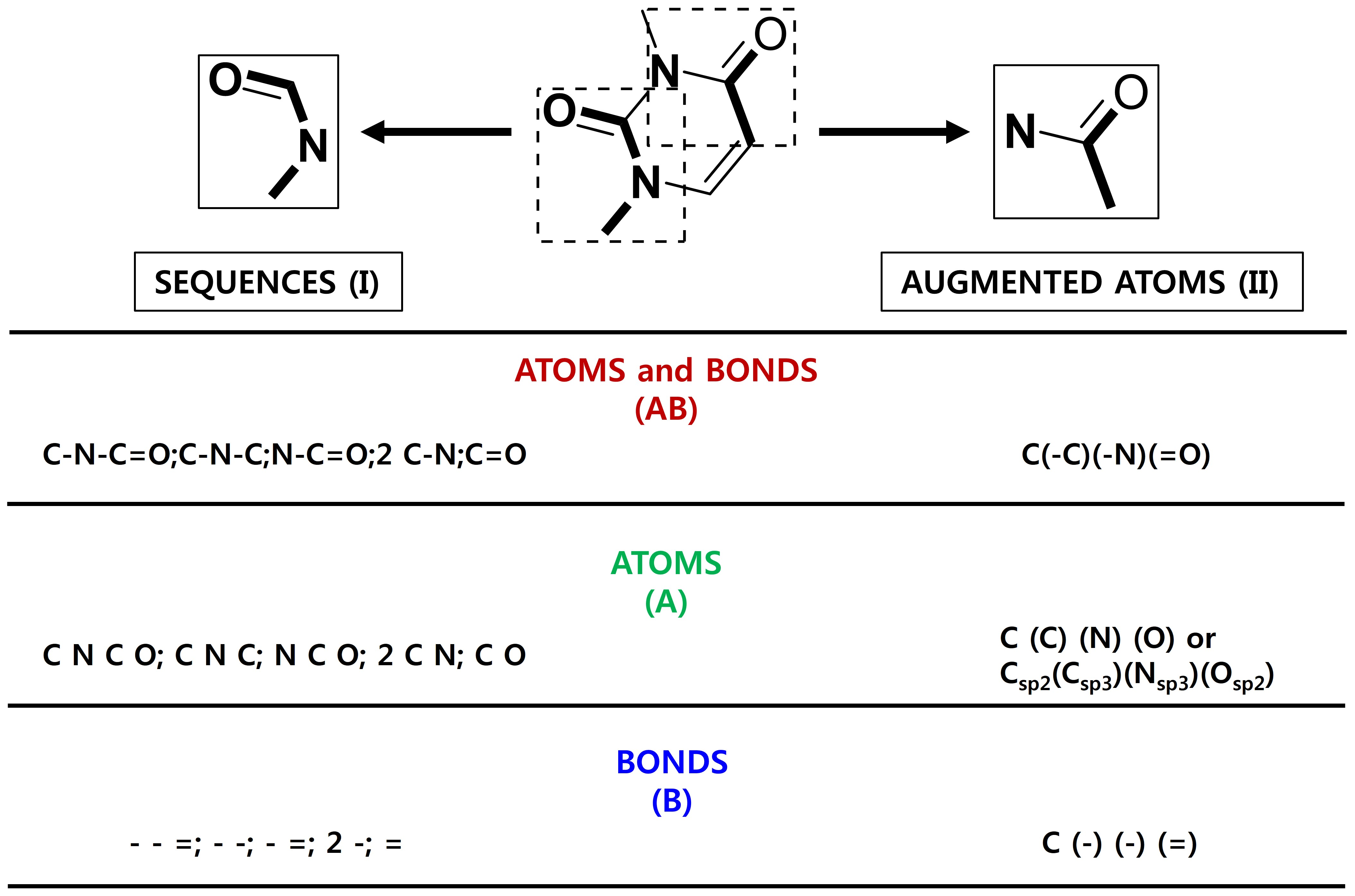


**Figure 2:** Two classes of substructural fragments: atom/bond sequences and augmented atoms. Shortest paths sequences (I) and augmented atoms (II) including atoms and bonds (AB), only atoms (A) or only bonds (B).

**References:** Substructural fragments: an universal language to encode reactions, molecular and supramolecular structures, A. Varnek, D. Fourches, F. Hoonakker,V. P. Solov’ev, Journal of Computer-Aided Molecular Design (2005) 19: 693–703.

**Table S1:** The Result of statistical performance on PDBbind (Release 2015) using the Random Forest model. The Refined set (n =3481) used for training and validation, and core set (n=180) as a test set. The valid represent 20% of the refined set. The boldface represents the model with good statistics in each category.

| **Features** | **Random Forest parameters** | | **SETS** | **Train** | | | | **Valid** | | | | **Test** | | | |
| --- | --- | --- | --- | --- | --- | --- | --- | --- | --- | --- | --- | --- | --- | --- | --- |
|  | **max_**  **features** | **n_**  **estimators** |  | **RMSE** | **MAE** | **PCC** | **p-value** | **RMSE** | **MAE** | **PCC** | **p-value** | **RMSE** | **MAE** | **PCC** | **p_value** |
| **IFP** | auto | 500 | SET01 | 0.534 | 0.416 | 0.975 | 0 | 1.467 | 1.153 | 0.677 | 1.26E-94 | 1.700 | 1.376 | 0.667 | 1.62E-24 |
|  |  | 200 | SET02 | 0.540 | 0.415 | 0.974 | 0 | 1.424 | 1.113 | 0.677 | 1.80E-94 | 1.734 | 1.394 | 0.648 | 7.68E-23 |
|  |  | 300 | SET03 | 0.531 | 0.414 | 0.975 | 0 | 1.474 | 1.122 | 0.665 | 5.26E-90 | 1.758 | 1.406 | 0.629 | 3.17E-21 |
|  |  | 100 | SET04 | 0.545 | 0.424 | 0.973 | 0 | 1.423 | 1.098 | 0.696 | 3.88E-102 | 1.696 | 1.374 | 0.670 | 8.18E-25 |
|  |  | **100** | **SET05** | **0.543** | **0.422** | **0.974** | **0** | **1.401** | **1.062** | **0.707** | **1.70E-106** | **1.667** | **1.343** | **0.686** | **2.20E-26** |
|  |  | 200 | SET06 | 0.532 | 0.415 | 0.975 | 0 | 1.454 | 1.136 | 0.705 | 9.45E-106 | 1.686 | 1.361 | 0.676 | 2.26E-25 |
|  | sqrt | 100 | SET01 | 0.534 | 0.414 | 0.977 | 0 | 1.459 | 1.151 | 0.686 | 6.89E-98 | 1.683 | 1.362 | 0.707 | 1.50E-28 |
|  |  | 100 | SET02 | 0.535 | 0.416 | 0.978 | 0 | 1.381 | 1.089 | 0.706 | 2.39E-106 | 1.729 | 1.400 | 0.676 | 2.04E-25 |
|  |  | 300 | SET03 | 0.521 | 0.411 | 0.979 | 0 | 1.448 | 1.121 | 0.682 | 1.48E-96 | 1.741 | 1.409 | 0.666 | 1.81E-24 |
|  |  | 200 | SET04 | 0.529 | 0.414 | 0.979 | 0 | 1.389 | 1.090 | 0.718 | 2.44E-111 | 1.700 | 1.372 | 0.697 | 1.68E-27 |
|  |  | **100** | **SET05** | **0.537** | **0.420** | **0.977** | **0** | **1.372** | **1.066** | **0.726** | **4.17E-115** | **1.656** | **1.349** | **0.716** | **1.49E-29** |
|  |  | 300 | SET06 | 0.523 | 0.411 | 0.979 | 0 | 1.447 | 1.143 | 0.717 | 7.76E-111 | 1.696 | 1.364 | 0.698 | 1.37E-27 |
| **IFP +**  **Int_Dist** | auto | 100 | SET01 | 0.558 | 0.434 | 0.973 | 0 | 1.528 | 1.216 | 0.642 | 3.50E-82 | 1.738 | 1.426 | 0.659 | 9.40E-24 |
|  |  | 100 | SET02 | 0.567 | 0.440 | 0.974 | 0 | 1.458 | 1.161 | 0.659 | 6.31E-88 | 1.769 | 1.453 | 0.636 | 8.50E-22 |
|  |  | 500 | SET03 | 0.546 | 0.432 | 0.977 | 0 | 1.529 | 1.175 | 0.632 | 5.46E-79 | 1.783 | 1.453 | 0.632 | 1.71E-21 |
|  |  | 500 | SET04 | 0.550 | 0.435 | 0.977 | 0 | 1.456 | 1.148 | 0.681 | 3.91E-96 | 1.750 | 1.439 | 0.661 | 5.14E-24 |
|  |  | **200** | **SET05** | **0.549** | **0.432** | **0.976** | **0** | **1.447** | **1.131** | **0.682** | **1.21E-96** | **1.711** | **1.410** | **0.677** | **1.91E-25** |
|  |  | 500 | SET06 | 0.541 | 0.427 | 0.977 | 0 | 1.503 | 1.190 | 0.682 | 1.55E-96 | 1.765 | 1.439 | 0.642 | 2.54E-22 |
|  | sqrt | 400 | SET01 | 0.530 | 0.417 | 0.980 | 0 | 1.478 | 1.173 | 0.678 | 7.55E-95 | 1.712 | 1.407 | 0.696 | 2.37E-27 |
|  |  | 100 | SET02 | 0.548 | 0.428 | 0.978 | 0 | 1.398 | 1.104 | 0.699 | 1.72E-103 | 1.749 | 1.425 | 0.675 | 2.52E-25 |
|  |  | 400 | SET03 | 0.529 | 0.418 | 0.980 | 0 | 1.472 | 1.146 | 0.669 | 1.07E-91 | 1.766 | 1.444 | 0.665 | 2.58E-24 |
|  |  | 400 | SET04 | 0.533 | 0.420 | 0.980 | 0 | 1.421 | 1.126 | 0.704 | 2.67E-105 | 1.715 | 1.406 | 0.700 | 7.69E-28 |
|  |  | **300** | **SET05** | **0.536** | **0.422** | **0.980** | **0** | **1.387** | **1.093** | **0.720** | **2.08E-112** | **1.692** | **1.388** | **0.711** | **5.15E-29** |
|  |  | 200 | SET06 | 0.531 | 0.417 | 0.980 | 0 | 1.481 | 1.180 | 0.700 | 1.12E-103 | 1.732 | 1.402 | 0.684 | 3.92E-26 |
| **IFP + Frag** | auto | **100** | **SET01** | **0.496** | **0.381** | **0.977** | **0** | **1.327** | **1.035** | **0.747** | **2.62E-125** | **1.489** | **1.227** | **0.771** | **8.71E-37** |
|  |  | 100 | SET02 | 0.503 | 0.383 | 0.976 | 0 | 1.329 | 1.023 | 0.727 | 2.36E-115 | 1.555 | 1.270 | 0.739 | 2.55E-32 |
|  |  | 200 | SET03 | 0.493 | 0.380 | 0.978 | 0 | 1.296 | 0.991 | 0.755 | 1.78E-129 | 1.552 | 1.266 | 0.747 | 2.02E-33 |
|  |  | 100 | SET04 | 0.502 | 0.382 | 0.976 | 0 | 1.296 | 1.009 | 0.758 | 4.45E-131 | 1.547 | 1.257 | 0.743 | 6.76E-33 |
|  |  | 300 | SET05 | 0.494 | 0.383 | 0.978 | 0 | 1.310 | 0.992 | 0.751 | 2.87E-127 | 1.547 | 1.274 | 0.748 | 1.83E-33 |
|  |  | 200 | SET06 | 0.487 | 0.374 | 0.978 | 0 | 1.330 | 1.049 | 0.764 | 4.02E-134 | 1.568 | 1.286 | 0.732 | 1.63E-31 |
|  | sqrt | **100** | **SET01** | **0.503** | **0.389** | **0.977** | **0** | **1.362** | **1.065** | **0.731** | **1.54E-117** | **1.519** | **1.252** | **0.761** | **2.36E-35** |
|  |  | 400 | SET02 | 0.497 | 0.384 | 0.979 | 0 | 1.331 | 1.027 | 0.727 | 9.85E-116 | 1.589 | 1.314 | 0.731 | 2.61E-31 |
|  |  | 500 | SET03 | 0.500 | 0.388 | 0.979 | 0 | 1.317 | 1.014 | 0.747 | 3.43E-125 | 1.594 | 1.317 | 0.730 | 2.80E-31 |
|  |  | 400 | SET04 | 0.499 | 0.386 | 0.979 | 0 | 1.327 | 1.051 | 0.745 | 3.19E-124 | 1.580 | 1.292 | 0.733 | 1.52E-31 |
|  |  | 100 | SET05 | 0.512 | 0.393 | 0.976 | 0 | 1.340 | 1.027 | 0.739 | 3.64E-121 | 1.561 | 1.287 | 0.751 | 6.54E-34 |
|  |  | 100 | SET06 | 0.506 | 0.387 | 0.977 | 0 | 1.349 | 1.071 | 0.758 | 7.74E-131 | 1.598 | 1.300 | 0.730 | 2.96E-31 |
| **IFP +**  **Int_Dist +**  **Frag** | auto | **100** | **SET01** | **0.498** | **0.381** | **0.976** | **0** | **1.332** | **1.037** | **0.745** | **3.06E-124** | **1.503** | **1.235** | **0.766** | **5.57E-36** |
|  |  | 200 | SET02 | 0.491 | 0.377 | 0.978 | 0 | 1.318 | 1.016 | 0.732 | 4.56E-118 | 1.553 | 1.287 | 0.740 | 1.55E-32 |
|  |  | 100 | SET03 | 0.498 | 0.382 | 0.977 | 0 | 1.310 | 0.995 | 0.749 | 3.21E-126 | 1.556 | 1.274 | 0.744 | 5.17E-33 |
|  |  | 100 | SET04 | 0.499 | 0.382 | 0.977 | 0 | 1.308 | 1.024 | 0.753 | 2.20E-128 | 1.551 | 1.267 | 0.741 | 1.14E-32 |
|  |  | 100 | SET05 | 0.494 | 0.382 | 0.977 | 0 | 1.316 | 1.004 | 0.748 | 9.09E-126 | 1.549 | 1.272 | 0.746 | 2.83E-33 |
|  |  | 100 | SET06 | 0.496 | 0.377 | 0.977 | 0 | 1.336 | 1.053 | 0.760 | 2.46E-132 | 1.564 | 1.287 | 0.734 | 1.13E-31 |
|  | sqrt | **100** | **SET01** | **0.494** | **0.382** | **0.978** | **0** | **1.346** | **1.054** | **0.740** | **8.07E-122** | **1.512** | **1.244** | **0.770** | **1.43E-36** |
|  |  | 400 | SET02 | 0.494 | 0.382 | 0.980 | 0 | 1.314 | 1.013 | 0.736 | 4.20E-120 | 1.587 | 1.313 | 0.738 | 3.32E-32 |
|  |  | 100 | SET03 | 0.506 | 0.390 | 0.978 | 0 | 1.307 | 1.004 | 0.752 | 3.54E-128 | 1.564 | 1.290 | 0.751 | 5.70E-34 |
|  |  | 100 | SET04 | 0.505 | 0.389 | 0.978 | 0 | 1.314 | 1.040 | 0.751 | 1.29E-127 | 1.574 | 1.289 | 0.744 | 5.65E-33 |
|  |  | 300 | SET05 | 0.499 | 0.387 | 0.979 | 0 | 1.319 | 1.018 | 0.751 | 3.74E-127 | 1.561 | 1.290 | 0.758 | 7.87E-35 |
|  |  | 300 | SET06 | 0.490 | 0.379 | 0.980 | 0 | 1.343 | 1.069 | 0.762 | 2.38E-133 | 1.587 | 1.307 | 0.739 | 2.57E-32 |

**Note:** Random_State values assigned for different Sets are as SET01: 123456; SET02: 42; SET03: 56789; SET04: 98765; SET05: 4321; SET06: 1234 to reproduce the same statistical result for training and validation data. **RMSE:** Root-Mean-Square-Error; **MAE:** Mean Absolute Error; **PCC:** Pearson Correlation Coefficient; **p_value:** p_value for statistical significance.

**Description:** A total of six different sets were created by assigning different random seed. Each set of model was trained with different options of max_feature (‘auto’, or ‘sqrt’) and n_estimators (100, 200, 300, 400, 500). From all the models for each set, best model from each ‘auto’ and ‘sqrt’ options was selected based on lowest RMSE for test set.

**Table S2:** The statistical comparison of different feature type and size.

| **Features** | **Train Set** | | | **Valid Set** | | | **Test Set** | | |
| --- | --- | --- | --- | --- | --- | --- | --- | --- | --- |
|  | **PCC** | **RMSE** | **MAE** | **PCC** | **RMSE** | **MAE** | **PCC** | **RMSE** | **MAE** |
| **Null Feature (2422)** | 0.945 | 0.678 | 0.497 | 0.708 | 1.407 | 1.101 | 0.718 | 1.572 | 1.295 |
| **IFP Feature (140)** | 0.977 | 0.537 | 0.420 | 0.724 | 1.372 | 1.066 | 0.716 | 1.656 | 1.349 |
| **IFP + Frag (2422)** | 0.977 | 0.496 | 0.381 | 0.747 | 1.327 | 1.035 | 0.771 | 1.489 | 1.227 |

**Note:** The Null Feature model represent the input matrix, where all IFP values were set to ‘zero’, while, all Frag feature values were retained (IFP (all zero) + Frag) and the total size of input matrix was 2422. IFP and IFP+Frag Feature model is the same, which was presented in Table 1 of the Manuscript and size of input matrix was 140 and 2422 feature respectively.

**Description:** From the above table, the calculated metrics for Null feature model and its comparison with other model revealed that, Null feature model was statistically at par with IFP feature based model, while a little lower RMSE_Test_ (1.572) was observed. Such performance was expected as selection of correct featurization for ligand, not only improves the model’s performance, sometimes, it outperform any target based model. Notably, both model consider single feature, one is derived from ligand and another from protein-ligand interaction. Further, the model building from combined feature of IFP+Frag, not only improves the PCC, but it also reduces the prediction error in terms of RMSE_Test_ (1.489) and MAE_Test_ (1.227). Thus, it can said that, the addition of Interaction Fingerprint (IFP) information further improves the statistical metrics, and such observarions were also reported by Boyles et al. (Boyles, et al., Learning from the ligand: using ligand-based features to improve binding affinity prediction Bioinformatics, 36(3), 2020, 758–764; doi: 10.1093/bioinformatics/btz665).

**SMPLIP Random Forest and DNN Model:**

**SMPLIP-RF Model:** The RF model with the IFP feature alone has achieved PCC for the training data to 0.977 with an RMSE of 0.537. On the validation data, this model has a prediction power of 0.724 (PCC) with an RMSE of 1.372. Since validation data was from refined data, so we further evaluated its prediction power on the core data as a test set. Contrary to the validation data result, the IFP feature-based model for test set has achieved a PCC of 0.716 and an RMSE of 1.656 with a significance level (p_value) of 1.49E-29. Compared to the PCC of reported models for core set (n = 180) as a test set: Vina (0.676), RF-Score (0.710), RF-Score-v3 (0.746) and NNScore 2.0 (0.751)^1^, our model (0.716) showed obviously better performance than Vina, RF-Score (**Table1**). Furthermore, it is rationalized that the shape and size of bound ligands can be useful information with the improved predictive power (particularly, test data MAE: 1.227, RMSE: 1.489) through the significance of the ligand fragments that belong to neighbor atoms and augmented atoms as features. The model (IFP+Frag: 0.771) has higher PCC and comparable performance than Boyles’s report (Vina + RDKit: 0.749; RF-Score + RDKit: 0.778), though being slightly lower than models from (RF-Score-v3 + RDKit: 0.780; NNScore 2.0 + RDKit: 0.786)^1^. On observation of improved statistics for IFP+Frag features, we further combined all three features (IFP+Int-Dist+Frag) to measure the prediction performance. This model has achieved at par statistics as compared to IFP+Frag features in terms of PCC for training, validation, and test data, however the RMSE for the test data increased to 1.512 as compared with RMSE of IFP+Frag (1.489).

**Reference:**

1. Boyles, et al., Learning from the ligand: using ligand-based features to improve binding affinity prediction Bioinformatics, 36(3), 2020, 758–764; doi: 10.1093/bioinformatics/btz665

**SMPLIP-DNN-Model:** Likewise for SMPLIP-DNN model, the IFP feature showed PCC of 0.899, 0.678, and 0.726, while RMSE was found to be 0.874, 1.483 and 1.538 for the train, valid and test data respectively. Like the random forest model, although the performance of the IFP feature model on training and validation data was not at par, this model performed well for test data in terms of RMSE (1.538) and PCC (0.726), with LOSS statistics at 1.032. With improved statistics on test data for IFP features, we further combined the Interaction distance features with IFP. Although the combined model did not improve its statistics for PCC (0.713) and RMSE (1.582) on test data, this model did perform better for SMPLIP-RF (IFP+Int-Dist). Notably to mention here that, in the RF model, the IFP+Frag features have higher predictive power for test data, so we further built the DNN model with these features. Even if SMPLIP-DNN (IFP+Frag) did not present dramatic improvement, it has the highest prediction power for test data (PCC: 0.733; RMSE: 1.530) than other DNN models. This performance was stable against epochs as shown in **Figure S15** to illustrates the comparison of PCC and RMSE of the train and valid data against epochs. The best model was obtained for a batch size of 64; dropout of 0.1 and alpha value of 0.7 at 129 epochs.

**Figure S3:** Statistical comparison of **a)** PCC (Person-Correlation-Coefficient) and **b)** RMSE (Root-Mean-Square-Error) between different SETS for Train, Valid and Test data based on Interaction Fingerprint Pattern (IFP) as features (RF parameters: max_features = 'auto'). The statistics shown in red color are better among other SETS.


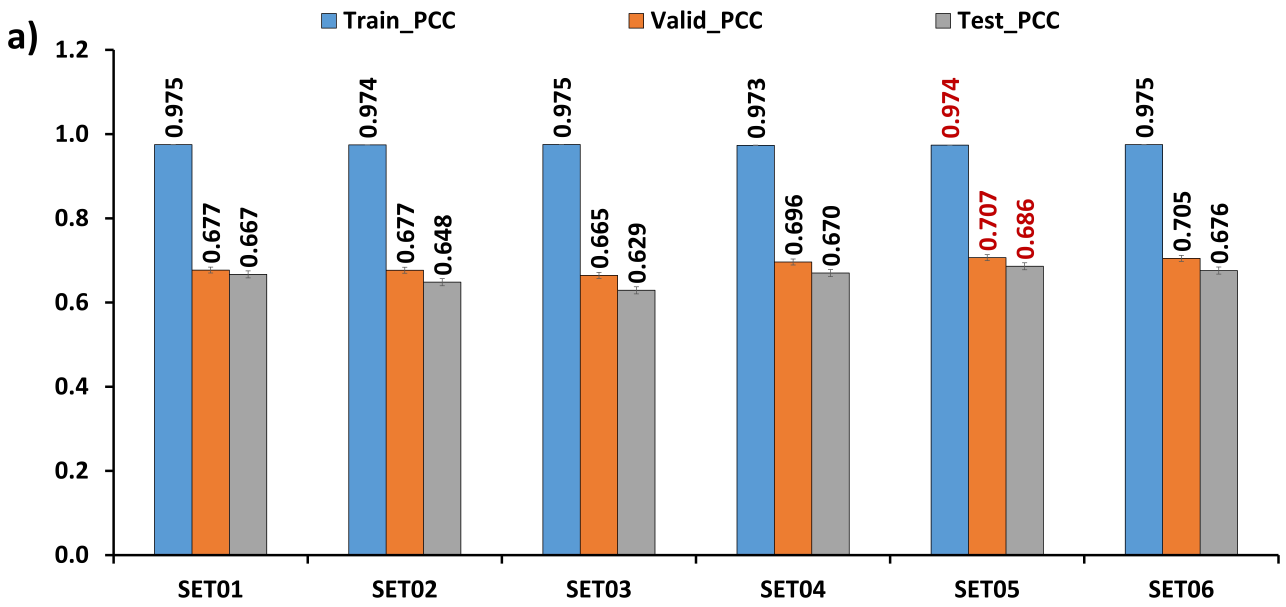


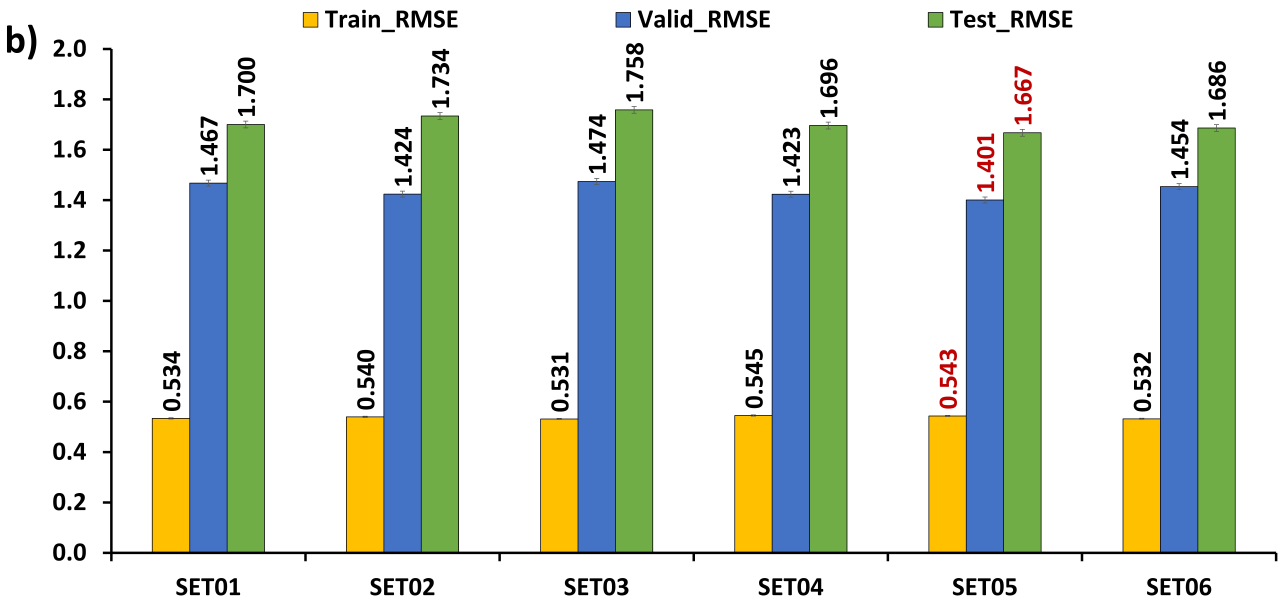


**Figure S4:** Statistical comparison of **a)** PCC (Person-Correlation-Coefficient) and **b)** RMSE (Root-Mean-Square-Error) between different SETS for Train, Valid and Test data based on Interaction Fingerprint Pattern (IFP) as features (RF parameters: max_features = 'sqrt'). The statistics shown in red color are better among other SETS.


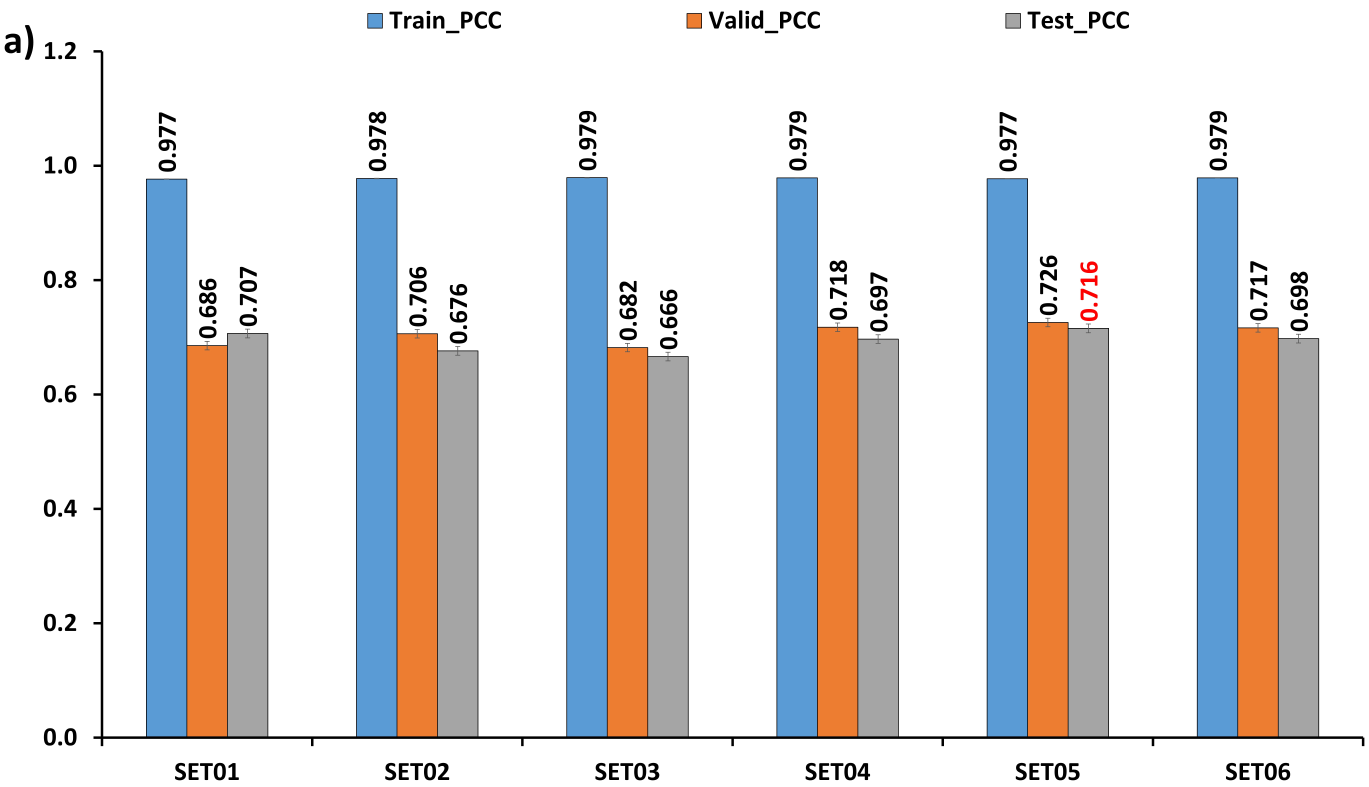


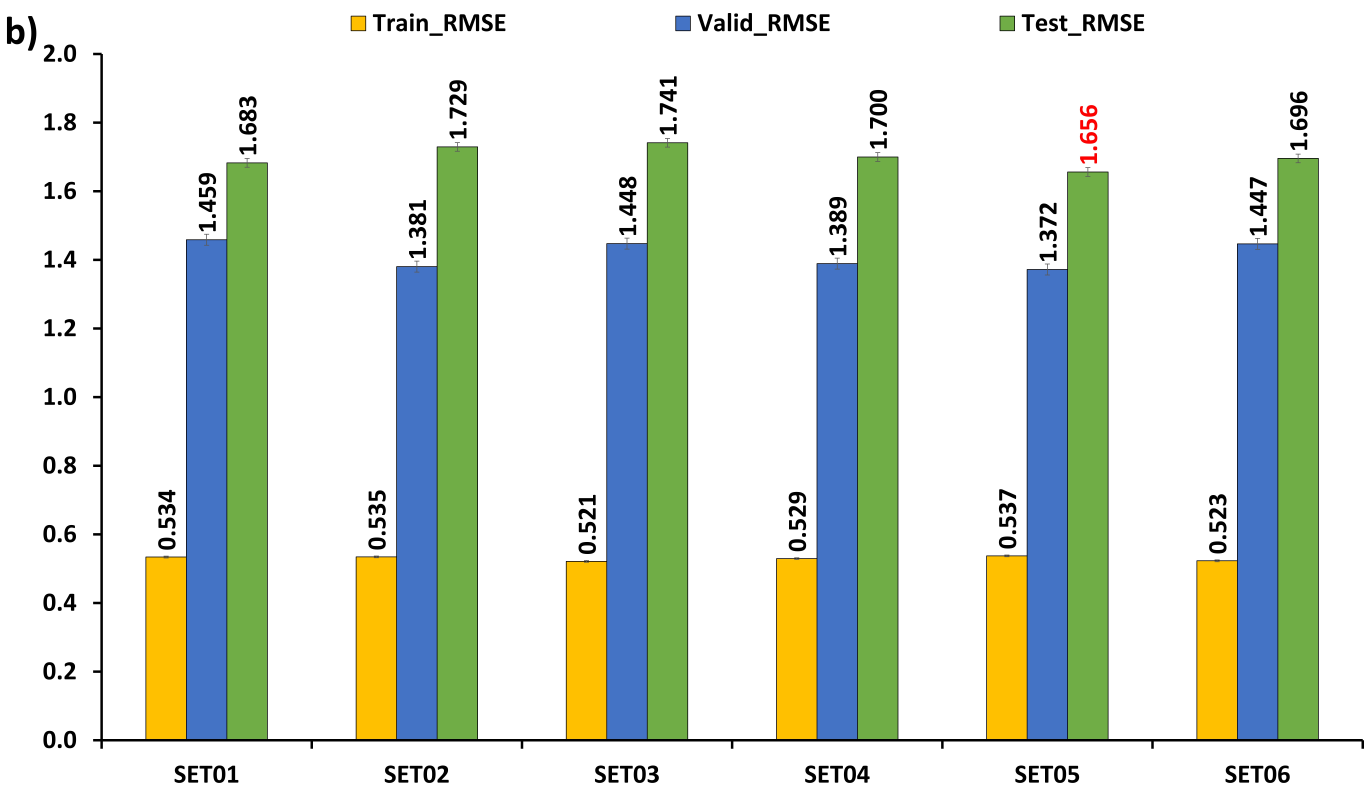


**Figure S5:** Statistical comparison of **a)** PCC (Person-Correlation-Coefficient) and **b)** RMSE (Root-Mean-Square-Error) between different SETS for Train, Valid and Test data based on Interaction Fingerprint Pattern (IFP) and Interaction Distances (IFP+Int-Dist) as features (RF parameters: max_features = 'auto'). The statistics shown in red color are better among other SETS.


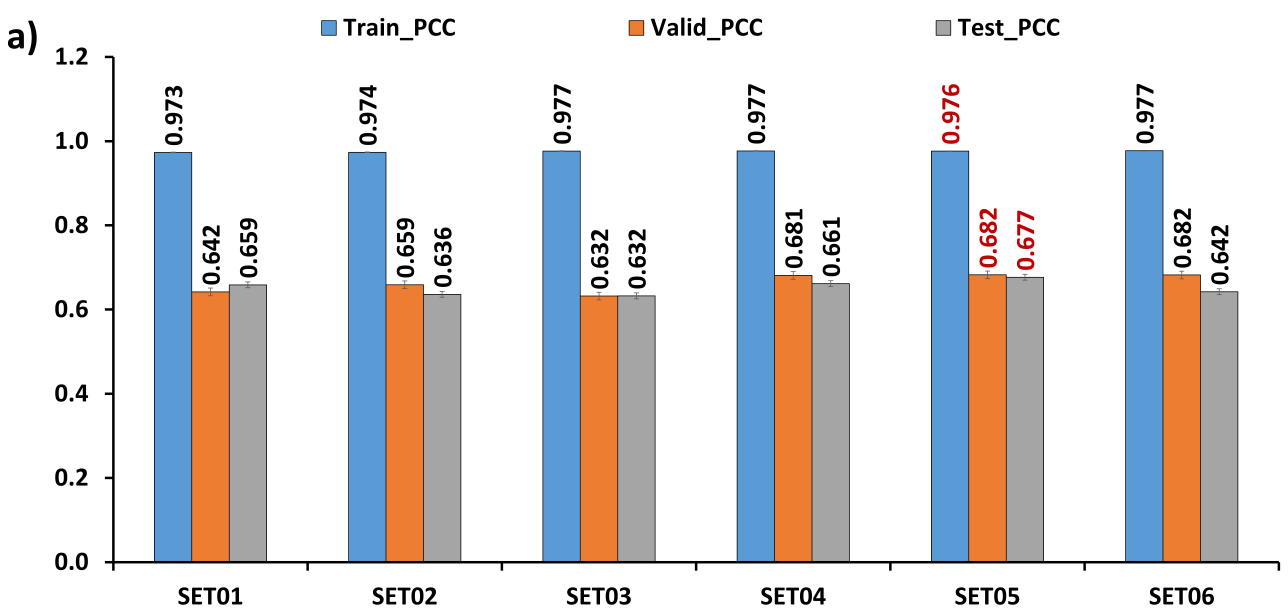


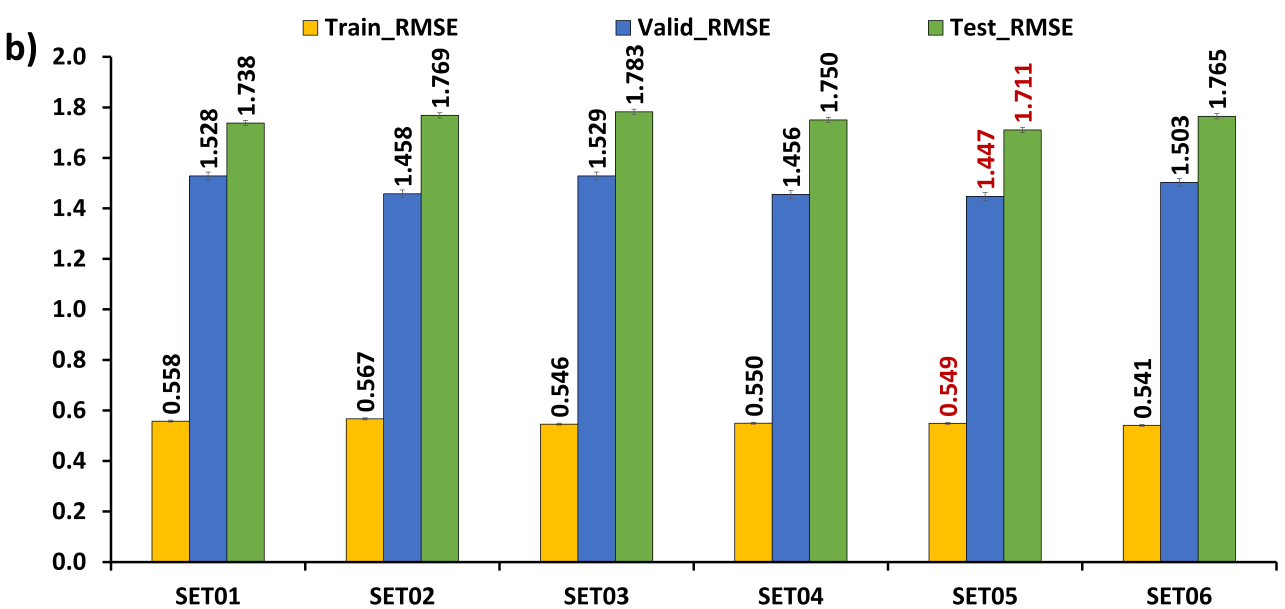


**Figure S6:** Statistical comparison of **a)** PCC (Person-Correlation-Coefficient) and **b)** RMSE (Root-Mean-Square-Error) between different SETS for Train, Valid and Test data based on Interaction Fingerprint Pattern (IFP) and Interaction Distances (IFP+Int-Dist) as features (RF parameters: max_features = 'sqrt'). The statistics shown in red color are better among other SETS.


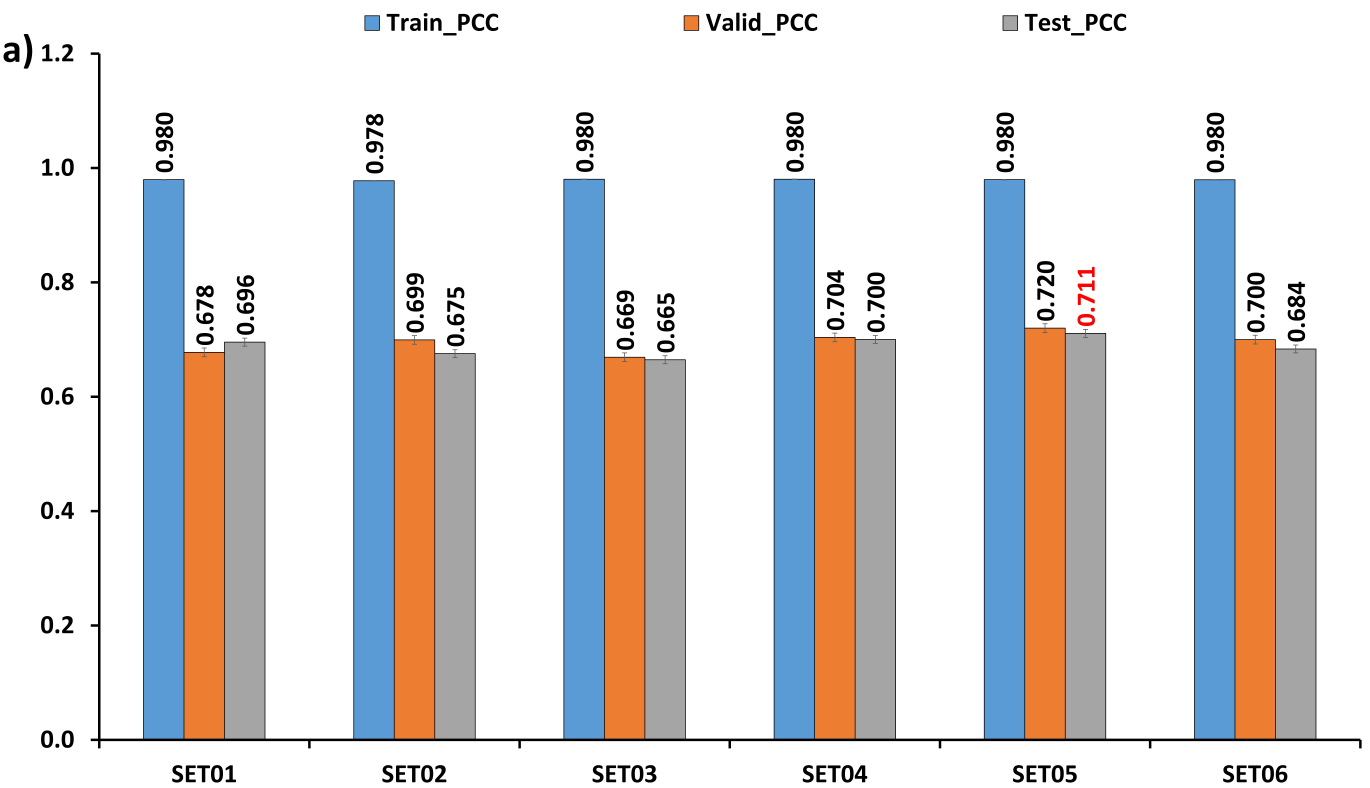


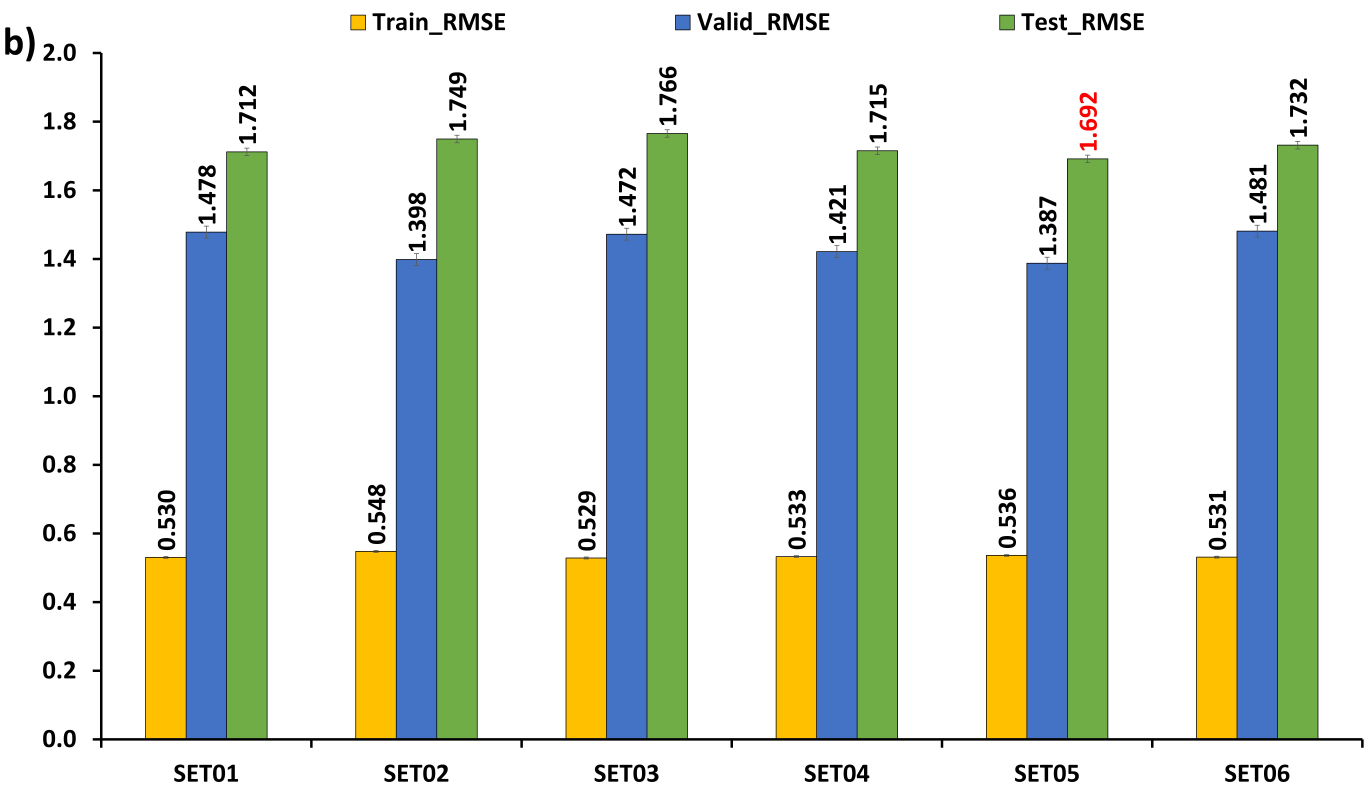


**Figure S7:** Statistical comparison of **a)** PCC (Person-Correlation-Coefficient) and **b)** RMSE (Root-Mean-Square-Error) between different SETS for Train, Valid and Test data based on Interaction Fingerprint Pattern (IFP) and Ligand Fragments (IFP+Frag) as features (RF parameters: max_features = 'auto'). The statistics shown in red color are better among other SETS.


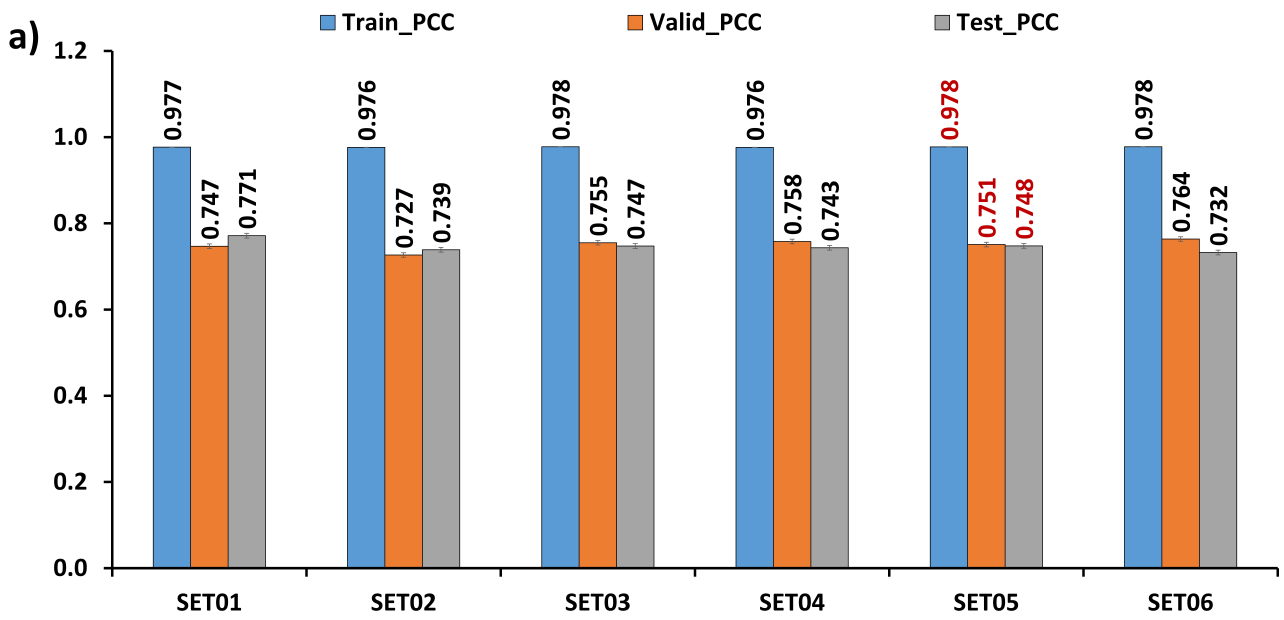


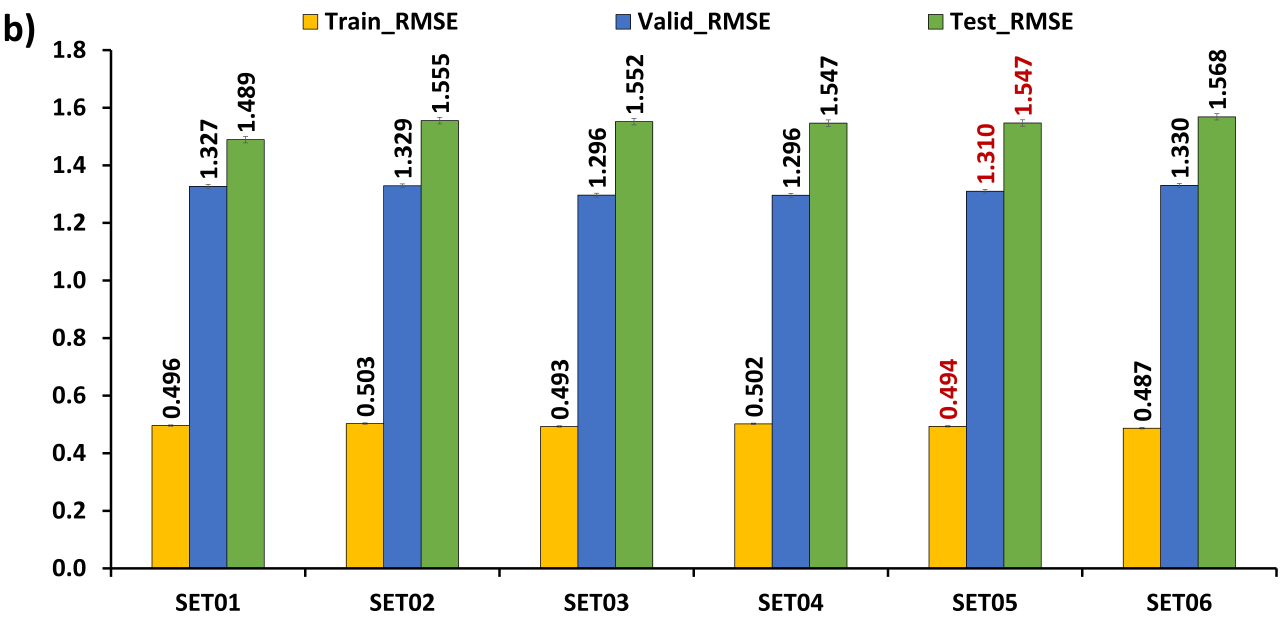


**Figure S8:** Statistical comparison of **a)** PCC (Person-Correlation-Coefficient) and **b)** RMSE (Root-Mean-Square-Error) between different SETS for Train, Valid and Test data based on Interaction Fingerprint Pattern (IFP) and Ligand Fragments (IFP+Frag) as features (RF parameters: max_features = 'sqrt'). The statistics shown in red color are better among other SETS.


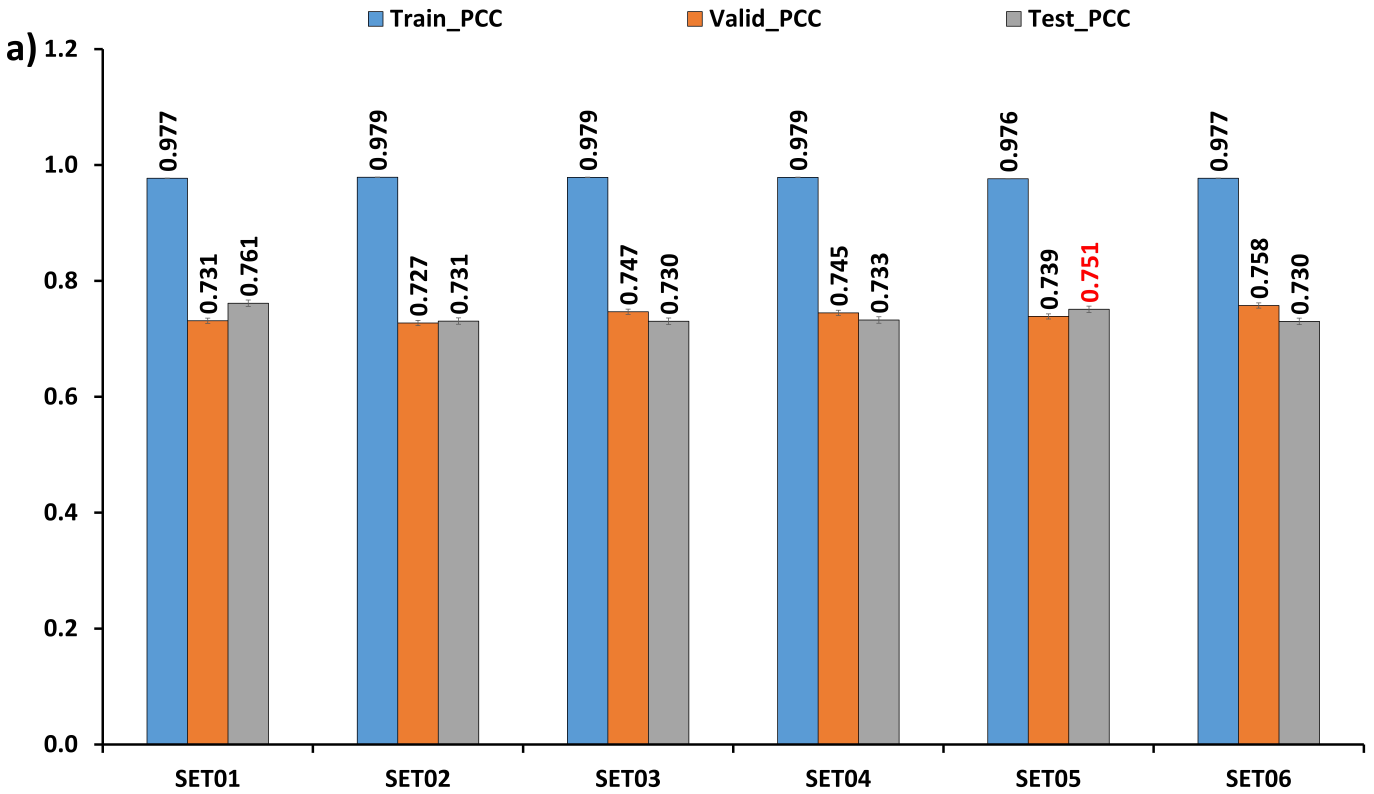


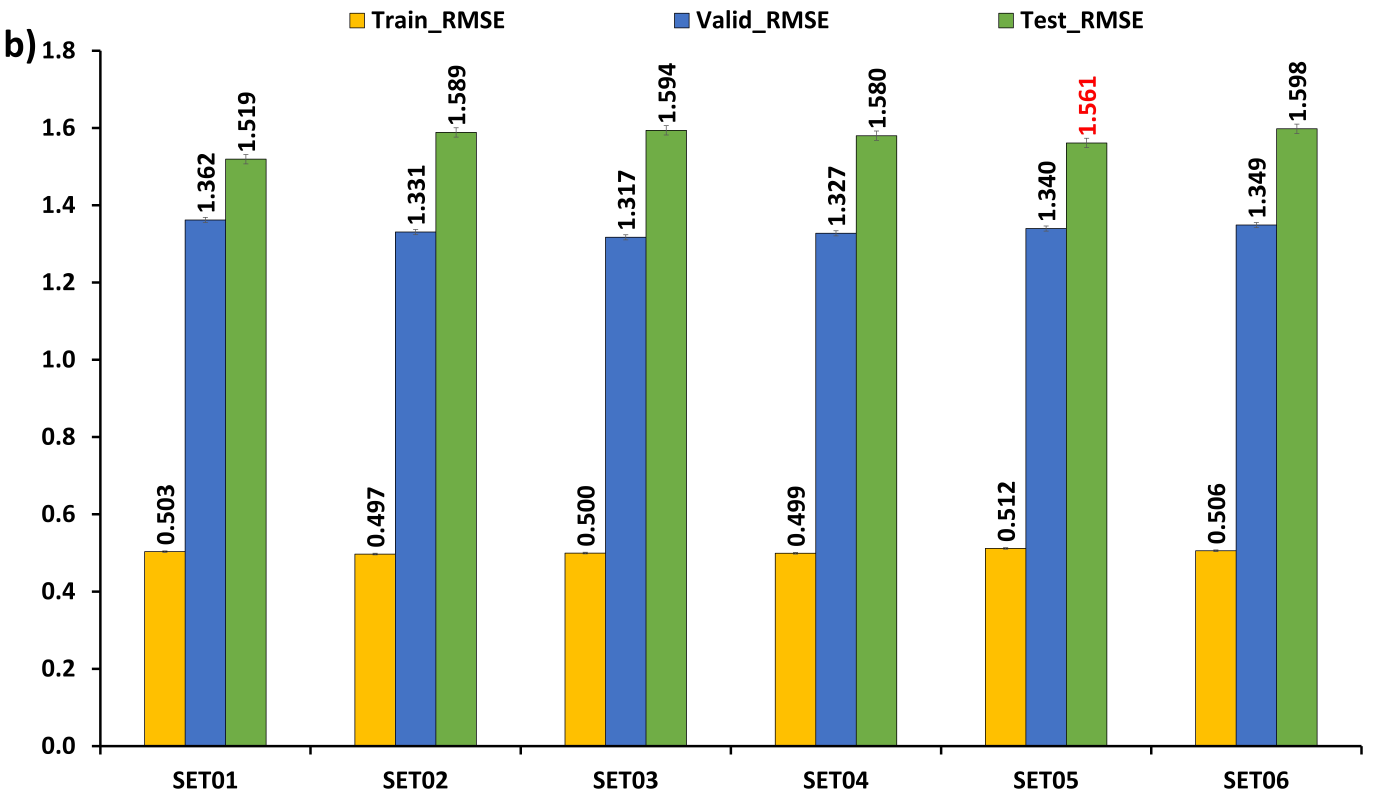


**Figure S9:** Statistical comparison of **a)** PCC (Person-Correlation-Coefficient) and **b)** RMSE (Root-Mean-Square-Error) between different SETS for Train, Valid and Test data based on Interaction Fingerprint Pattern (IFP), Interaction Distances and Ligand Fragments (IFP+Int-Dist+Frag) as features (RF parameters: max_features = 'auto'). The statistics shown in red color are better among other SETS.


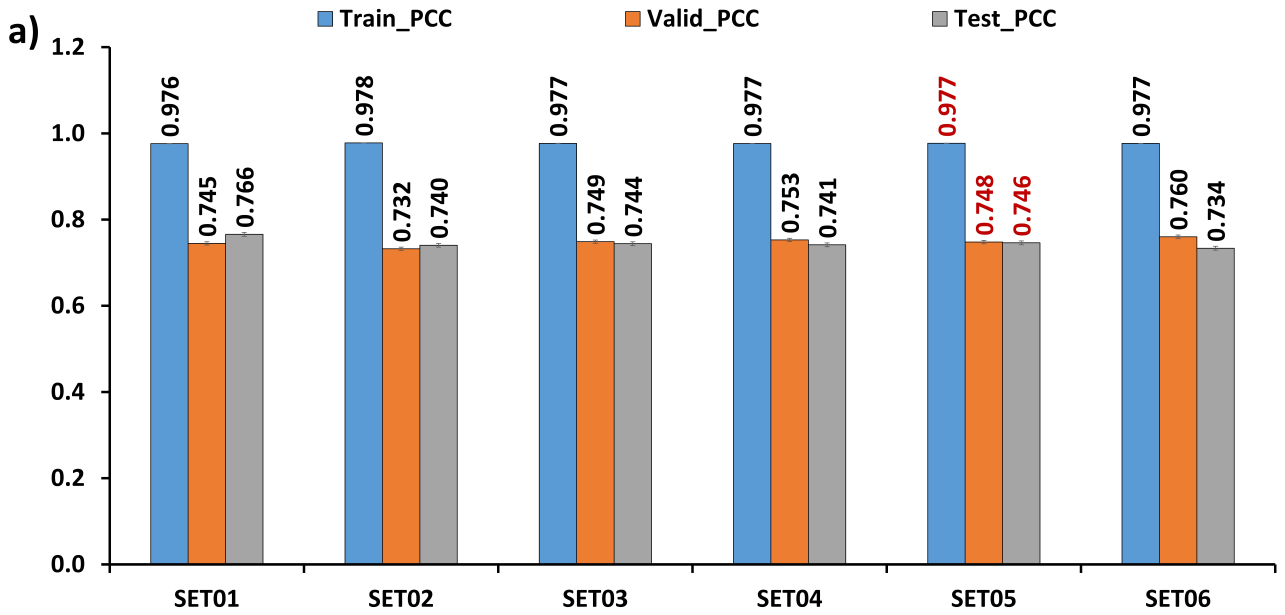


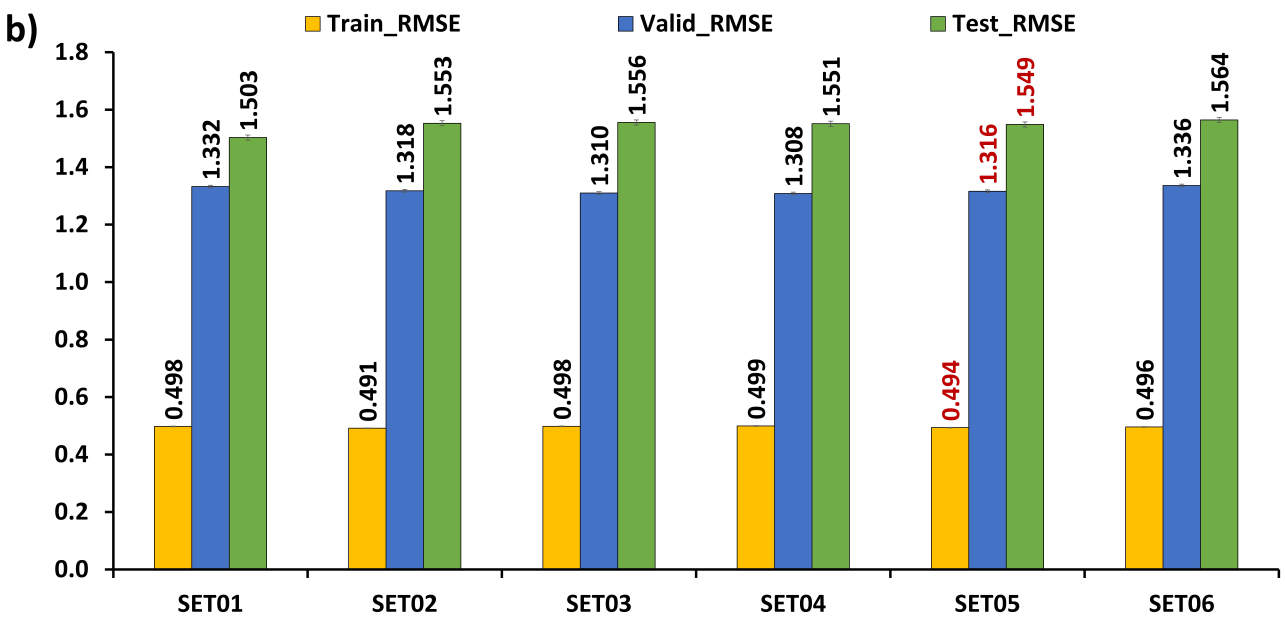


**Figure S10:** Statistical comparison of **a)** PCC (Person-Correlation-Coefficient) and **b)** RMSE (Root-Mean-Square-Error) between different SETS for Train, Valid and Test data based on Interaction Fingerprint Pattern (IFP), Interaction Distances and Ligand Fragments (IFP+Int-Dist+Frag) as features (RF parameters: max_features = 'sqrt'). The statistics shown in red color are better among other SETS.


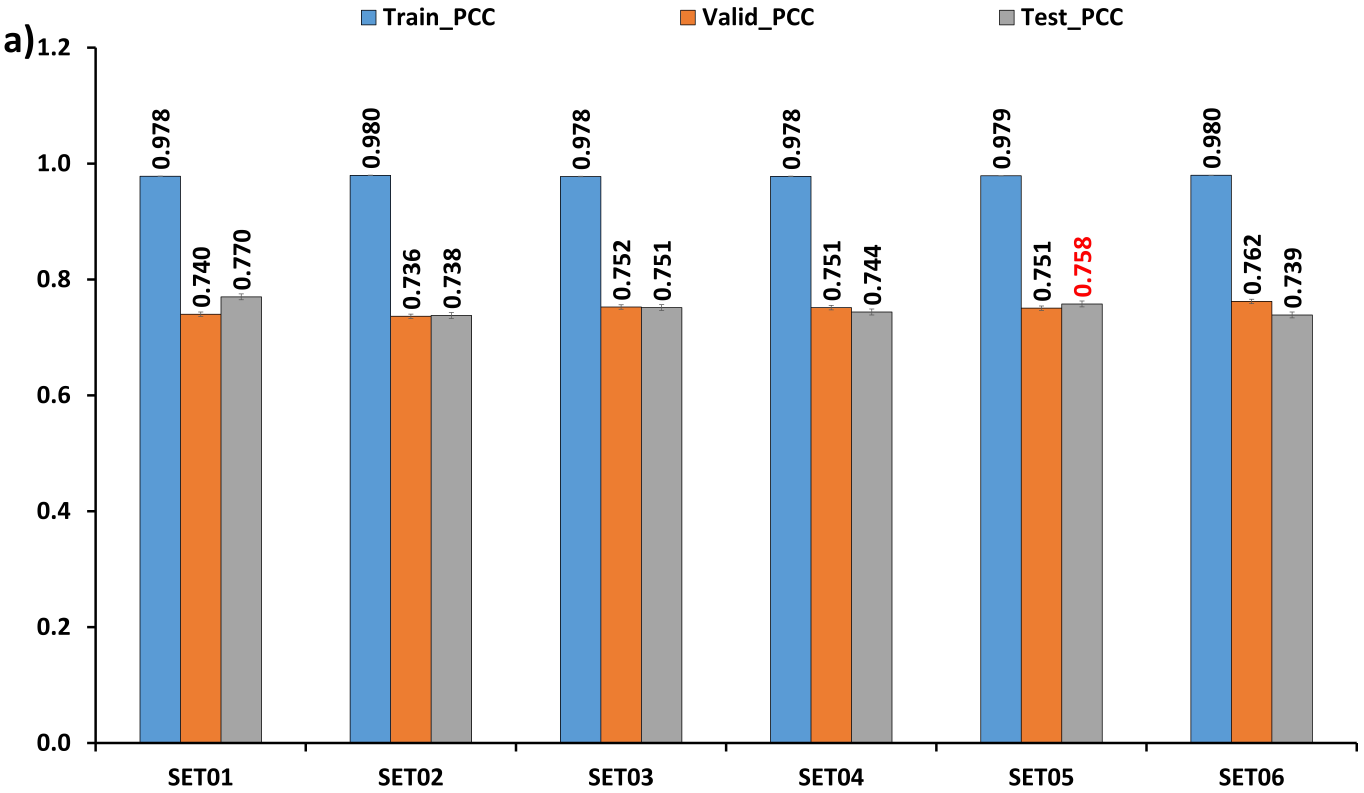


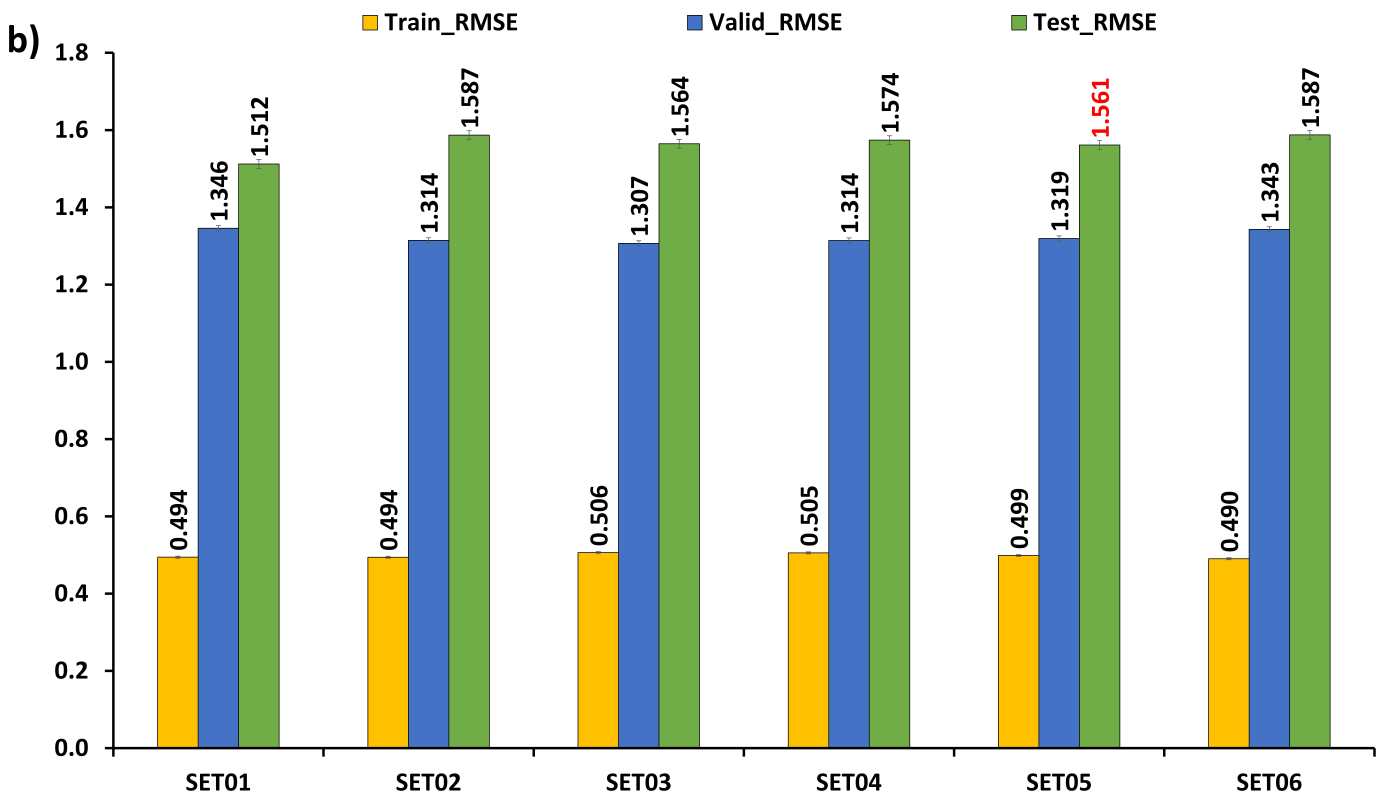


**Table S3:** The Result of statistical performance on PDBbind (Release 2015) using the DNN model. The Refined set (n =3481) used for training and validation, and core set (n=180) as a test set. The valid represent 20% of the refined set. The boldface represents the model with good statistics in each category.

| **Features** | **SETS** | **Model Parameters** | **Best Model**  **at Epochs** | **Train** | | | **Valid** | | | **Test** | | |
| --- | --- | --- | --- | --- | --- | --- | --- | --- | --- | --- | --- | --- |
|  |  |  |  | **LOSS** | **RMSE** | **PCC** | **LOSS** | **RMSE** | **PCC** | **LOSS** | **RMSE** | **PCC** |
| IFP | SET01 | 64_0.3_0.6 | 140 | 0.359 | 0.779 | 0.920 | 0.810 | 1.529 | 0.668 | 0.805 | 1.574 | 0.708 |
|  | SET02 | 128_0.5_0.6 | 212 | 0.554 | 1.133 | 0.832 | 0.790 | 1.481 | 0.670 | 0.839 | 1.644 | 0.697 |
|  | SET03 | 256_0.3_0.4 | 263 | 0.689 | 1.048 | 0.849 | 1.060 | 1.530 | 0.645 | 1.099 | 1.626 | 0.691 |
|  | SET04 | 64_0.1_0.1 | 75 | 0.630 | 0.693 | 0.938 | 1.343 | 1.458 | 0.693 | 1.493 | 1.624 | 0.691 |
|  | **SET05** | **128_0.3_0.4** | **209** | **0.563** | **0.871** | **0.899** | **1.019** | **1.483** | **0.678** | **1.032** | **1.538** | **0.726** |
|  | SET06 | 64_0.1_0.2 | 102 | 0.540 | 0.661 | 0.943 | 1.259 | 1.495 | 0.686 | 1.341 | 1.600 | 0.694 |
| IFP + Int-Dist | SET01 | 128_0.4_0.4 | 156 | 0.703 | 1.070 | 0.849 | 1.065 | 1.541 | 0.650 | 1.079 | 1.606 | 0.712 |
|  | SET02 | 64_0.2_0.4 | 79 | 0.561 | 0.871 | 0.904 | 0.964 | 1.401 | 0.692 | 1.088 | 1.613 | 0.698 |
|  | SET03 | 128_0.3_0.1 | 120 | 0.931 | 1.019 | 0.867 | 1.342 | 1.455 | 0.675 | 1.507 | 1.641 | 0.692 |
|  | SET04 | 64_0.5_0.4 | 103 | 0.758 | 1.147 | 0.826 | 0.997 | 1.453 | 0.686 | 1.100 | 1.638 | 0.707 |
|  | **SET05** | **128_0.5_0.6** | **261** | **0.472** | **0.990** | **0.873** | **0.775** | **1.468** | **0.687** | **0.805** | **1.582** | **0.713** |
|  | SET06 | 128_0.3_0.1 | 127 | 0.932 | 1.020 | 0.860 | 1.383 | 1.501 | 0.681 | 1.530 | 1.664 | 0.680 |
| IFP+ Frag | SET01 | 128_0.5_0.2 | 143 | 0.764 | 0.929 | 0.895 | 1.235 | 1.464 | 0.679 | 1.333 | 1.593 | 0.709 |
|  | **SET02** | **64_0.1_0.7** | **129** | **0.209** | **0.595** | **0.956** | **0.631** | **1.402** | **0.699** | **0.646** | **1.530** | **0.733** |
|  | SET03 | 64_0.2_0.1 | 66 | 0.762 | 0.837 | 0.910 | 1.279 | 1.389 | 0.712 | 1.423 | 1.551 | 0.727 |
|  | SET04 | 64_0.2_0.5 | 129 | 0.392 | 0.718 | 0.934 | 0.872 | 1.436 | 0.693 | 0.918 | 1.557 | 0.720 |
|  | SET05 | 128_0.2_0.1 | 269 | 0.486 | 0.536 | 0.964 | 1.216 | 1.323 | 0.747 | 1.432 | 1.560 | 0.716 |
|  | SET06 | 64_0.3_0.4 | 111 | 0.492 | 0.769 | 0.924 | 0.963 | 1.424 | 0.727 | 1.079 | 1.598 | 0.701 |
| IFP + Frag + Int-Dist | SET01 | 128_0.4_0.4 | 156 | 0.703 | 1.070 | 0.849 | 1.065 | 1.541 | 0.650 | 1.079 | 1.606 | 0.712 |
|  | SET02 | 64_0.2_0.4 | 79 | 0.561 | 0.871 | 0.904 | 0.964 | 1.401 | 0.692 | 1.088 | 1.613 | 0.698 |
|  | SET03 | 128_0.3_0.1 | 120 | 0.931 | 1.019 | 0.867 | 1.342 | 1.455 | 0.675 | 1.507 | 1.641 | 0.692 |
|  | SET04 | 64_0.5_0.4 | 103 | 0.758 | 1.147 | 0.826 | 0.997 | 1.453 | 0.686 | 1.100 | 1.638 | 0.707 |
|  | SET05 | 128_0.5_0.6 | 261 | 0.472 | 0.990 | 0.873 | 0.775 | 1.468 | 0.687 | 0.805 | 1.582 | 0.713 |
|  | **SET06** | **128_0.1_0.5** | **265** | **0.212** | **0.403** | **0.979** | **0.834** | **1.400** | **0.733** | **0.923** | **1.559** | **0.714** |

**Note:** Random_State assigned for different Sets are as SET01: 123456; SET02: 42; SET03: 56789; SET04: 98765; SET05: 4321; SET06: 1234 to reproduce the same statistical result for training and validation data. **LOSS:** Loss function is defined as: LOSS = α (1-PCC) + (1- α) RMSE; **RMSE:** Root-Mean-Square-Error; **PCC:** Pearson Correlation Coefficient; **Model Parameters:** each value represent the following order: batchsize_dropout_alpha; **Best Model at Epochs:** During training, best model is obtained at the Epochs, when the validation loss improves from the previous epochs. To avoid biasness, the same train, valid, and test set were used for DNN modeling.

**Figure S11:** Statistical comparison of PCC (Person-Correlation-Coefficient) and RMSE (Root-Mean-Square-Error) between different SETS for Train, Valid, and Test data based on Interaction Fingerprint Pattern (IFP) as features. The statistics shown in red color are better among other SETS.


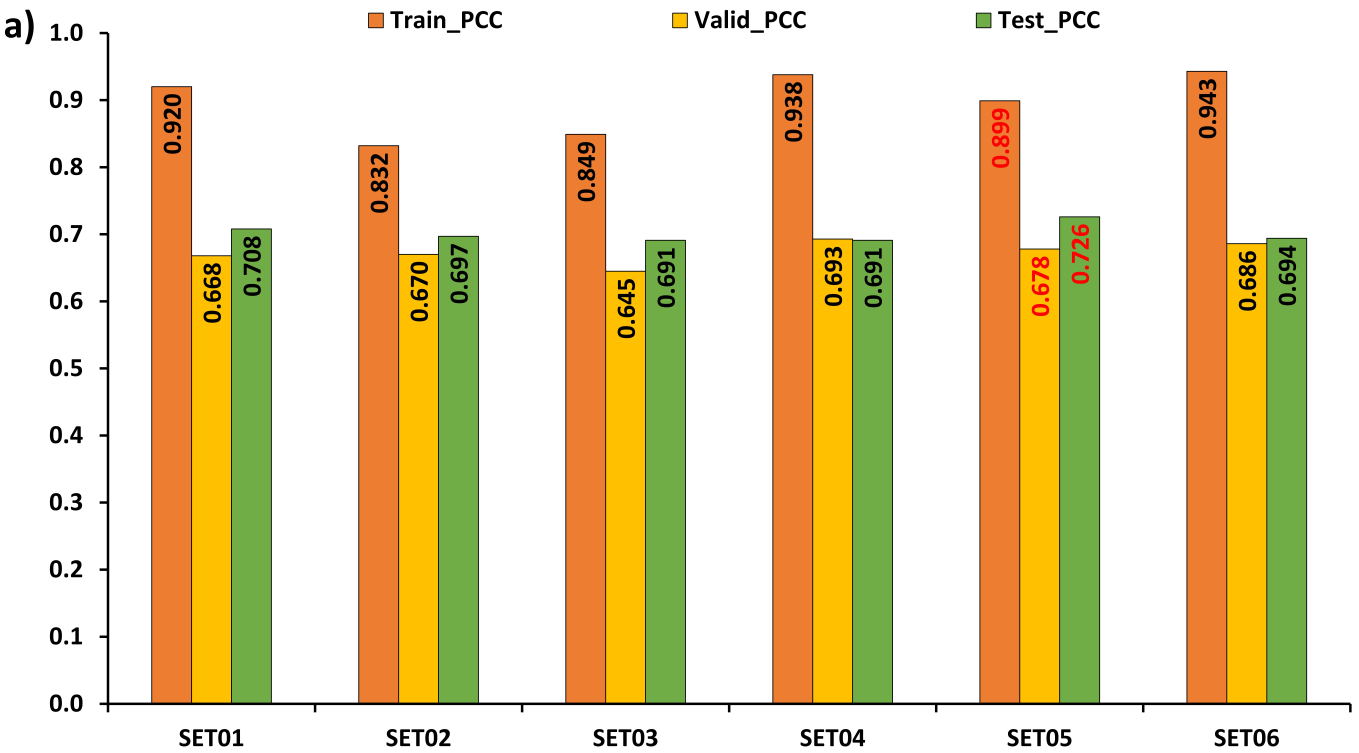


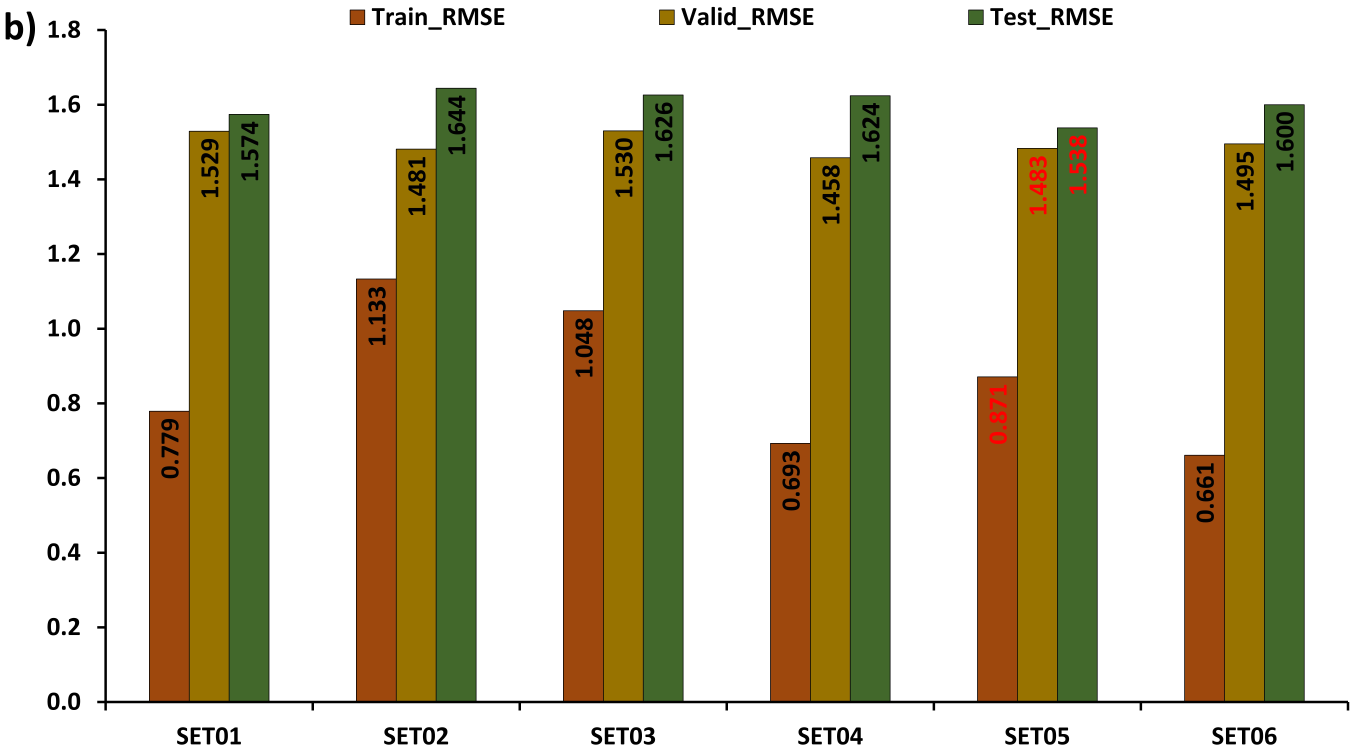


**Figure S12:** Statistical comparison of PCC (Person-Correlation-Coefficient) and RMSE (Root-Mean-Square-Error) between different SETS for Train, Valid and Test data based on Interaction Fingerprint Pattern (IFP) and Interaction-Distance (IFP+Int-Dist) as features. The statistics shown in red color are better among other SETS.


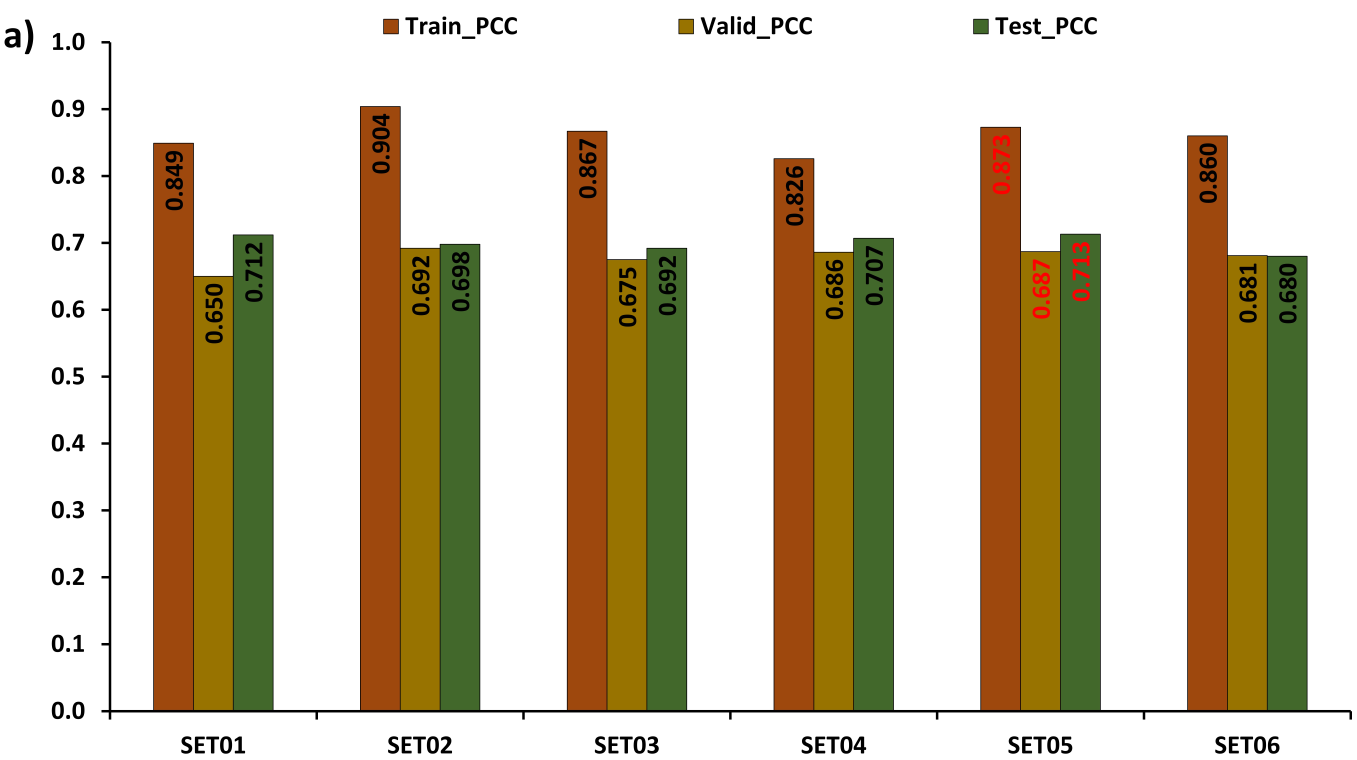


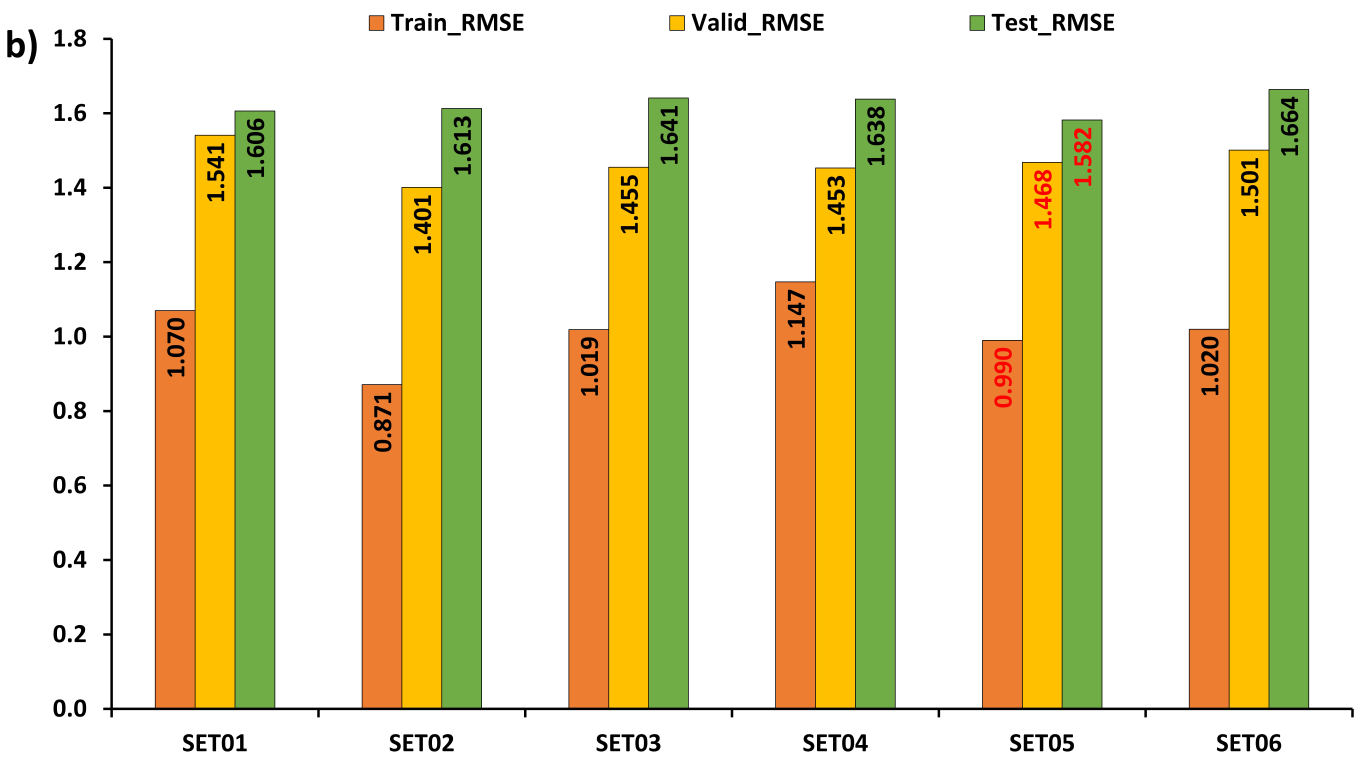


**Figure S13:** Statistical comparison of PCC (Person-Correlation-Coefficient) and RMSE (Root-Mean-Square-Error) between different SETS for Train, Valid and Test data based on Interaction Fingerprint Pattern (IFP) and Ligand fragments (IFP+Frag) as features. The statistics shown in red color are better among other SETS.


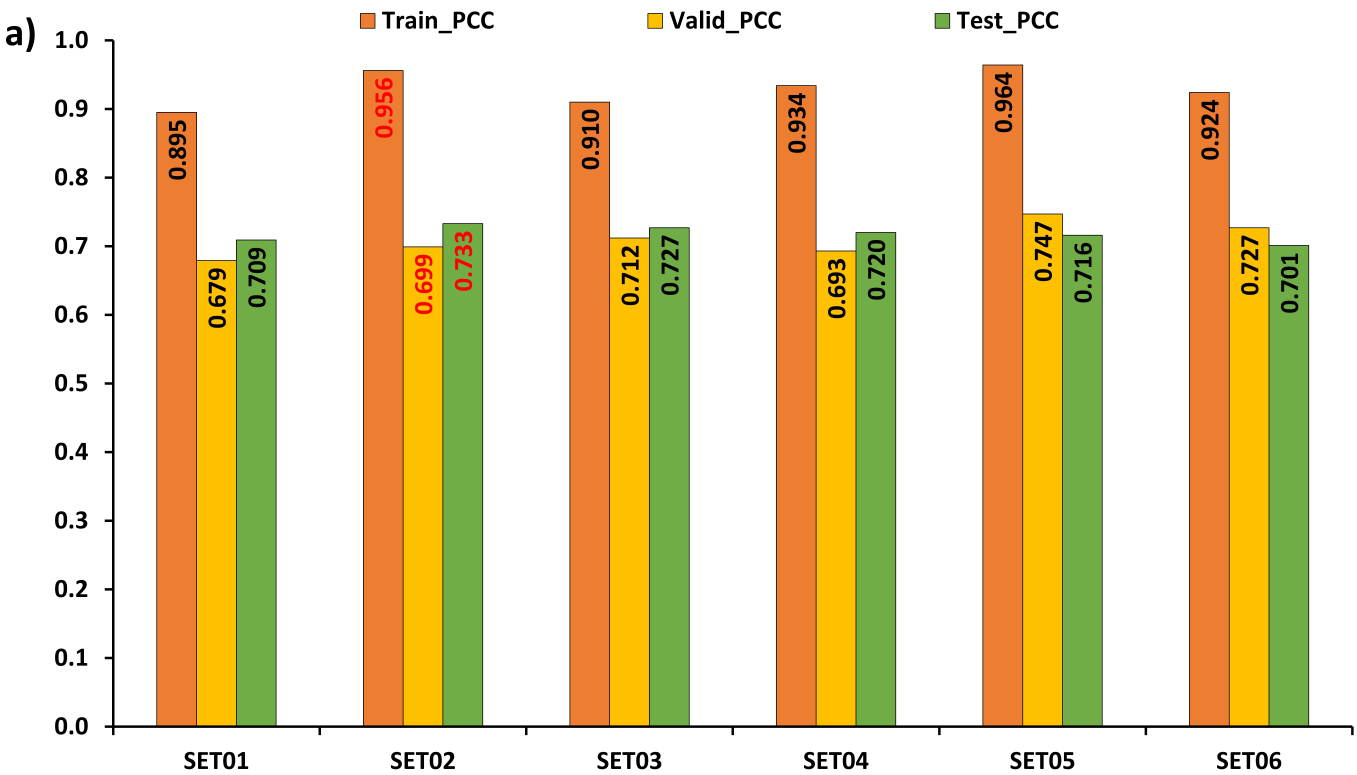


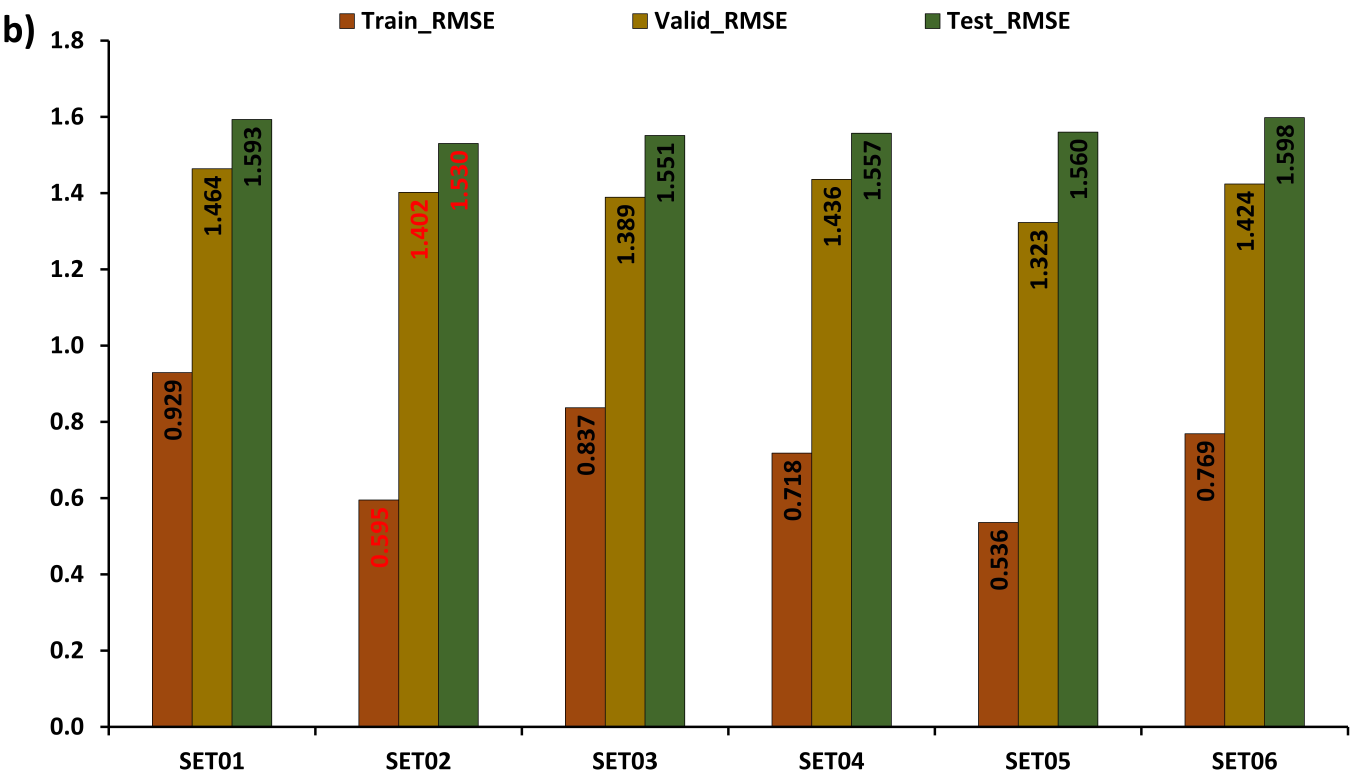


**Figure S14:** Statistical comparison of PCC (Person-Correlation-Coefficient) and RMSE (Root-Mean-Square-Error) between different SETS for Train, Valid and Test data based on Interaction Fingerprint Pattern (IFP), Interaction Distances and Ligand fragments (IFP+Int-Dist_Frag) as features. The statistics shown in red color are better among other SETS.


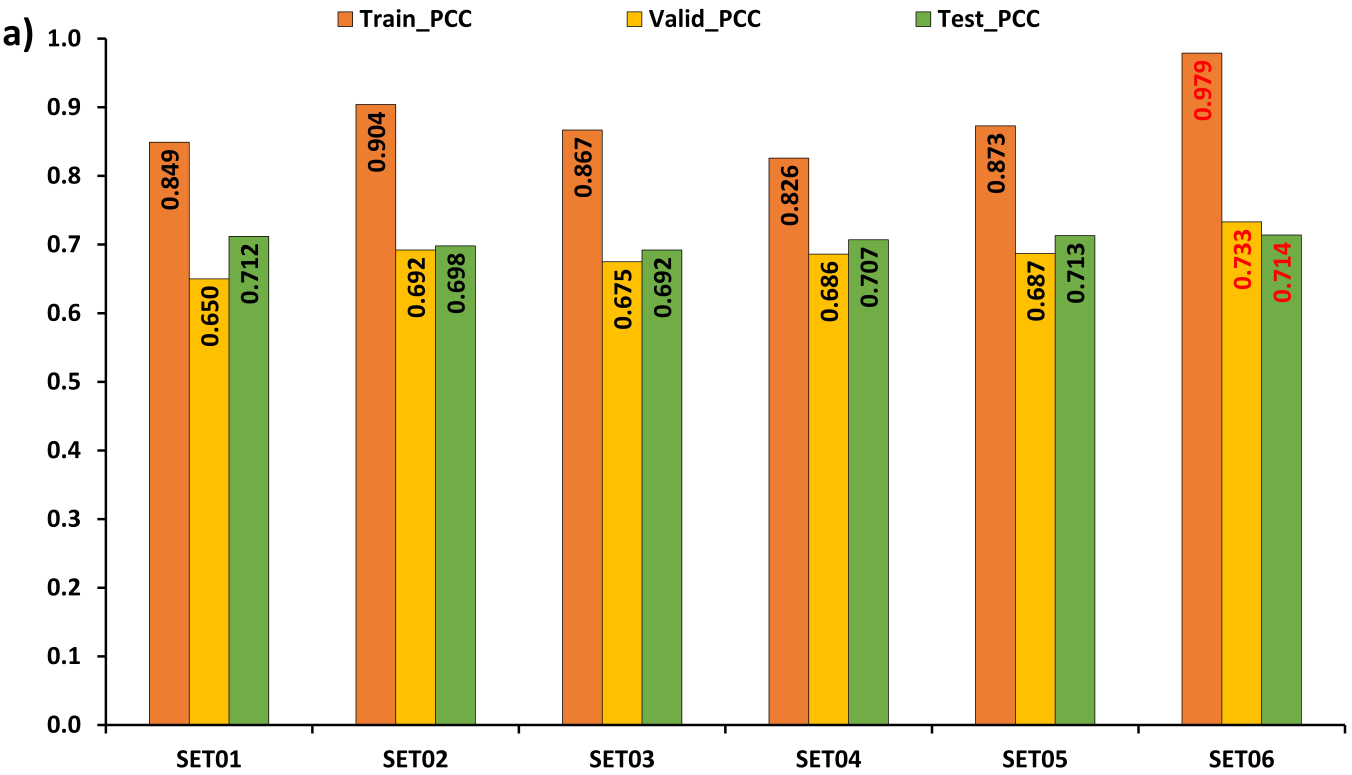


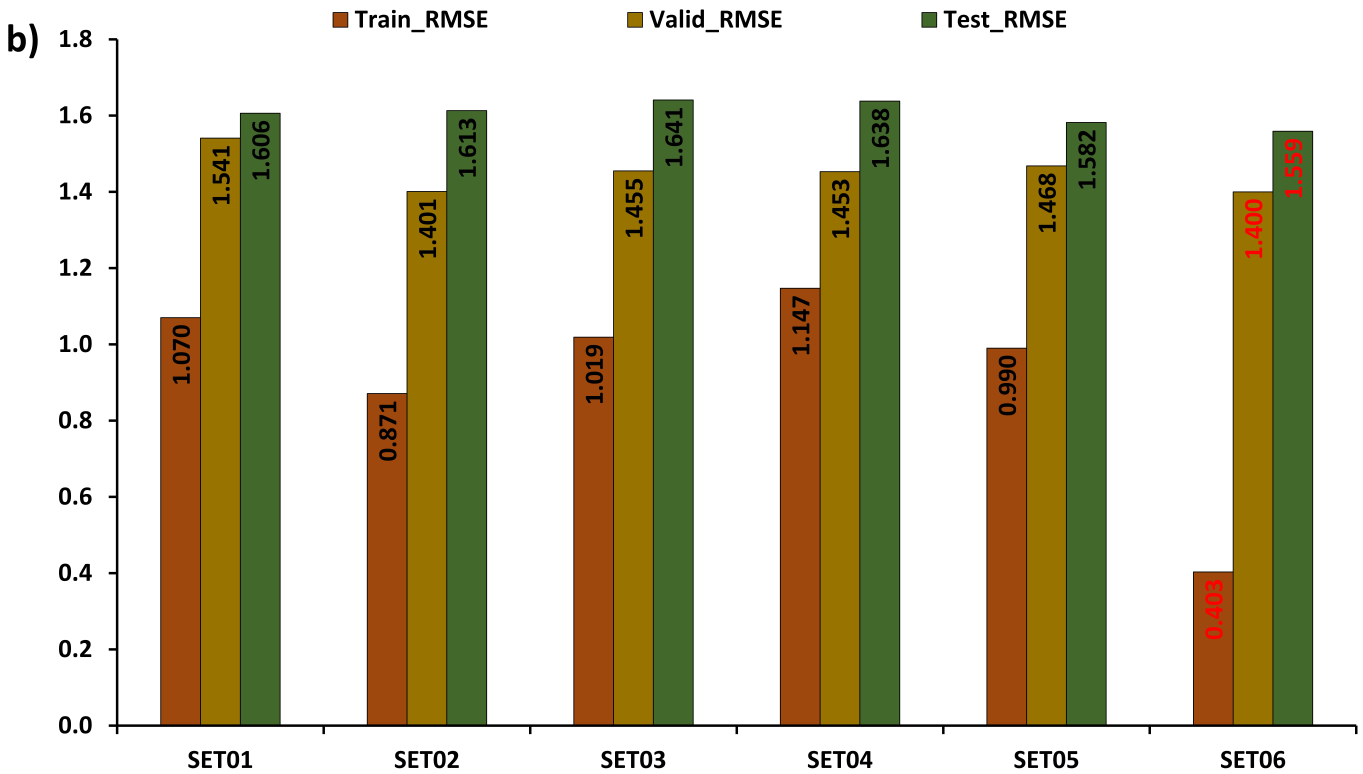


**Figure S15:** Statistical performance of PCC (Person-Correlation-Coefficient) and RMSE (Root-Mean-Square-Error) for Train and Valid data as a function of Epochs based on Interaction Fingerprint Pattern and Ligand fragments (IFP+Frag) as features. (The result shown here is based on selected best Hyperparameter option (Batch_Size: 64; dropout: 0.1; Alpha: 0.7)


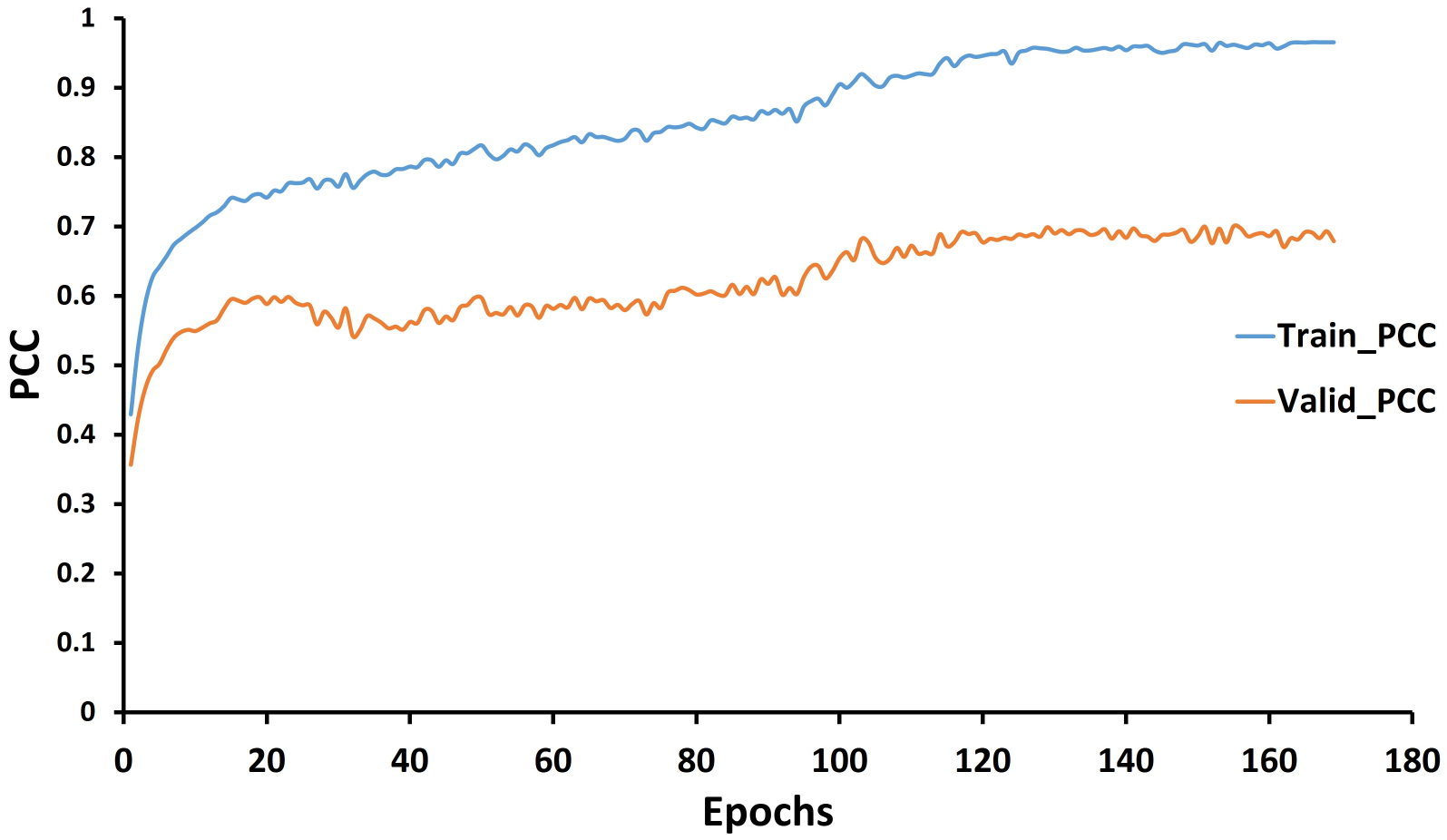


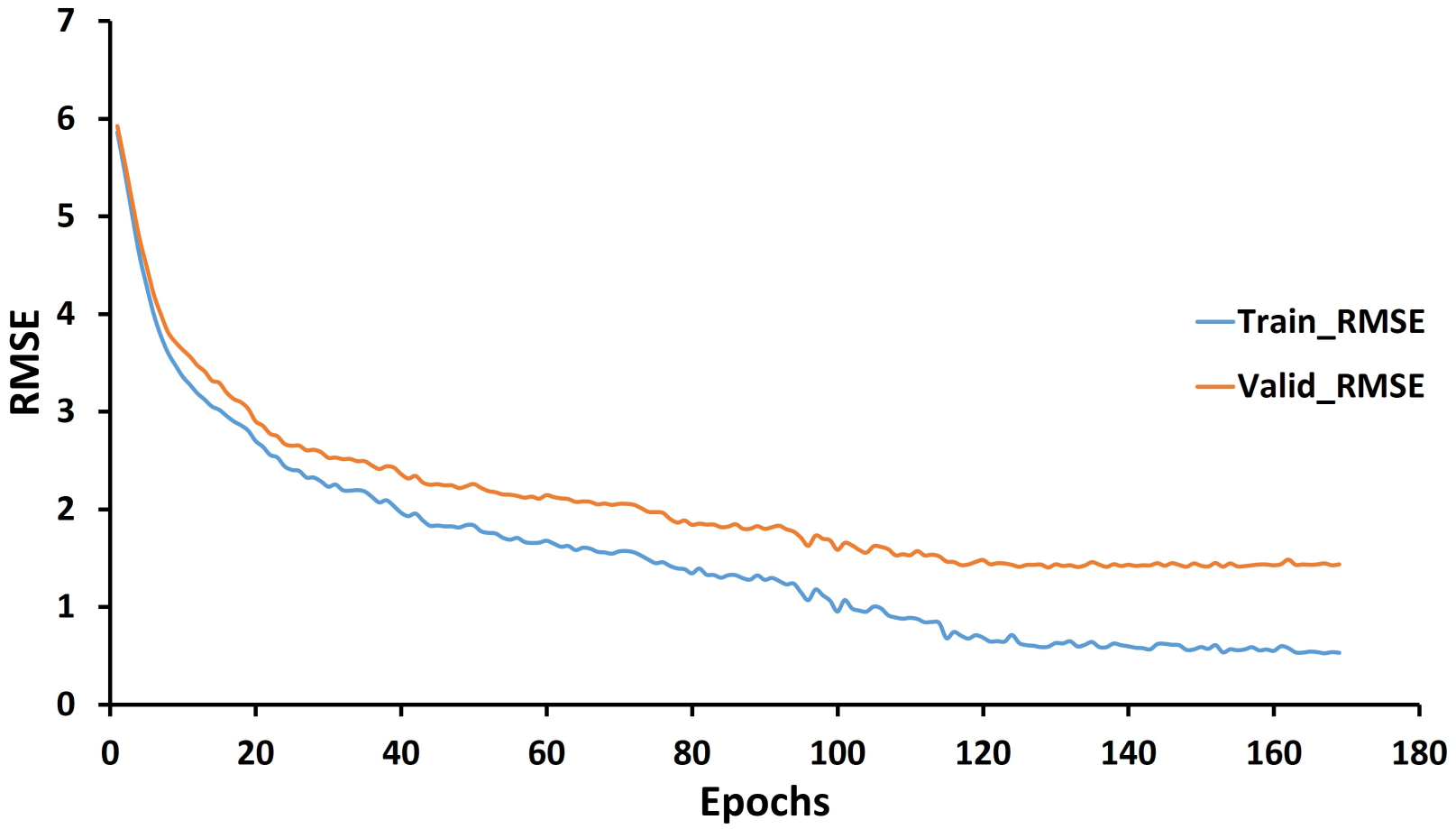


**Table S4:** The variance analysis of the Random Forest (RF) and Deep Neural Network (DNN) models. The results were based on the six best models from test set.

| **Features** | **Methods** | **Parameters** | **RMSE** | | **PCC** | |
| --- | --- | --- | --- | --- | --- | --- |
|  |  |  | **Average** | **Std.** | **Average** | **Std.** |
| **IFP** | **RF** | n_estimators = 500 max_features = 'auto' | 1.707 | 0.033 | 0.663 | 0.021 |
|  |  | n_estimators = 500 max_features = 'sqrt' | 1.701 | 0.031 | 0.693 | 0.019 |
| **IFP + Int_Dist** |  | n_estimators = 500 max_features = 'auto' | 1.753 | 0.026 | 0.651 | 0.017 |
|  |  | n_estimators = 500 max_features = 'sqrt' | 1.728 | 0.027 | 0.689 | 0.017 |
| **IFP + Frag** |  | n_estimators = 500 max_features = 'auto' | 1.543 | 0.028 | 0.747 | 0.013 |
|  |  | n_estimators = 500 max_features = 'sqrt' | 1.574 | 0.030 | 0.739 | 0.013 |
| **IFP + Int_Dist + Frag** |  | n_estimators = 500 max_features = 'auto' | 1.546 | 0.022 | 0.745 | 0.011 |
|  |  | n_estimators = 500 max_features = 'sqrt' | 1.564 | 0.028 | 0.750 | 0.012 |
| **IFP** | **DNN** | - | 1.601 | 0.039 | 0.701 | 0.014 |
| **IFP + Int_Dist** |  |  | 1.624 | 0.029 | 0.700 | 0.013 |
| **IFP + Frag** |  |  | 1.565 | 0.026 | 0.718 | 0.012 |
| **IFP + Int_Dist + Frag** |  |  | 1.607 | 0.032 | 0.706 | 0.009 |

**Abbreviations: RMSE:** Root-Mean-Square-Error; **PCC:** Pearson Correlation Coefficient; **RF:** Random Forest**; DNN:** Deep Neural Network

**Figure S16:** The binding affinity predictions for the Five benchmark dataset with IFP+Frag features using Random Forest Model. **a)** Astex Diverse Set; **b)** CASR NRC HiQ Set01; **c)** CASR NRC HiQ Set02; **d)** CSAF-2016; **e)** PDBbind NMR; and FEP dataset **f)** BACE; **g)** MCL1; **h)** PTP1B; **i)** Thrombin; **j)** Tyk2


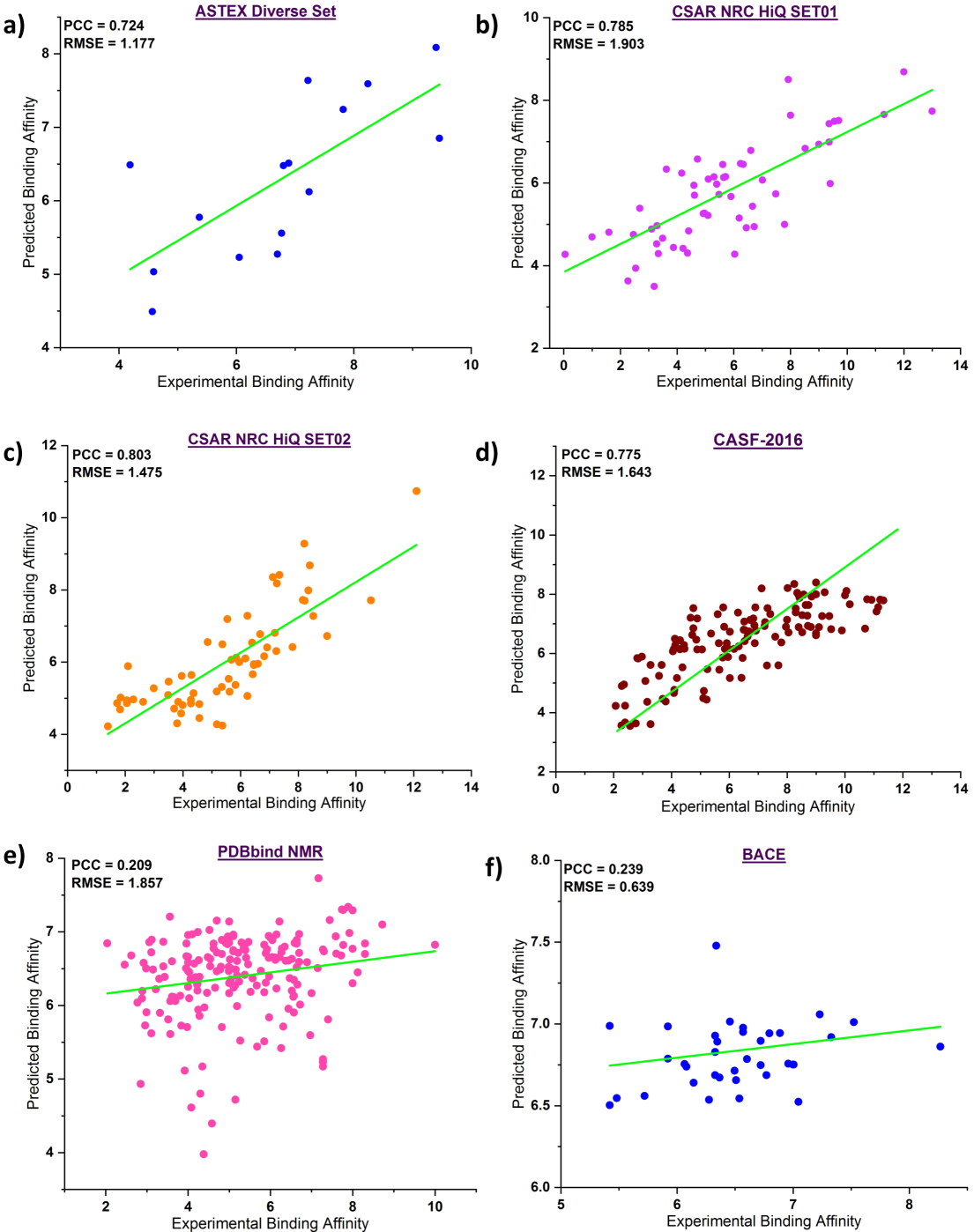


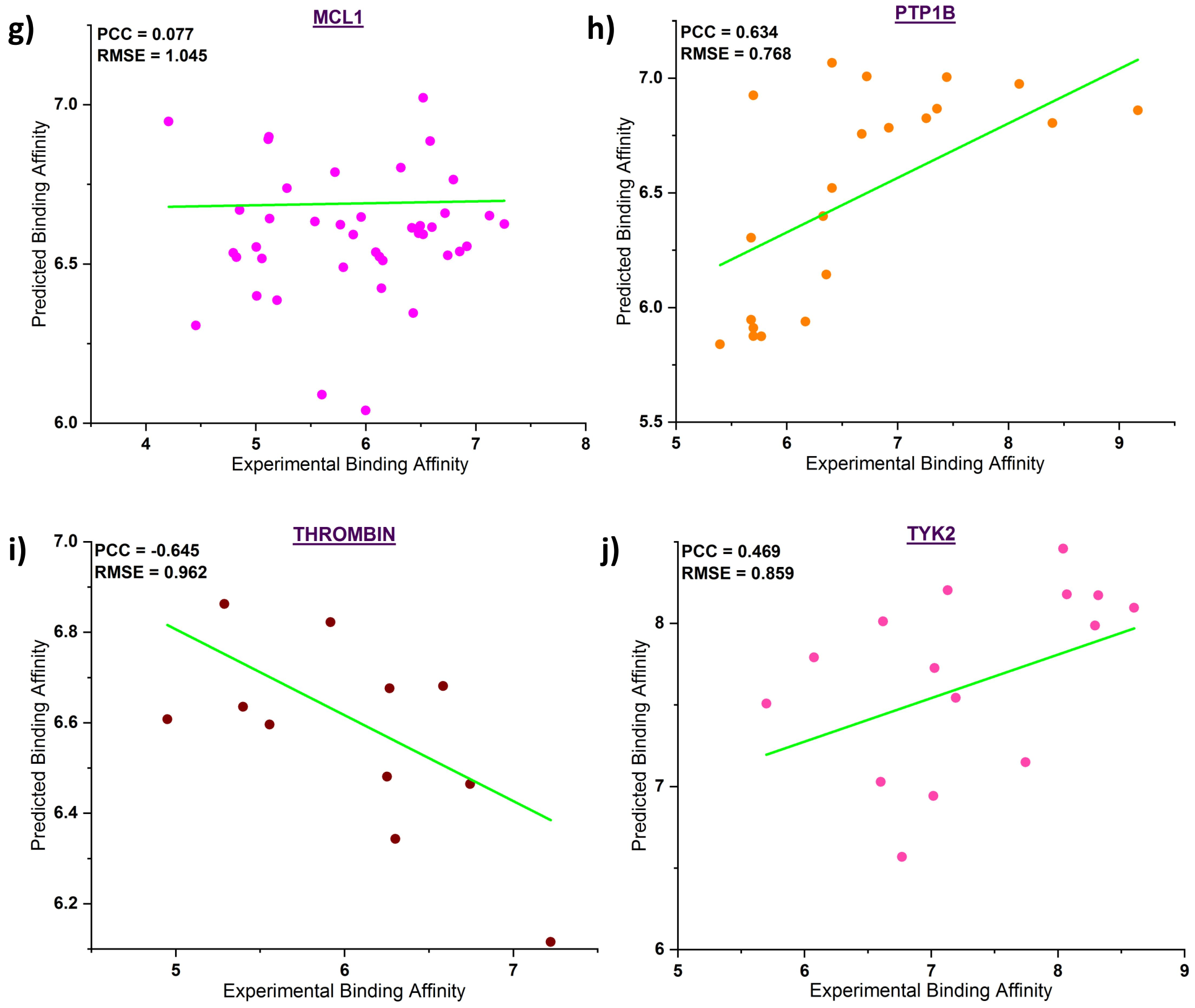


**Figure S17:** The superimposition of docked pose (magenta color) over the crystal pose (green color) for **a)** PDB ID: 2JDM; **b)** PDB ID: 3VH9 from PDBbind Core Set. The nitrogen atoms are in blue, oxygen in red, and hydrogen in white color.


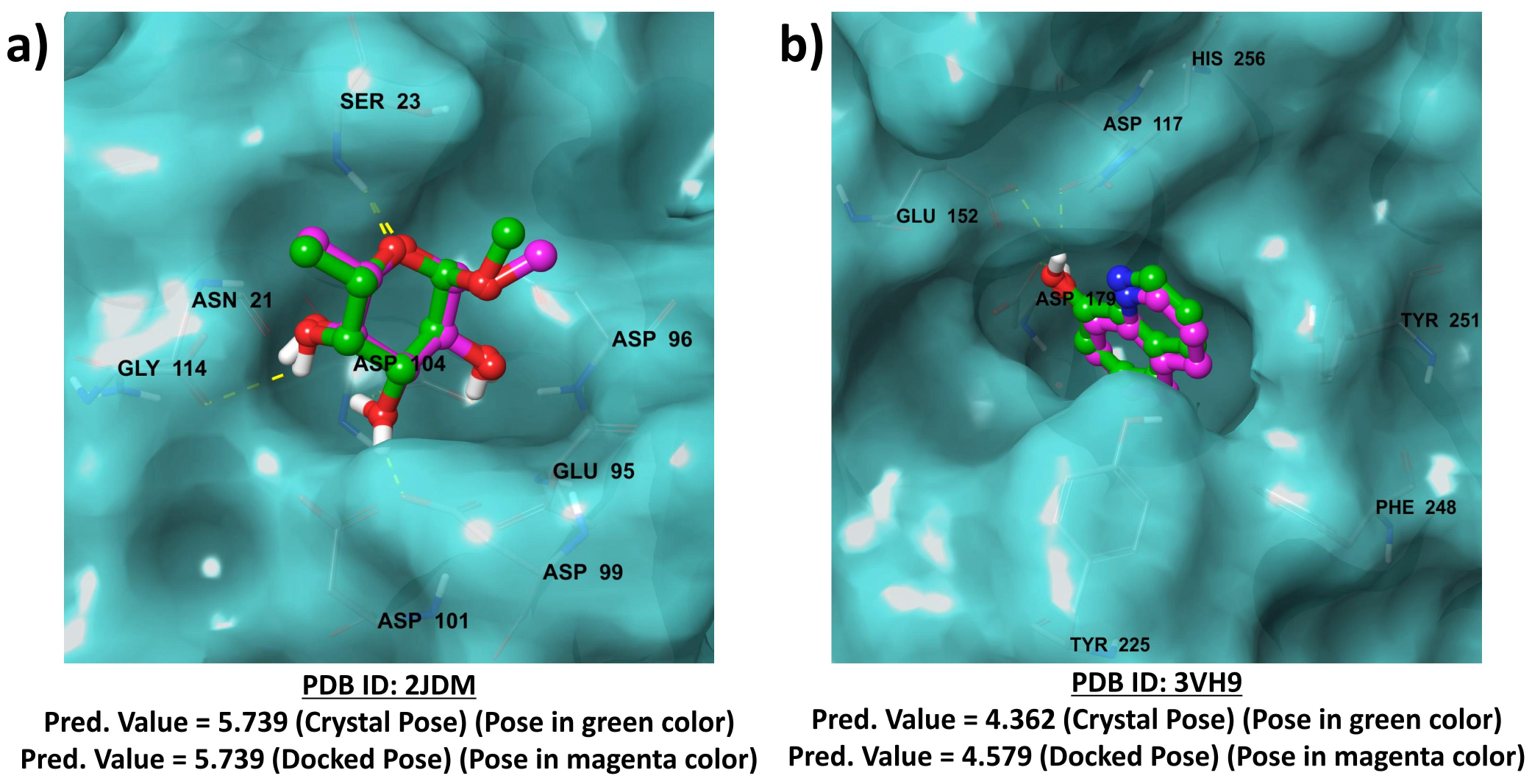


**Table S5:** Interaction Fingerprint Pattern (IFP) calculated for Crystal pose and Docked pose for PDB (**2JDM**).

| **Interaction Fingerprint Pattern** | | | | | | |
| --- | --- | --- | --- | --- | --- | --- |
| **Crystal Pose** | | |  | **Docked Pose** | | |
| **Hydrophobic** | **CB** | **ALA_22_** |  | **Hydrophobic** | **CB** | **ALA_22_** |
| **Hydrophobic** | **CB** | **SER_23_** |  | **Hydrophobic** | **CB** | **SER_23_** |
| **Hydrophobic** | **CG2** | **THR_45_** |  | **Hydrophobic** | **CG2** | **THR_45_** |
| **HBond_LIG** | **OD1** | **ASP_104_** |  | **HBond_LIG** | **OD1** | **ASP_104_** |
| **HBond_LIG** | **OD2** | **ASP_104_** |  | **HBond_LIG** | **OD2** | **ASP_104_** |
| **HBond_LIG** | **OD1** | **ASP_104_** |  | **HBond_LIG** | **OD1** | **ASP_104_** |
| **HBond_LIG** | **O** | **GLY_114_** |  | **HBond_LIG** | **O** | **GLY_114_** |
| **HBond_PROT** | **N** | **SER_23_** |  | **HBond_PROT** | **N** | **SER_23_** |

**Note:** Boldface represents the common interaction found in Crystal Pose and Docked Pose.

**Table S6:** Interaction Fingerprint Pattern (IFP) calculated for Crystal pose and Docked pose for PDB (**3VH9**).

| **Interaction Fingerprint Pattern** | | | | | | |
| --- | --- | --- | --- | --- | --- | --- |
| **Crystal Pose** | | |  | **Docked Pose** | | |
| **Hydrophobic** | **CB** | **ASP_179_** |  | **Hydrophobic** | **CB** | **ASP_179_** |
| **Hydrophobic** | **SD** | **MET_180_** |  | **Hydrophobic** | **SD** | **MET_180_** |
| **Hydrophobic** | **CB** | **CYS_227_** |  | Hydrophobic | SG | CYS_223_ |
| **Hydrophobic** | **CE** | **MET_242_** |  | **Hydrophobic** | **SG** | **CYS_227_** |
| **Hydrophobic** | **CD1** | **ILE_255_** |  | **Hydrophobic** | **CB** | **CYS_227_** |
| HBond_LIG | OE2 | GLU_152_ |  | **Hydrophobic** | **CE** | **MET_242_** |
|  |  |  |  | **Hydrophobic** | **CD1** | **ILE_255_** |
|  |  |  |  | HBond_LIG | OD1 | ASP_117_ |
|  |  |  |  | HBond_LIG | OD1 | ASP_179_ |

**Note:** Boldface represents the common interaction found in Crystal Pose and Docked Pose.

**Figure S18:** The superimposition of docked pose (magenta color) over the crystal pose (green color) for **a)** PDB ID: 1TT1; **b)** PDB ID: 1SQN from Astex Diverse Set. The nitrogen atoms are in blue, oxygen in red, and hydrogen in white color.


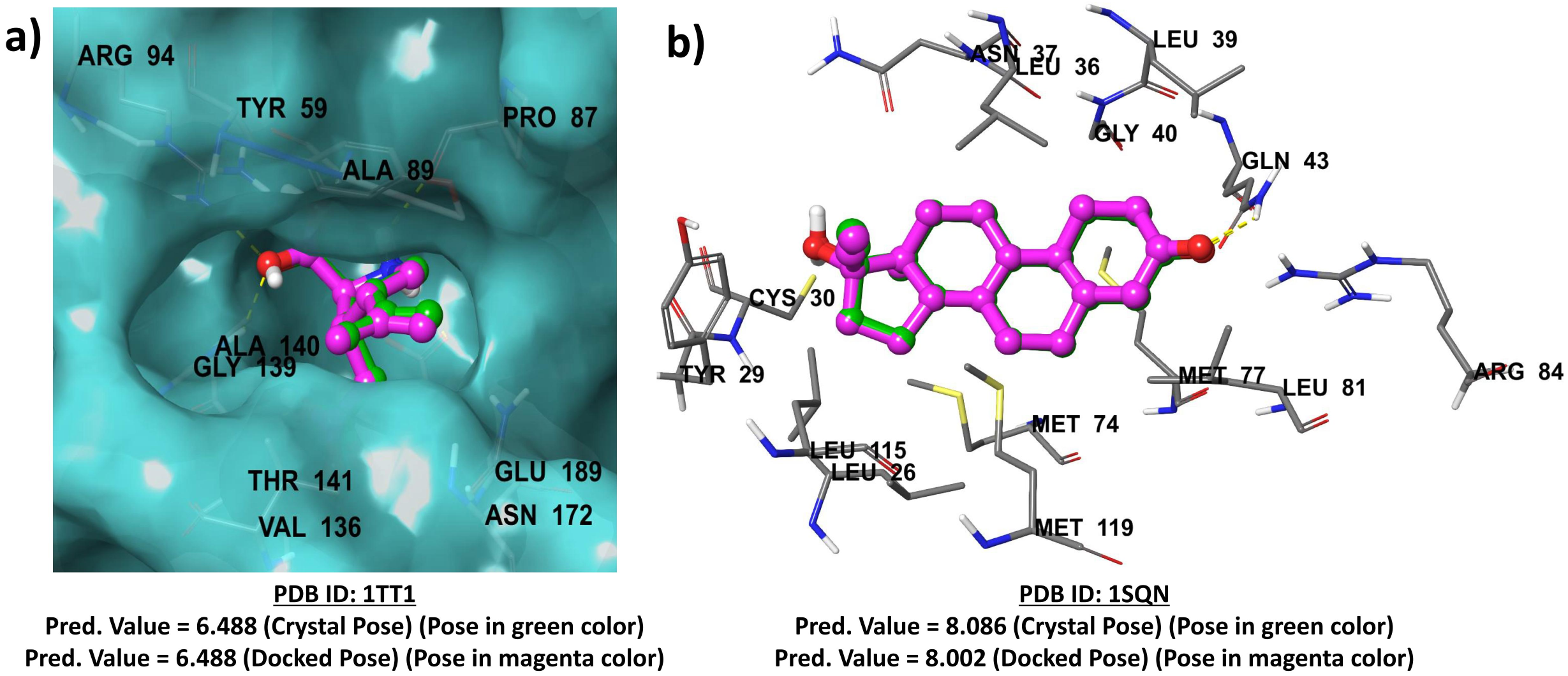


**Table S7:** Interaction Fingerprint Pattern (IFP) calculated for Crystal pose and Docked pose for PDB (**1TT1**).

| **Interaction Fingerprint Pattern** | | | | | | |
| --- | --- | --- | --- | --- | --- | --- |
| **Crystal Pose** | | |  | **Docked Pose** | | |
| **Hydrophobic** | **CZ** | **TYR_61_** |  | **Hydrophobic** | **CE1** | **TYR_61_** |
| **Hydrophobic** | **CE1** | **TYR_61_** |  | **Hydrophobic** | **CE1** | **TYR_61_** |
| **Hydrophobic** | **CG1** | **VAL_138_** |  | **Hydrophobic** | **CG1** | **VAL_138_** |
| **Hydrophobic** | **CG** | **GLU_191_** |  | **Hydrophobic** | **CG** | **GLU_191_** |
| **HBond_LIG** | **O** | **PRO_89_** |  | **HBond_LIG** | **O** | **PRO_89_** |
| **HBond_LIG** | **OE1** | **GLU_191_** |  | **HBond_LIG** | **OE1** | **GLU_191_** |
| **HBond_LIG** | **OE2** | **GLU_191_** |  | **HBond_LIG** | **OE2** | **GLU_191_** |
| **HBond_PROT** | **N** | **ALA_91_** |  | **HBond_PROT** | **N** | **ALA_91_** |
| **HBond_PROT** | **NH1** | **ARG_96_** |  | **HBond_PROT** | **NH1** | **ARG_96_** |
| **HBond_PROT** | **NH2** | **ARG_96_** |  | **HBond_PROT** | **NH2** | **ARG_96_** |
| **HBond_PROT** | **N** | **ALA_142_** |  | **HBond_PROT** | **N** | **ALA_142_** |
| **HBond_PROT** | **N** | **THR_143_** |  | **HBond_PROT** | **N** | **THR_143_** |
| **Ionic_LIG** | **OE1** | **GLU_191_** |  | **Ionic_LIG** | **OE1** | **GLU_191_** |
| **Ionic_LIG** | **OE2** | **GLU_191_** |  | **Ionic_LIG** | **OE2** | **GLU_191_** |

**Note:** Boldface represents the common interaction found in Crystal Pose and Docked Pose.

**Table S8:** Interaction Fingerprint Pattern (IFP) calculated for Crystal pose and Docked pose for PDB (**1SQN**).

| **Interaction Fingerprint Pattern** | | | | | | |
| --- | --- | --- | --- | --- | --- | --- |
| **Crystal Pose** | | |  | **Docked Pose** | | |
| **Hydrophobic** | **CD2** | **LEU_715_** |  | **Hydrophobic** | **CD2** | **LEU_715_** |
| **Hydrophobic** | **CD2** | **LEU_718_** |  | **Hydrophobic** | **CD1** | **LEU_718_** |
| **Hydrophobic** | **CD1** | **LEU_718_** |  | **Hydrophobic** | **CD2** | **LEU_718_** |
| **Hydrophobic** | **CB** | **LEU_718_** |  | **Hydrophobic** | **CB** | **LEU_718_** |
| **Hydrophobic** | **CD2** | **LEU_721_** |  | **Hydrophobic** | **CD2** | **LEU_721_** |
| **Hydrophobic** | **CE** | **MET_756_** |  | **Hydrophobic** | **CE** | **MET_756_** |
| **Hydrophobic** | **SD** | **MET_756_** |  | **Hydrophobic** | **SD** | **MET_756_** |
| **Hydrophobic** | **SD** | **MET_759_** |  | **Hydrophobic** | **CB** | **MET_759_** |
| **Hydrophobic** | **CB** | **MET_759_** |  | **Hydrophobic** | **SD** | **MET_759_** |
| **Hydrophobic** | **SD** | **MET_759_** |  | **Hydrophobic** | **CB** | **VAL_760_** |
| **Hydrophobic** | **CB** | **VAL_760_** |  | Hydrophobic | CD2 | LEU_763_ |
| **Hydrophobic** | **CE2** | **PHE_794_** |  | Hydrophobic | CD2 | PHE_778_ |
| **Hydrophobic** | **CD2** | **LEU_797_** |  | **Hydrophobic** | **CE2** | **PHE_794_** |
| **Hydrophobic** | **CD2** | **LEU_797_** |  | **Hydrophobic** | **CD2** | **LEU_797_** |
| **Hydrophobic** | **SD** | **MET_801_** |  | **Hydrophobic** | **CD2** | **LEU_797_** |
| **Hydrophobic** | **CD1** | **LEU_887_** |  | **Hydrophobic** | **SD** | **MET_801_** |
| **Hydrophobic** | **CB** | **LEU_887_** |  | **Hydrophobic** | **CB** | **LEU_887_** |
| **Hydrophobic** | **CD1** | **TYR_890_** |  | **Hydrophobic** | **CD1** | **LEU_887_** |
| **Hydrophobic** | **CE1** | **TYR_890_** |  | **Hydrophobic** | **CE1** | **TYR_890_** |
| **Hydrophobic** | **CB** | **CYS_891_** |  | **Hydrophobic** | **CD1** | **TYR_890_** |
| **Hydrophobic** | **SG** | **CYS_891_** |  | **Hydrophobic** | **SG** | **CYS_891_** |
| **Hydrophobic** | **CE** | **MET_909_** |  | **Hydrophobic** | **CB** | **CYS_891_** |
| **HBond_PROT** | **NE2** | **GLN_725_** |  | **Hydrophobic** | **CE** | **MET_909_** |
|  |  |  |  | **HBond_PROT** | **NE2** | **GLN_725_** |

**Note:** Bold face represents the common interaction found in Crystal Pose and Docked Pose.

**Figure S19:** The superimposition of docked pose (magenta color) over the crystal pose (green color) for **a)** PDB ID: 2Y5H; **b)** PDB ID: 1W4O from CASF-2016 Set. The nitrogen atoms are in blue, oxygen in red, and hydrogen in white color.


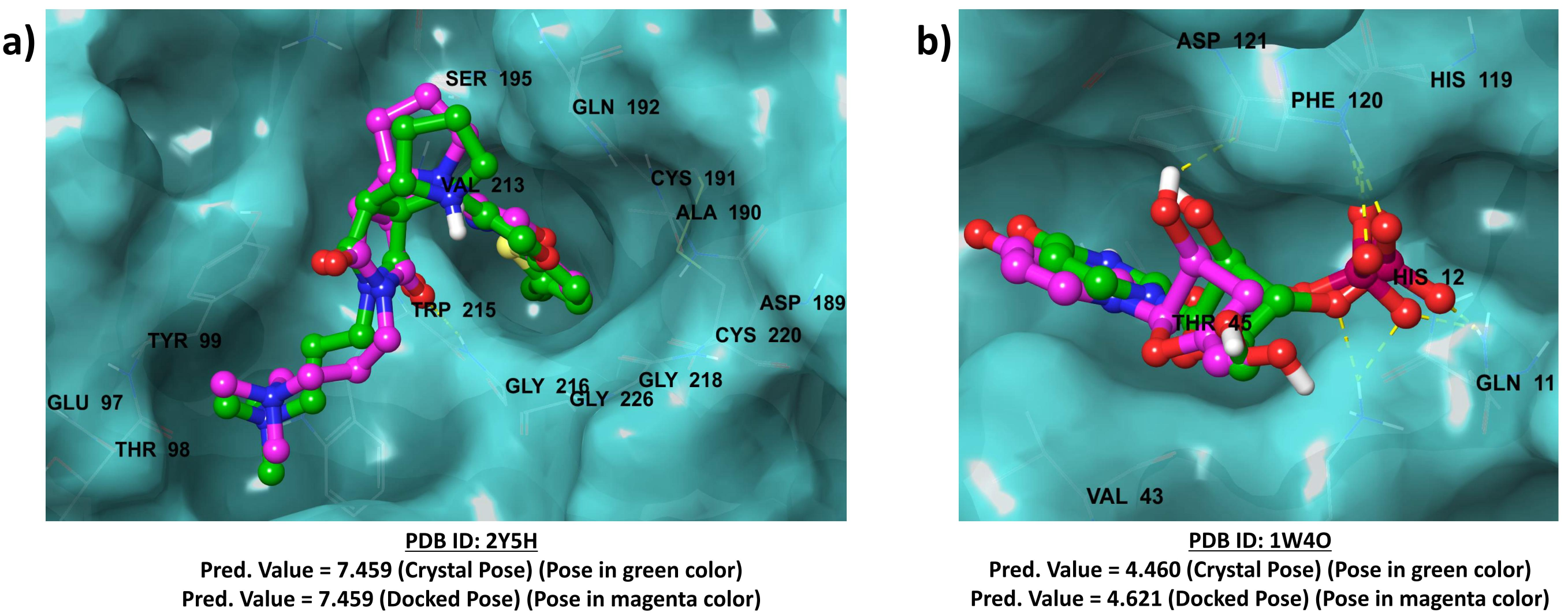


**Table S9:** Interaction Fingerprint Pattern (IFP) calculated for Crystal pose and Docked pose for PDB (**2Y5H**).

| **Interaction Fingerprint Pattern** | | | | | | |
| --- | --- | --- | --- | --- | --- | --- |
| **Crystal Pose** | | |  | **Docked Pose** | | |
| **Hydrophobic** | **CZ** | **TYR_99_** |  | **Hydrophobic** | **CZ** | **TYR_99_** |
| **Hydrophobic** | **CZ** | **TYR_99_** |  | **Hydrophobic** | **CE2** | **TYR_99_** |
| **Hydrophobic** | **CB** | **ALA_190_** |  | **Hydrophobic** | **CB** | **ALA_190_** |
| **Hydrophobic** | **CG** | **GLN_192_** |  | **Hydrophobic** | **CG** | **GLN_192_** |
| **Hydrophobic** | **CG1** | **VAL_213_** |  | **Hydrophobic** | **CG1** | **VAL_213_** |
| **Hydrophobic** | **CG1** | **VAL_213_** |  | **Hydrophobic** | **CG1** | **VAL_213_** |
| **Hydrophobic** | **CB** | **TRP_215_** |  | **Hydrophobic** | **CE3** | **TRP_215_** |
| **Hydrophobic** | **CZ** | **TYR_228_** |  | **Hydrophobic** | **CZ** | **TYR_228_** |
| **HBond_PROT** | **N** | **GLY_216_** |  | **HBond_PROT** | **N** | **GLY_216_** |

**Note:** Boldface represents the common interaction found in Crystal Pose and Docked Pose.

**Table S10:** Interaction Fingerprint Pattern (IFP) calculated for Crystal pose and Docked pose for PDB (**1W4O**).

| **Interaction Fingerprint Pattern** | | | | | | |
| --- | --- | --- | --- | --- | --- | --- |
| **Crystal Pose** | | |  | **Docked Pose** | | |
| **HBond_LIG** | **OG1** | **THR_45_** |  | **HBond_LIG** | **OG1** | **THR_45_** |
| **HBond_PROT** | **NE2** | **GLN_11_** |  | HBond_LIG | O | PHE_120_ |
| **HBond_PROT** | **NZ** | **LYS_41_** |  | **HBond_PROT** | **NE2** | **GLN_11_** |
| HBond_PROT | N | THR_45_ |  | **HBond_PROT** | **NZ** | **LYS_41_** |
| **HBond_PROT** | **ND1** | **HIS_119_** |  | **HBond_PROT** | **ND1** | **HIS_119_** |
| **HBond_PROT** | **N** | **PHE_120_** |  | **HBond_PROT** | **ND1** | **HIS_119_** |
| **Ionic_PROT** | **NZ** | **LYS_41_** |  | **HBond_PROT** | **N** | **PHE_120_** |
|  |  |  |  | **HBond_PROT** | **NZ** | **LYS_41_** |
|  |  |  |  |  |  |  |

**Note:** Boldface represents the common interaction found in Crystal Pose and Docked Pose.

**Figure S20:** The superimposition of crystal and docked pose for **PDB ID: 2JDM**. **a)** The crystal pose (cyan) with docked pose (green color): RMSD = 0.376; **b)** The crystal pose (cyan) with docked pose (purple color): RMSD = 3.53; **c)** The crystal pose (cyan) with docked pose (yellow color): RMSD = 3.043.


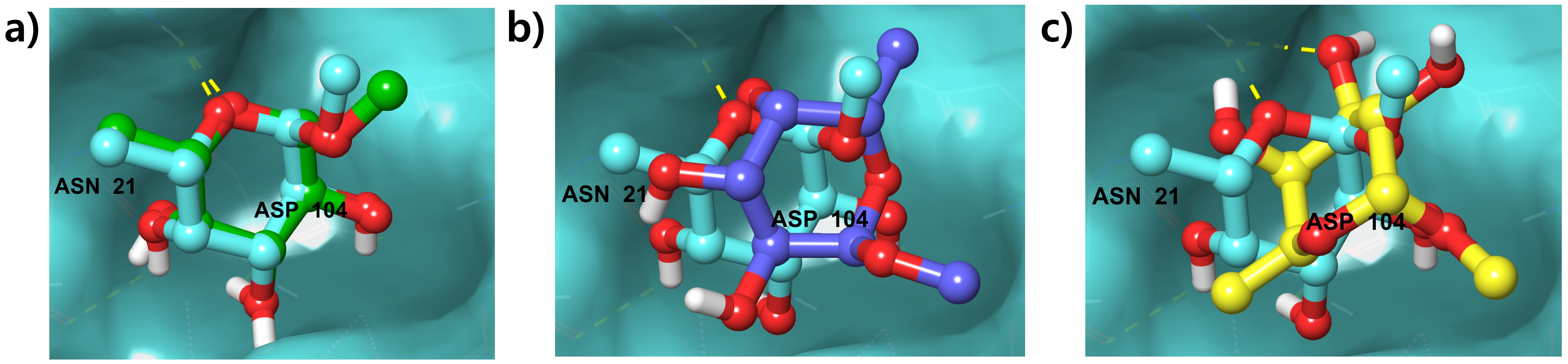


**Table S11:** Interaction Fingerprint Pattern (IFP) calculated for Crystal pose and two Docked pose for PDB (**2JDM**).

| **Interaction Fingerprint Pattern** | | | | | | | | | | | | | | |
| --- | --- | --- | --- | --- | --- | --- | --- | --- | --- | --- | --- | --- | --- | --- |
| **Crystal Pose** | | |  | **Docked Pose (RMSD = 0.376)** | | |  | **Docked Pose (RMSD = 3.53)** | | |  | **Docked Pose (RMSD = 3.043)** | | |
| **Hydrophobic** | **CB** | **ALA_22_** |  | **Hydrophobic** | **CB** | **ALA_22_** |  | **Hydrophobic** | **CB** | **ASP_99_** |  | **Hydrophobic** | **CB** | **ASP_99_** |
| **Hydrophobic** | **CB** | **SER_23_** |  | **Hydrophobic** | **CB** | **SER_23_** |  | **Hydrophobic** | **CB** | **SER_23_** |  | **Hydrophobic** | **CB** | **ALA_23_** |
| **Hydrophobic** | **CG2** | **THR_45_** |  | **Hydrophobic** | **CG2** | **THR_45_** |  | **Hydrophobic** | **CG2** | **THR_98_** |  | **HBond_PROT** | **N** | **SER_23_** |
| **HBond_LIG** | **OD1** | **ASP_104_** |  | **HBond_LIG** | **OD1** | **ASP_104_** |  | Hydrophobic | CB | ASP_99_ |  | **HBond_PROT** | **N** | **SER_23_** |
| **HBond_LIG** | **OD2** | **ASP_104_** |  | **HBond_LIG** | **OD2** | **ASP_104_** |  | **HBond_PROT** | **N** | **SER_23_** |  |  |  |  |
| **HBond_LIG** | **OD1** | **ASP_104_** |  | **HBond_LIG** | **OD1** | **ASP_104_** |  |  |  |  |  |  |  |  |
| **HBond_LIG** | **O** | **GLY_114_** |  | **HBond_LIG** | **O** | **GLY_114_** |  |  |  |  |  |  |  |  |
| **HBond_PROT** | **N** | **SER_23_** |  | **HBond_PROT** | **N** | **SER_23_** |  |  |  |  |  |  |  |  |

**Note:** Boldface represents the common interaction found in Crystal Pose and Docked Pose.

**Figure S21:** The superimposition of crystal and docked pose for **PDB ID: 3VH9**. **a)** The crystal pose (cyan) with docked pose (green color): RMSD = 0.531; **b)** The crystal pose (cyan) with docked pose (purple color): RMSD = 2.621; **c)** The crystal pose (cyan) with docked pose (yellow color): RMSD = 4.27. (Protein surfaces were hide for better view)


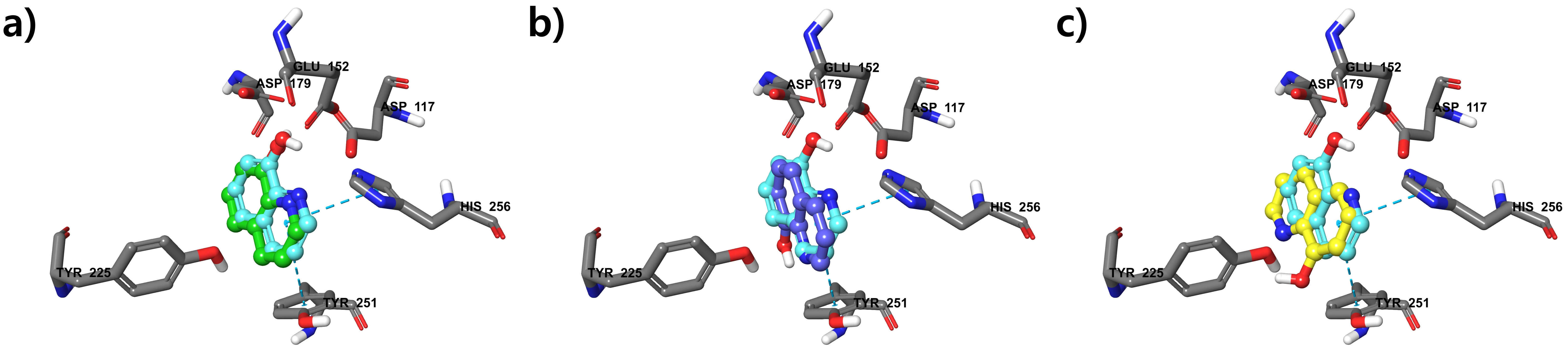


**Table S12:** Interaction Fingerprint Pattern (IFP) calculated for Crystal pose and two Docked pose for PDB (**3VH9**).

| **Interaction Fingerprint Pattern** | | | | | | | | | | | | | | |
| --- | --- | --- | --- | --- | --- | --- | --- | --- | --- | --- | --- | --- | --- | --- |
| **Crystal Pose** | | |  | **Docked Pose (RMSD = 0.531)** | | |  | **Docked Pose (RMSD = 2.621)** | | |  | **Docked Pose (RMSD = 4.27)** | | |
| **Hydrophobic** | **CB** | **ASP_179_** |  | **Hydrophobic** | **CB** | **ASP_179_** |  | **Hydrophobic** | **SD** | **MET_180_** |  | **Hydrophobic** | **SD** | **MET_180_** |
| **Hydrophobic** | **SD** | **MET_180_** |  | **Hydrophobic** | **SD** | **MET_180_** |  | **Hydrophobic** | **CD1** | **ILE_255_** |  | **Hydrophobic** | **SD** | **MET_180_** |
| **Hydrophobic** | **CB** | **CYS_227_** |  | Hydrophobic | SG | CYS_223_ |  | **Hydrophobic** | **CD1** | **ILE_255_** |  | **Hydrophobic** | **SG** | **CYS_227_** |
| **Hydrophobic** | **CE** | **MET_242_** |  | **Hydrophobic** | **SG** | **CYS_227_** |  |  |  |  |  | **Hydrophobic** | **CE** | **MET_242_** |
| **Hydrophobic** | **CD1** | **ILE_255_** |  | **Hydrophobic** | **CB** | **CYS_227_** |  |  |  |  |  | **Hydrophobic** | **CD1** | **ILE_255_** |
| HBond_LIG | OE2 | GLU_152_ |  | **Hydrophobic** | **CE** | **MET_242_** |  |  |  |  |  | HBond_PROT | OD1 | TYR_225_ |
|  |  |  |  | **Hydrophobic** | **CD1** | **ILE_255_** |  |  |  |  |  |  |  |  |
|  |  |  |  | HBond_LIG | OD1 | ASP_117_ |  |  |  |  |  |  |  |  |
|  |  |  |  | HBond_LIG | OD1 | ASP_179_ |  |  |  |  |  |  |  |  |

**Note:** Boldface represents the common interaction found in Crystal Pose and Docked Pose.

**Table S13:** The Experimental and Predicted Values for Crystal and Docked pose from selected PDBs.

| **PDB ID** | **POSE** | **RMSD** | **Exp. (pKd)** | **Pred.(pKd)** |
| --- | --- | --- | --- | --- |
| **2JDM** | **Crystal** | **-** | **5.4** | **5.739** |
|  | **Docked_pose01** | **0.376** |  | **5.739** |
|  | **Docked_pose02** | **3.530** |  | **4.869** |
|  | **Docked_pose03** | **3.043** |  | **4.846** |
| **3VH9** | **Crystal** | **-** | **6.2** | **4.362** |
|  | **Docked_pose01** | **0.531** |  | **4.579** |
|  | **Docked_pose02** | **2.621** |  | **3.963** |
|  | **Docked_pose03** | **4.270** |  | **4.199** |

**Table S14:** The Performance Comparison Between SMPLIP Feature and PLEC Feature.

| **SETS**^a^ | **IFP+Frag Features ^b^ (No: 2,422)** | | | | | | **PLEC Features ^c^ (No: 65,536)** | | | |
| --- | --- | --- | --- | --- | --- | --- | --- | --- | --- | --- |
|  | **SMPLIP-RF** | | | **SMPLIP-Linear** ^d^ | | | **PLEC-RF** | | **PLEC-Linear** | |
|  | **RMSE** | **PCC** | **p-value** | **RMSE** | **PCC** | **p-value** | **RMSE** | **PCC** | **RMSE** | **PCC** |
| Core Set-2013 | 1.489 | 0.771 | 8.71e-37 | 1.652 | 0.681 | 7.82e-26 | 1.547 | 0.758 | 1.495 | 0.755 |
| CASF-2016 | 1.643 | 0.775 | 1.10e-25 | 1.584 | 0.753 | 1.39e-23 | 1.412 | 0.800 | 1.279 | 0.822 |
| ASTEX | 1.177 | 0.724 | 0.002 | **1.703** | 0.482 | 0.069 | 1.412 | 0.606 | **1.989** | 0.557 |

^a^The benchmark data were core set of PDBbind 2013, CASF-2016, and ASTEX. ^b^Learning parameters were identical (between SMPLIP and PLEC) to compare the performance of two type features. The learning parameters were chosen based on ‘PLECScore.py' of ODDT so that they are the optimal for PLEC and not for SMPLIP-linear. ^c^ The result was gained from the original research article of PLEC.
